# The Molecular and Cellular Strategies of Glioblastoma and Non-Small-Cell Lung Cancer Cells Conferring Radioresistance

**DOI:** 10.3390/ijms232113577

**Published:** 2022-11-05

**Authors:** Lina Alhaddad, Andreyan N. Osipov, Sergey Leonov

**Affiliations:** 1 School of Biological and Medical Physics, Moscow Institute of Physics and Technology, 141700 Dolgoprudny, Russia; 2 Department of Environmental Sciences, Faculty of Science, Damascus University, Damascus P.O. Box 30621, Syria; 3 State Research Center-Burnasyan Federal Medical Biophysical Center of Federal Medical Biological Agency (SRC-FMBC), 123098 Moscow, Russia; 4 N.N. Semenov Research Center of Chemical Physics, Russian Academy of Sciences, 119991 Moscow, Russia; 5 Institute of Cell Biophysics, Russian Academy of Sciences, 142290 Pushchino, Russia

**Keywords:** ionizing radiation, radioresistance, radiosensitivity, cancer stem cells, epithelial mesenchymal transition, polyploid/multinucleated giant cancer cells

## Abstract

Ionizing radiation (IR) has been shown to play a crucial role in the treatment of glioblastoma (GBM; grade IV) and non-small-cell lung cancer (NSCLC). Nevertheless, recent studies have indicated that radiotherapy can offer only palliation owing to the radioresistance of GBM and NSCLC. Therefore, delineating the major radioresistance mechanisms may provide novel therapeutic approaches to sensitize these diseases to IR and improve patient outcomes. This review provides insights into the molecular and cellular mechanisms underlying GBM and NSCLC radioresistance, where it sheds light on the role played by cancer stem cells (CSCs), as well as discusses comprehensively how the cellular dormancy/non-proliferating state and polyploidy impact on their survival and relapse post-IR exposure.

## 1. Introduction

**Figure 1:**
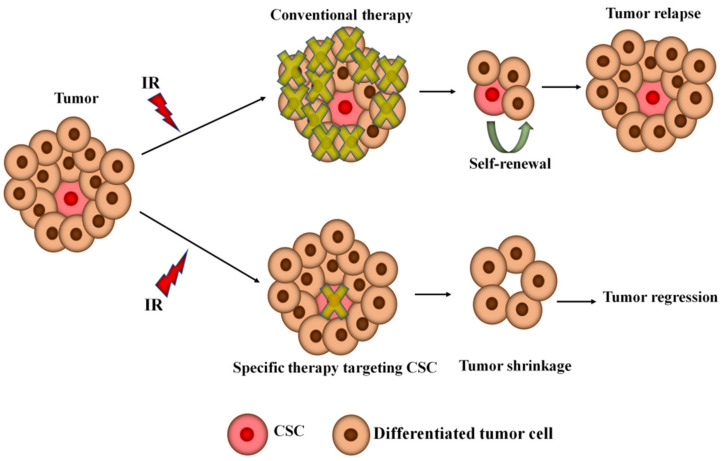
Targeting of CSCs. CSCs remaining after radiotherapy can then emerge and repopulate.

Glioblastoma (GBM; grade IV) is classified as the most aggressive, heterogeneous, and invasive primary brain tumor [1] in adults and often occurs in patients over 65 years of age [2]. GBM accounts for 15% of all brain tumors [3,4,5]. Non-small-cell lung cancer (NSCLC), a heterogeneous class of tumors, represents approximately 85% of all lung cancer diagnoses [6]. Radiotherapy represents the mainstay of therapy in patients with inoperable early-stage NSCLC [7] and GBM [8], yet the prognosis of GBM and NSCLC patients still remains poor due to a refractory response to ionizing radiation (IR) [9,10]. Therefore, a high radioresistance of these tumors still limits therapeutic success. The median survival for elderly patients with GBM remains approximately 8 months with RT alone [11], whereas the observed improvement in median survival of NSCLC time is only 5 to 7 months, and radiotherapy does not offer the possibility of a cure [12]. Tumor heterogeneity accounts for therapeutic failure [13]. Tumor recurrence occurs when therapy-surviving residual tumor cells tenaciously propagate and re-establish the tumor. Many studies have suggested that the heterogeneity of tumors can be explained not only with the characteristic of genetic instability and epigenetic changes but also with the help of cancer stem cells (CSCs) supported by antiapoptotic signaling [14,15,16]. CSCs thwart harmful insults resulting from radiotherapy due to their distinctive inherent properties of self-renewal for unlimited time, increased aggressiveness, resistance to stress, and preferential activation of the DNA damage checkpoint [10,14,17,18,19,20,21] (Figure 1). CSCs are capable of evading cell death, albeit they can become dormant for extended periods of time [22]. Plasticity of CSCs, therapy resistance, and dormancy are substantially interrelated processes [23,24]. It has been suggested that tumor progression depends profoundly on the CSC niche within it [23]. Invasion and metastasis have been known to be the main obstacles to successful therapy and are closely linked to the mortality rate of GBM [25] and NSCLC [26]. Epithelial-to-mesenchymal transition (EMT) is a very complex process regulated by several families of transcriptional factors through many signaling pathways that form a network that allows tumor cells to acquire invasive properties and penetrate the neighboring stroma, leading to the formation of a privileged microenvironment for tumor progression and metastasis [27]. EMT is hallmarked by the combined loss of epithelial cell junction proteins, such as E-cadherin, and the gain of mesenchymal markers, such as vimentin and N-cadherin [28,29]. EMT is an important inducer of the CSCs’ plasticity [30]. IR is one of the exogenous genotoxic agents that are capable of eliciting DNA damage [31]. GBM and NSCLC cells have network mechanisms in response to DNA damage, including initiation of DNA repair and, in certain cases, induction of apoptosis to annihilate dysregulated and damaged cells [32,33]. It has been reported that IR can induce proliferation arrest of tumor cells [34], which is often accompanied by senescence and/or quiescence [35,36]. The mechanisms dictating the exit from cycle arrest have considerable implications on cell fate and can thus affect the outcome of DNA damage-based tumor therapies [36]. Rarely dividing/non-proliferating tumor cells are regarded as deeply resistant to these agents, thereby causing therapeutic failure and tumor recurrence [37]. It has been suggested that tumor cells can recuperate from temporary arrest after DNA damage is repaired [36]. It has been demonstrated that IR can result in the development of polyploid tumor cells [38]. These giant cells have been reported to be adequately pliable to meet developmental tumor needs by facilitating the rapid evolution of tumors and the acquisition of therapy resistance in multiple incurable tumors [39]. Moreover, they have been shown to undergo depolyploidization [40] and produce a limited number of para-diploid clones [41,42]. It should also be noted that the radioresistance of GBM and NSCLC may be attributable to the interlink between the tumor cells and their tumor microenvironment (TME) [43,44].

## 2. Tumor Heterogeneity

**Figure 2:**
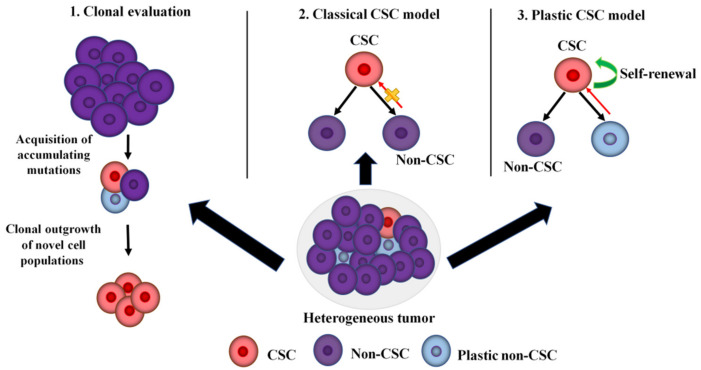
Models of tumor heterogeneity and progression of metastatic disease.

One of the most important hallmarks of GBM [45] and NSCLC [46] is cellular heterogeneity. The heterogeneity of tumor cells is a continuous cause of incomplete molecular response to radiotherapy [13,47,48] due to the diversity of tumor cells within the same tumor, leading to different responses to radiotherapy and inevitable repopulation post-IR [49,50]. Tumor heterogeneity arises from subpopulations of cells with marked genomic and/or epigenetic change and molecular signatures, a phenomenon termed intra-tumor heterogeneity [51]. Intra-tumoral heterogeneity results in the ability of a tumor to harbor anomalies in a variety of signaling pathways and cells with different levels of sensitivity to established antitumor agents [48]. Three models of tumor progression and metastasis have been postulated to explain this phenomenon—the clonal evolution, the classical CSC, and the plastic CSC models [52] (Figure 2).

The clonal evolution theory was the first model to describe a way in which tumor cells evolve progressively due to genomic instability resulting from an accumulation of successive mutations. These stochastic events generate the raw material for the clonal outgrowth of novel cell populations that can thrive under selective pressures imposed by the TME, dictating more malignant phenotypes [52,53]. Moreover, heritable changes in the epigenome permit the cells to acquire advantageous traits and to be selected in a neo-Darwinian-like evolutionary paradigm [54]. The classical CSC theory hypothesizes that tumors are immortalized by CSCs identified by their ability to initiate tumor growth, sustain self-renewal, and re-establish a heterogeneous tumor cell population [21,55,56,57]. This model focuses on the internal heterogeneity within individual clonal subsets. According to this theory, most tumorigenic cells within such a clonal population reside at the peak of the hierarchy [52]. A defining feature of this model is its unidirectional nature, whereby CSCs undergo symmetric division to replenish the CSC pool within a tumor or irreversible asymmetric division to generate more differentiated progeny with low metastatic potential. In this case, the divergent cell phenotypes are regulated by endogenous and exogenous stimuli arising in the TME. These stimuli can lead to the induction of specific growth factor and cytokine pathways that, in turn, affect subtle epigenetic changes in CSCs and their non-CSC progeny. The CSC hierarchical model has been extended to many solid human tumors, including GBM and NSCLC [58,59]. The plastic CSCs theory portrays the phenotypic conversion between the CSC and non-CSC compartments as a bidirectional process [52], highlighting an evolving mechanism by which non-CSCs can dynamically move back up the hierarchy and re-enter the CSC state due to dedifferentiation process [52]. Moreover, this model describes the basic aspects of non-CSC-to-CSC bidirectional interconversions. According to this theory, CSCs may be derived by the oncogenic transformation of normal tissue stem cells, by EMT, by mutations in key regulators of differentiation, or by a spontaneous conversion process [52]. Numerous studies have reported that IR can promote non-CSCs to obtain the phenotype and function of CSCs, so-called “awakened” CSCs [60,61].

## 3. Cancer Stem Cells Concept, History, and Properties

**Figure 3:**
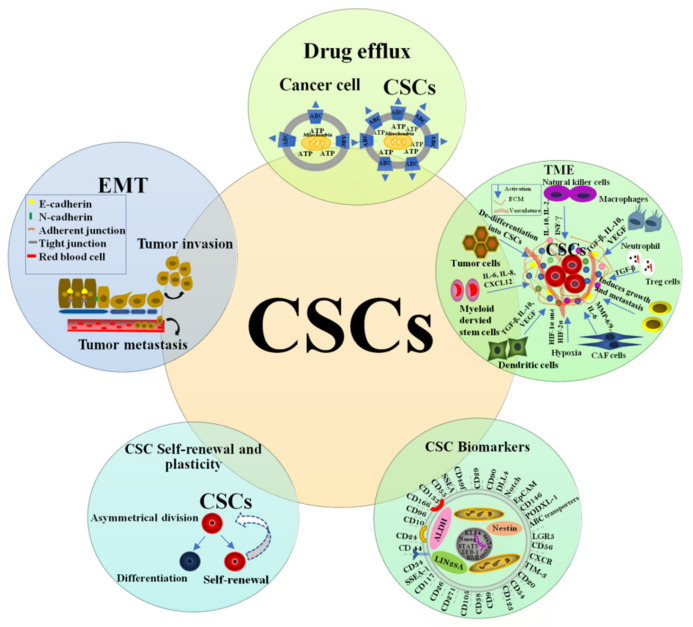
The defining properties CSCs.

Cancer stem (-like) cells (CSCs) or (CSLCs), also referred to as cancer-initiating cells (CICs), are a small subgroup of cancer cells with the capability of self-renewal and differentiation into heterogeneous tumor cells, and they have been believed to be accountable for tumor initiation, growth, and recurrence [62]. Other terms used for these cells are tumor or rescuing units, tumor-progenitor cells, and functional tumor stem cells [63]. Large-scale studies have indicated that the biological characteristics of tumors, including radioresistance, are determined by CSCs [64,65]. It has been demonstrated that CSCs can be enriched both in vitro and in vivo by IR, indicating the possibility of IR-induced generation of CSCs [66,67]. CSCs are considered to have innately higher radioresistance, invasion capability, and metastatic capacity than their differentiated counterparts [61]. Targeting CSCs in tumors may represent an effective antitumor therapeutic strategy and improve the efficacy of radiotherapy. CSCs share some, or all, properties of SCs, that endow these cells with key traits in tumorigenesis, relative quiescence, activation of survival responses, promotion of blood vessel formation, and enhanced motility [10,68]. For example, miR-200 regulates both normal stem/progenitor cells and CSCs by targeting BMI,1 which is necessitated for SC self-renewal [69,70]. Among the early investigators of the 1800s, Virchow and Cohnheim postulated the existence of CSCs that arise from what they believed to be the “activation of dormant embryonic tissue remnants” [71]. CSCs were first proposed by Fiala in 1968 [72]. Although the modern concept of SC biology was absent, a CIC was clearly hypothesized to be an SC unable to differentiate. Researchers in the 1970s advanced the theory that tissue-specific SCs might be the cells of origin for specific tumors [71]. Bayard Clarkson and coworkers identified a small subpopulation of slow-cycling cells, which they termed “dormant cells”. These cells were able to escape anti-proliferative chemotherapy and were supposed to be responsible for the relapse of lymphoblastic leukemia in adults [73,74]. The tumor colony assay in soft agar medium proposed by Hamburger and Salmon in the late 1970s introduced the concept that only a small proportion of tumor cells, yielding a plating efficiency of 0.001 to 0.1%, are tumorigenic, and the authors identified these cells as the essential target of therapy [75]. Eventually, compelling evidence for the role of CSCs in the metastatic progression of the tumors was first validated in 1997 when Dick and Bonnet isolated a set of stem cells from human acute myeloid leukemia, and the samples were capable of transferring human acute myeloid leukemia to nonobese diabetic/severe combined immunodeficient mice [56]. In this case, it was observed only that CD34+ CD38− subpopulation [56] was able to reconstitute human acute myeloid leukemia that resembled the human disease in nonobese diabetic/severe combined immunodeficient mice [56,76,77,78]. CD34+ CD38− cell fraction was shown to represent 0.1–1% of the total cells and possess the proliferative, differentiation, and self-renewal capacities expected of normal stem cells [56,76]. The first time the CSCs concept was applied to solid tumors was in 2003 by Al-Hajj and colleagues [78], when they identified that only a small subset of breast cancer cells expressing markers CD44+/CD24− possessed a marked proliferative capacity, differentiation, self-renewal, and in vivo tumor-forming ability, while the remaining bulk of cells from this tumor had none, even when injected at many-fold higher cell doses [79]. Within the same year, Singh et al. purified CD133+ CSCs from human brain tumors of different phenotypes [80]. In 2004, it was also shown that multiple myeloma contains a rare subset of cells, defined by their lack of expression of the plasma cell marker CD138, that are clonogenic in vitro and tumorigenic in vivo [81]. In 2006, a workshop of the American Association for Cancer Research discussed the rapidly emerging CSC model for tumor progression and established the definition of a CSCs as “a small subset of cells within a tumor that possesses the capacity to self-renewal and to generate the heterogeneous lineages of cancer cells that comprise the tumor” [82]. During the last decade, CSCs have been identified in many solid tumors, including lung [83], pancreas [84], prostate [85], colon [86], liver [87], blood [88], skin [89], thyroid [90], cervix [91], ovary [92], and stomach [93], as well as their functional and phenotypic features have been investigated. CSCs have been previously identified through different criteria, including increased glycolysis and glycine/serine metabolism or low concentrations of reactive oxygen species (ROS) and ATP [94]. The vast majority of studies, such as colony forming unit and marker expression, have been performed to check CSCs’ radioresistance [82,95]. CSCs have been found to display enhanced chromosomal instability, possibly highlighting a role in clonal evolution in their propagation [96]. One of the intrinsic properties of CSCs is their ability to grow in a serum-free medium supplemented with growth factors in non-adherent culture plates [97]. In general, CSCs are considered quiescent or at least slow-cycling [98,99,100,101]. These cells can stay dormant for extended periods after treatment but eventually re-enter the cell cycle, leading to tumor regrowth [102]. Furthermore, CSCs exhibit certain properties, such as long-term survival, high expression of drug efflux transporters, abnormal cellular metabolism, deregulated self-renewal pathways, acquisition of EMT phenotype [103], more efficient DNA damage repair than bulk tumor cells after IR [104] (Figure 3).

It has been reported that CSCs withstand anoikis and display immune evasion, which may result in tumor metastasis and relapse [105,106]. Complicated cellular and molecular mechanisms, such as stemness maintenance, EMT, and ROS production, are intimately involved in the process of CSC initiation and facilitation of tumor recurrence and metastasis after treatment [107,108,109,110]. Metabolic stressors, which result in numerous enlarged, elongated, and interconnected mitochondria and increase oxidative capacity and ATP production in CSCs [111]. CSCs have been reported to overexpress ROS free-radical scavengers in order to reinforce their defense against ROS-induced damage [18,112,113,114], leading to tumor radioresistance. Compared to regular stem cells, CSCs are believed to reside predominantly in niches within the TME, including stromal cells, cancer-associated fibroblasts, infiltrating immune/inflammatory cells, and vascular endothelial cells [67,115,116], to retain their unique properties [117,118]. CSCs niche facilitates their metastatic potential and preserves their plasticity [22,23]. Furthermore, a variety of conditions, such as EMT [30], hypoxia [119], inflammatory cytokines, such as IL-1 β, IL-6, C-X-C motif chemokine ligand 12 (CXCL12), and IL-8 generate cells harboring CSCs properties [120]. The acidic TME has been reported to enhance CSCs radioresistance and angiogenesis through the induction of vascular endothelial growth factor (VEGF) [121,122]. Experimental and clinical studies have indicated that CSCs also possess the ability to initiate tumor formation in the host’s body [123]. Interestingly, as few as 100 CSCs have been shown to be able to recreate tumors in nonobese diabetic/severe combined immunodeficient mice [124]. The frequency of CSC varies broadly between different tumor types, spanning from small populations of <1% in human acute myeloid leukemia and liver cancer up to 82% in acute lymphoblastic leukemia [110]. When leukemic cells were transplanted in vivo, only 1–4% of cells could form spleen colonies [67,125]. In vitro data have shown that only 1 in 1000 to 5000 lung cancer cells generates colonies in soft agar assay, indicating that not every lung cancer cell is capable of clonal tumor initiation [126].

### 3.1. Cancer Stem Cells Related Markers

A large panel of highly specific CSCs markers provides molecular targeted therapies for various tumors, using therapeutic antibodies specific to these markers [127]. These markers are categorized according to cellular localization [128,129,130,131]. Many CSCs surface membrane markers have been identified, including podocalyxin, stage-specific mouse embryonic antigen, trophoblast cell-surface antigen 2, epithelial cell adhesion molecule (EpCAM), leucine-rich repeat-containing G-protein coupled receptor 5, aldehyde dehydrogenase 1 family member A1 (ALDH1A1), CD13, CD14, CD19, CD20, CD24, CD26, CD27, CD34, CD38, CD44, CD45, CD47, CD66c, CD90, CD166, CD105, CD133, CD138, CD117/c-kit, CD151, CD166 [129], CD 29 [132], CD271 [133], CD 114 [127], CD 73 [134], integrin α6 (CD49), integrin β1(CD29), integrin β3 (CD61) [135,136], Jagged 1–2 [137], ATP-binding cassette sub-family G member (ABC) transporter family [138], neural cell adhesion molecule (NCAM) [139], and to name a few. Cell surface molecules mediate interactions between cells and their microenvironment [129]. Of note, CD44 and CD133 have been published to be the most widely used markers for isolating CSCs [129,140]. CSCs can be phenotyped by certain stemness-related transcription factors, such as (Yamanaka factors; octamer-binding transcription factor 3/4 (Oct3/4), cellular myelocytomatosis oncogene (c-Myc), SRY (sex determining region Y)-box 2 (SOX2), kruppel-like factor 4 (KLF4), Nanog, Spalt like transcription factor 4 [129], special AT-rich sequence-binding protein 2 (SATB2) [141], forkhead box M1 (FOXM1) [142], mouse nucleoside diphosphate kinase B (NME2) [143], and hypoxia-inducible factor 1-alpha (HIF-1) [144]. These transcription factors contribute to the pathologic self-renewal characteristics of CSCs [145]. Additionally, there is a number of stemness-related markers that are neither cell surface proteins nor transcription factors, including B lymphoma Mo-MLV insertion region 1 homolog (BMI1), Nestin, β-catenin, T-cell immunoglobulin and mucin-domain containing-3 (TIM-3), Musashi-1, ALDH, CXC chemokine receptors (CXCRs) [129,146], transcriptional co-activators (e.g., transformation/transcription domain associated protein TRRAP [147], yes-associated protein 1 (YAP), and transcriptional co-activator with PDZ-binding motif (TAZ) [148], and antiapoptotic genes (e.g., B-Cell Leukemia/Lymphoma 2 (BCL -2), Bcl-2-associated X protein (BAX), cellular FLICE-inhibitory protein (c-FLIP) [149,150]), Survivin [151], B-cell lymphoma-extra-large (Bcl-XL), and myeloid leukemia 1 (MCL-1) [152]. The CSCs phenotype can differ essentially between patients [153]. For example, a small subpopulation of cancer cells is present within some human breast cancers that exhibit a CD44 +/CD24 (−/low) phenotype; these tumorigenic cells have been shown to be highly enriched for CICs in xenografts compared to their counterparts [154], and display a mesenchymal phenotype in the invasive front of the tumor [155]. High CD133 expression is linked to multiple tumor recurrence, increased metastatic potential, and radioresistance [156,157,158]. It has been reported that CD44 overexpression, in particular CD44v, contributes to tumor radioresistance through the protection against ROS by stimulating the synthesis of reduced glutathione (GSH) level, a primary intracellular antioxidant [159,160]. It has been confirmed that HIF-1 α inactivates the T-cell factor-4 (Tcf-4) for direct binding to β-catenin [161], indicating a role of β-catenin–HIF-1α interaction in promoting CSCs adaptation to hypoxia after IR [161,162]. BMI1 upregulation has been found to confer radioresistance in tumors through stimulating telomerase activity and enhancing ATM recruitment to the chromatin, leading, in turn to tumor perpetuation [163]. Recent studies have confirmed that SATB2 acts as a regulator of stemness and self-renewal by augmenting the expression of pluripotency maintenance-associated transcription factors, such as SOX2, Oct4, c-Myc, KLF4, and Nanog [141,164]. ABC transporters overexpression has been reported in several tumors [165,166] and more predominantly in CSCs [137]. Multi-drug resistant proteins (MDR) have been known to mediate the transport of a variety of cytotoxic compounds out of the cells [137]. It has been shown that members of the ABC transporter superfamily have a wide range of physiological activity: (a) detoxification, permeability glycoprotein (P-gp, also known as MDR1 or ABCB1), and multi-drug resistance-associated protein 1 (MRP1, also known as ABCC1); (b) xenobiotic oxidative stress alleviation (MRPs and ABCCs); (c) cellular lipid metabolism (MDR3, ABCG, and ABCA families), and antigen presentation ATP-binding cassette subfamily-B member 2 and 3, antigen peptide transporter 1 and 2 (ABCB2/transporter associated with antigen processing 1 (TAP1) and ABCB3/TAP2) [167]. A drug-resistance characteristic of CSCs has been identified as a side population [168]. The activity of ABC transporters can be gauged by pumping out fluorescent dyes, such as rhodamine 123 and Hoechst 33342, which can be extruded by ATP-binding cassette subfamily-G member 2 (ABCG2, also known as CDw338/ breast cancer resistance protein (BCRP)) and ATP-binding cassette subfamily-B member 1 (ABCB1), respectively [169,170]. YAP/TAZ activation leads to the induction and maintenance of CSC properties in a wide range of human tumors, including GBM [171,172] and NSCLC [173]. YAP/ TAZ activation in response to IR has been shown to drive tumor growth, transformation, and metastasis [174]. Nestin overexpressing after IR has been demonstrated to correlate with the transformation of various human malignancies [175].

### 3.2. Cancer Stem Cells Signaling Pathways

CSCs exhibit deregulated activation of self-renewal pathways [103], a process that can lead to extensive cell proliferation and malignant transformation [176].

Furthermore, IR is an antitumor treatment modality that triggers multiple signal transduction networks [177]. Exploring CSC-specific signaling mechanisms and characteristics is clinically important for better-targeted radiotherapy strategies. Regulatory networks consisting of the Notch, Hedgehog (Hh), Janus kinase/signal transducer and activator of transcription (JAK-STAT), canonical and non-canonical Wingless and Int-1 (WNT/β- catenin), nuclear factor kappa-light-chain-enhancer of activated B cells (NF-κB), phosphatase and tensin homolog (PTEN) [129], transforming growth factor and mothers against decapentaplegic (TGF/SMAD), phosphatidylinositol-3-kinase (PI3K)/Akt and the mammalian target of rapamycin (mTOR), peroxisome proliferator-activated receptors (PPAR) [178,179], Hippo-YAP/TAZ [148], mitogen-activated protein kinase and extracellular signal-regulated kinase (MAPK/ERK) [180,181], and miRNAs [179] pathways have all been shown experimentally to play an essential role in regulating CSCs functions [129], controlling their properties [67], and causing radioresistance by expediting tumor recurrence [182,183]. Many of these pathways are inextricably interwoven networks of signaling mediators that feed one another, facilitating inter-pathway crosstalk [184]. For example, Notch signaling inhibition in GBM has been shown to downregulate its target Hes1, a transcriptional repressor, which in turn upregulates GLI transcription in the Hh pathway [185]. NF-κB and TGF proinflammatory signaling pathways have been shown to be activated in tumor cells in response to IR [186]. Recent studies have reported that the canonical WNT/β-catenin signaling cascade participates in the formation of tumor radioresistance by affecting the cell cycle, proliferation, apoptosis, invasion, and DNA damage repair (DDR) [187]. Hh signaling has been reported to play a fundamental role in growth, recurrence, metastasis, radioresistance, and acquisition of a CSC-like phenotype via the EMT process in various tumors [62,188]. It has been confirmed that Notch signaling is an important mediator of IR-induced EMT and responsible for IR-enhanced tumor malignancy [182,189,190,191]. Many studies have supported the role of the Hippo-YAP/TAZ signaling pathway in the induction of EMT, tumorigenesis, and chromatin remodeling. Moreover, this pathway has been shown to promote tumor radioresistance via escalating DDR [192,193,194].

### 3.3. DNA Damage Repair in Response to Ionizing Radiation

The biological consequences of IR are highly influenced by the activation of the DNA damage response mechanisms [195]. A high DDR capacity after IR has been described for CSCs in different tumor entities, including GBM and NSCLC [18,157,196]. Among the various types of DNA damages produced directly and indirectly by IR, such as single-strand breaks (SSBs), double-strand breaks (DSBs), and damaged nucleotide bases or abasic sites, DSBs represent the principal lesions that might lead to cell death or loss of reproductive capacity via activation of different pathways, such as mitotic catastrophe, apoptosis or senescence, if not adequately corrected [197,198,199,200]. It has been reported that about 1000 SSBs and 40 DSBs are produced per Gy/cell [201]. Two major mechanisms of DSBs repair have been extensively studied: error-prone non-homologous end joining (NHEJ) and error-free homology-directed recombination (HR) [202,203]. DSBs have been reported to activate three key phosphatidylinositol 3-kinase-related kinase (PIKK) family kinases: ataxia telangiectasia mutated kinase (ATM), ATM-related kinase (ATR), and DNA-dependent protein kinase (DNA-PK) [204,205]. NHEJ is initiated by the binding of the Ku70/Ku80 heterodimer to the end of the DSB [206], allowing the recruitment of catalytic subunit DNA-PKcs forming the protein complex DNA-PK [206]. DNA-bound DNA-PK is activated and phosphorylates numerous proteins, including histone H2AX (γ-H2AX) [207], Artemis [208], X-ray repair cross-complementing 4 (XRCC4), ligase IV complex [209], and XRCC4-like factor [210] that are aggregated on the site of IR-induced foci (IRIF) [211,212]. Inversely, HR uses undamaged homologous chromosome or sister chromatid as a template [213]. Consequently, this mechanism only functions in late S/G2 cell cycle phases [202] and requires the presence of breast cancer proteins [202]. NHEJ has been known to be an efficient and rapid process due to the avid end-binding ability of Ku and its high abundance [214], whereas HR is the least error-prone repair mechanism [202].

### 3.4. Cancer Stem Cells in Glioblastoma

It has been reported that a single GBM can contain heterogeneous clones of GBM stem-like cells (referred to as GSCs) with different morphologies, self-renewal, aggressive phenotype, and proliferative capacities [49,215,216,217,218,219]. The similarity of the gene-expression profiles of GSCs and normal neural stem cells (NSCs) provides support to the idea that CSCs are malignant variants of normal neural stem cells [220,221]. It has been shown that GSCs are able to grow under serum-free culture conditions identical to normal neural stem cells [222], efflux fluorescent dyes [223], and generate progeny comprised of a mixture of stem cells and more restricted non-stem cells descendants through symmetrically dividing [1,49,224]. GSCs tend to be more tumorigenic, pro-angiogenic, and radioresistant than the majority of GBM cells [10,131,225]. Recent accumulating evidence has revealed that GSCs can enhance radioresistance in GBM through activation of DNA damage checkpoint proteins, including checkpoint kinase 1 (Chk1), checkpoint kinase 2 (Chk2), ATM, structural maintenance of chromosomes (SMC1), and p53 [226]. GSCs have been reported to express cell surface CSCs markers, such as stage-specific mouse embryonic antigen, CD34, CD44, α6- integrin, CD133, L1CAM (L1 Cell Adhesion Molecule), CD54, and A2B5 [97,217,227,228,229,230,231,232,233,234,235]; cytoskeleton proteins (also referred as; intermediate filaments or nanofilaments), such as glial fibrillary acidic protein (GFAP) [236], vimentin [237,238,239], and Nestin; transcription factors, such as SOX2, Nanog, Oct3/4 [240,241], nuclear factor erythroid 2-related factor 2 (Nrf2) [241,242], oligodendrocyte transcription factor (Olig2) [243], FoxM1 [244], and zinc finger protein 281 (ZNF281) [245], POU class 3 Homeobox 2 [246], and melanocyte inducing transcription factor (MITF) [247]; posttranscriptional factors, such as Musashi 1 [248] and microRNAs [249]; polycomb (Pc) transcriptional suppressors, such as enhancer of zeste homolog 2 and BMI 1 [248]; transcriptional co-activators, such as YAP/TAZ [171] and TRRAP [250], yet no single marker is able to define a general GSC population. It has been reported that GSCs metabolism undergoes different changes than that of traditional GBM tumor cells following IR [251]. Gene expression analyses of GBM cells treated by IR have revealed that many genes are modulated after treatment [252,253,254]. These genes are closely involved in a variety of cellular processes, such as cell cycle, apoptosis, DNA replication/damage repair, cytoskeleton organization, and metabolism [255]. There is a growing body of evidence that the fraction of GSCs expressing CD133 increases after IR [80]. CD133+ GSCs have been reported to represent the cellular subpopulation that confers radioresistance and drives GBM recurrence [225,229,256]. It has been observed that injection of 10^2^ CD133+ cells forms a tumor that regenerates a phenocopy of the patient’s original tumor upon transplantation, whereas transplantation of 10^4^ CD133− cells does not lead to producing a tumor [225,229]. CD133+ GSCs radioresistance is attributed to preferential activation of the DNA damage checkpoint proteins (e.g., Chk1 and Chk2 kinases) [225,257]. It has been found that the CD133+ subpopulation is able to repair IR-induced DNA damage more efficiently and undergo less apoptosis compared to CD133− counterparts [225,258]. The CD133+ fraction among highly aggressive GBMs ranges from 0.1 to 50% [229]. It should be emphasized that some GBM tumors do not contain any CD133+ cells [259,260,261]. For example, GBM xenografts irradiated in vivo have been reported to be enriched 3–5-fold for CD133+ cells compared to untreated xenografts [225]. Bao et al. observed using a colony-forming unit assay that CD133− non-GSCs cultures irradiated at a dose of 5 Gy form fewer colonies compared to CD133+ GSC cultures [225]. Overexpression of Olig2 and CD44 after exposure to 6 Gy of cobalt-60 has been found to be related to the proliferative and invasive state of GBM [262]. Many authors have pointed out the role of α6-integrin in GBM radioresistance by increasing the efficiency of DDR [263]. IR-treated GBM cells have been reported to produce sICAM-1, resulting in a mesenchymal shift of GBM only in vivo [235,264]. Side population cells have been identified in GBM cells [168]. In 2003, Trog et al. first reported on the upregulation of the ABC-1 transporter in human GBM in response to DNA-damaging agents in an IR-dose-dependent manner. They also found that IR has a higher inducible effect on the ABC-1 expression rates depending on GBM cell density [265]. A high expression of KLF4 has been shown to promote the proliferation of the GSC population and the regrowth of GBM, even after aggressive radiotherapy [266]. An increased expression of Nrf2 post-IR has been reported to protect GBM against IR-induced oxidative stress by activating several downstream genes related to detoxification and antioxidant response, such as glutathione peroxidase and superoxide dismutase [241,267,268,269,270,271]. It has been documented that a pronounced enrichment of BMI1 after IR at the chromatin confers radioresistance in GBM through copurifying with NHEJ repair proteins, such as DNA-PK, poly [ADP-ribose] polymerase 1 (PARP-1), hnRNP U, and histone H1 in CD133+ GSCs [272]. Epidermal growth factor receptor (EGFR) and epidermal growth factor receptor variant III (EGFR^vIII^) have been shown to mediate radioresistance in GBM by maintaining EMT and activating both NHEJ and HR [273,274]. IR-induced VEGF secretion enhances the angiogenic potential of GBM [275]. Compelling preclinical proof has shown that anti-VEGF growth therapy stimulates IR-induced cell death in U-87 under normoxic and hypoxic conditions [276]. Maachani U. B. et al. demonstrated that both FOXM1 and STAT3 proteins interact together and co-localize in the nucleus under radiotherapy, facilitating the DDR processes [277]. It has been shown that GBM become more radioresistant through the overexpression of proliferating cell nuclear antigen (PCNA)-associated factor, which facilitates DNA damage bypass [278]. Accumulating evidence has pointed out the potential role of antioxidant enzymes, such as superoxide dismutase, catalase, glutathione peroxidase, glutathione reductase, in GBM radioresistance [279]. These enzymes have been reported to be activated up to 5-fold in a radioresistant variant clone isolated from a human U251 cell line compared to the parent cells after IR [279,280]. Enhanced expression of cyclooxygenase-2, also known as prostaglandin endoperoxide/H synthase 2, in GSCs has been reported to be potently involved in progressive GBM growth, as well as radioresistance [281,282]. It has been evinced that overexpression of cathepsin L, a lysosomal endopeptidase enzyme, enhances GSCs’ radioresistance through inducing expression of CD133 and phosphorylation of DNA damage checkpoint proteins [283]. Cyclin-dependent kinase 2 (CDK2) expression has been shown to be significantly enriched in GBM and is functionally required for their proliferation and growth both in vitro and in vivo [284]. CDK2 has also been to induce radioresistance in GBM cells, and its knockdown enhances cell apoptosis when combined with radiotherapy [284]. Histone deacetylase 4 and -6 have been shown to induce radioresistance in GBM by maintaining the GSC phenotype [285]. Many studies have reported that the radiosensitivity of GBM can be altered by targeting microRNAs. The expression of miR-1, miR-221/222, and miR-124 has been shown to effectively regulate IR-related signal transduction pathways in GBM [286]. Another study has revealed that miR-1, miR-125a, miR-150, and miR-425 induce radioresistance in GBM through upregulation of the cell cycle checkpoint response [287]. Many reports have stated that high Ki-67 is rigorously proportional to the high proliferative state of GBM cells [288,289]. It has been shown that the Ki-67 labeling index is significantly expressed in post-irradiated GBM cells compared to their respective pre-irradiated counterparts [290]. GSCs isolated from human LN18 cells with cell surface vimentin overexpression have been found to present 95% CD133 expression and 98% CD44 expression, suggesting that GSCs that express surface vimentin possess tumor-initiating properties [291]. Many studies have shown that vimentin regulates IR-induced migration of GBM cells [292]. Survivin has been reported to enhance GBM cell survival, regulate DSB repair capabilities, and contribute to a hypermetabolic state upon IR exposure [9,293]. Increased GFAP and insulin-like growth factor binding protein-2 (IGFBP-2) serum levels in GBM patients after radiotherapy have been shown to be correlated with the malignant degree and prognosis of GBM (wpr-669323). It has been indicated that overexpression of RCC2, a regulator of chromosome condensation 2, enhances proliferation and tumorigenesis, as well as confers radioresistance in GBM cells [294]. Alterations in several molecular and signaling pathways following IR have been shown to be closely involved in inducing radioresistance in GBM [295]. GSC functions are largely mediated by several deregulated signaling pathways, such as MEK/ERK [296], Notch [297], NF-κB [298], Hh [299], WNT/β-catenin [300], PI3K/AKT/mTOR [301], JAK-STAT [302,303], retinoblastoma protein (Rb), receptor tyrosine kinase (RTK) [304], transforming growth factor-β (TGF-β), platelet-derived growth factor [305,306], and PTEN [307], resulting in an aberrant expression of downstream signature molecules that drive radioresistance and recurrence of GBM. Marampon et al. showed that MEK/ERK pathway positively regulates HIF-1α protein activity through the sustained expression of DNA-PKcs, preserving GBM radioresistance in hypoxic conditions [296]. CD133 has been shown to promote the tumorigenic capacity of GCS by activation of the PI3K/Akt pathway by interacting with the p85 regulatory domain of PI3K [308]. It has been shown that Notch1 inhibition in GBM xenografts reduces the hypoxic fraction and delays tumor progression, suggesting a potential mechanism whereby Notch1 downregulation radiosensitizes GBM cells [309]. NF-κB signaling pathway has been found to be aberrantly activated in response to IR in GBM, where its IR-induced upregulation has been involved in GSCs maintenance, invasion, mesenchymal identity promotion, and DNA damage repair through NHEJ and HR processes [49,310,311,312,313,314,315,316]. In GBM, the most common genetic lesions, including p53, PTEN, and P16 (also known as p16^INK4A^, cyclin-dependent kinase inhibitor 2A), have been reported to regulate the DNA damage response [10]. Around 40–50% of GBM has p53 mutations [317,318]. Indeed, it has been reported that the failure of p53 to induce p21^BAX^ expression causes radioresistance in GBM [319]. Moreover, loss of PTEN contributes to an increase in the cellular motility of neural precursor cells, alteration in Chk1 localization, and genetic instability, conferring radioresistance in GBM cells [320,321]. It has been established that the radiosensitivity of GSCs can be increased by inhibiting Becline-1 and ATG5, autophagy-related proteins, indicating that the induction of autophagy contributes to radioresistance in GSC [322]. The PI3K/Akt/mTOR pathway has been suggested to play an important role in IR-induced autophagy in GBM cells [323].

### 3.5. Cancer Stem Cells in Non-Small-Cell Lung Cancer

It has been demonstrated that IR-Surviving NSCLC cells display CSCs [324]. The expression of CSC-related markers after radiotherapy is significantly correlated with a poor prognosis in patients with NSCLC [325]. There is a huge number of lung CSC (LCSC) markers, including cell surface markers, such as EpCAM [326], CD 24 [327,328], CD34 [329], CD44, CD90 [57,330], CD133 [94], CD166 [83], ALDH1 [331,332], sICAM-1 [333], ABCG2 [334], and NCAM [335]; stemness related-TFs, such as Yamanaka factors (Oct-3/4) [336], SOX-2 [337], KLF4 [338], c-Myc [339], Stabilin 2 [340], MITF [341], STAT3 [342], and HIF-1α [343]; other stemness-related markers, such as miRNAs [344], Nestin [345], BMI1 [346], Musashi-1 [347], PARP-1 [348], matrix metalloproteinases (MMPs) [349,350], VEGF, epidermal Growth Factor (EGF) [351], and chemokines (e.g., CXCL12/CXCR-4) [352]. CD24 expression in NSCLC cells has been reported to be associated with disease progression and aggressive tumor behavior [327,353]. It has been observed that CD24 is upregulated only in IR-surviving NSCLC cells [324]. It has been reported that CD44 is dramatically upregulated in IR-surviving NSCLC cells [324]. Knockdown of CD44 expression in NSCLC cells has been shown to suppress cell proliferation and colony formation in vitro [354]. Tirino et al. proposed that CD133+ cells isolated from NSCLC cells can form tumors and act as CICs [355,356]. The injection of 10^4^ lung cancer CD133+ cells in immunocompromised mice has been reported to readily generate unlimited progeny phenotypically identical to the original tumor [94]. It has been observed that A549 but not H1299 cells expand their CD133+ population after exposure to 4 Gy IR, and isolated A549 CD133+ cells have been found to be radioresistant, and this resistance has been noted to correspond with upregulated expression of DSB repair genes in A549 cells [157]. Clinically relevant IR doses (1 or 3 Gy) have been reported to induce markedly HIF-1α expression in a subset of normoxic NSCLC lines in vitro, leading to modulating the cell viability and angiogenic activity [357] through the activation of anaerobic metabolism [358]. CXCR-4 has been found to use STAT3-mediated slug expression to maintain NSCLC radioresistance [359]. Many articles have proved that angiogenic factors, such as VEGF and EGF, are correlated with tumor growth, aggressiveness, survival, disease relapse, and radioresistance in NSCLC [360]. Some studies have focused on the clinical implications of miRNAs for radiotherapy in patients with NSCLC. miRNA-210 has been found to drive radioresistance in NSCLC via promoting HIF1α-induced glycolysis [361] and regulating IR- induced DSBs repair [362]. Furthermore, miRNA-25 has been known to lessen radiosensitivity by binding the B-cell translocation gene 2 in NSCLC cells [363], whereas miRNA-1323 has been reported to decrease radiosensitivity of NSCLC by inducing the expression of protein kinase, DNA-activated, catalytic polypeptide [364]. The reduced expression of tumor protein p53-inducible protein 3, a downstream mediator of the DDR, in IR-surviving H460R cells has been shown to be greatly involved in acquired RR [365]. MMPs has found to play an undeniable role in tumor extracellular matrix (ECM) invasion [366]. It has been demonstrated that IR-surviving NSCLC cells, after exposure to 10 Gy of, show increased motility and increased expression of MMP-2/-9 [367]. It has been suggested that the detaching soluble natural killer group 2 member D ligands in NCI-H23 cells can be a result of IR-induced MMP-2 [368]. Using a 3D NSCLC model, IR with a dose of 5 Gy has been found to increase the growth of tumor tissue analogs containing CSCs and enhance the expression of cytokines (regulated upon activation, normal T cell expressed, and secreted, epithelial-neutrophil activating peptide, and TGF-α) and factors (MMP, vimentin, and tissue inhibitors of metalloproteinase (TIMP)) [369]. It has been indicated that the level of IR-induced apoptosis decreases in those NSCLC cells exhibiting BCL-2 overexpression [370]. Our previous experimental data have reported that ABCG2 expression markedly increases in multifractionated radiotherapy-surviving NSCLC cells at a total dose of 60 Gy, conferring these cells a radioresistant phenotype [170]. Silencing MITF has been reported to promote migration, invasion, colony formation, metastasis, and tumorigenesis in CL1-0, CL1-1, and CL1-5 cell lines [341]. Overexpression of SOX2 in IR-surviving NSCLC cells has been revealed to stimulate cellular migration and anchorage-independent growth, while SOX-2 knockdown has been reported to impair their growth [324,371,372]. It has been demonstrated that inhibition of Poly (ADP-ribose) polymerase-1 (PARP-1), a well-known active candidate in DNA repair, separately diminishes proliferation, migration, EMT, phosphorylation of EGFR, Akt, p38, NF-kB, and ERK in treated NSCLC with ^12^C [373,374]. Several signaling pathways, such as PI3K, MEK [375], Notch [376], Nrf2 [377], WNT/β-catenin [378], and Hh [379], have been described to regulate the behavior of LCSCs and contribute to radioresistance in NSCLC. It has been reported that PI3K kinase inhibition can play a role in boosting the radiosensitivity of NSCLC cells via immune evasion [380] and resistance to IR-induced apoptosis [381]. Overwhelming data have indicated that the dysregulated expression of the Notch signaling pathway is a frequent event in NSCLC [382,383]. It has been shown that high Notch pathway activity has an unequivocal role in survival, poor prognosis, and radioresistance in NSCLC patients through the inhibition of apoptosis, suggesting its potential as a therapeutic target [384,385]. Moreover, Notch-1 has been reported to increase NSCLC cells’ survival under hypoxic conditions by activating the insulin-like growth factor (IGF) pathway [386]. Additionally, inhibiting IR-induced Notch-1 signaling has been found to enhance the radiotherapy efficacy in H1299 and H460 cell lines [376]. It has been reported that Nrf2 expression is significantly elevated in NSCLC cells at 4 h after IR exposure [387], as well as it has been observed to regulate the cellular antioxidant system and crosstalk with Notch1 signaling pathway in response to IR [387]. It was reviewed by Heavey et al. that inhibition of the Akt/mTOR/4EBP/eIF4E pathway in NSCLC cells might result in the development of radiotherapy and overcome radioresistance [388]. The Sonic Hh-Gli pathway has been found to promote the migrative and invasive abilities of NSCLC cells by regulating EMT [389]. The aberrant activation of the WNT/β-catenin signaling in NSCLC has been reported to correlate closely with self-renewal, proliferation, tumorigenesis, progression, and radioresistance [182,390]. Increased PI3K/AKT/mTOR activation has been shown to lead to radioresistance in NSCLC cells [301]. Previously, we have reported that residual γH2AX/53BP1 foci number decreases in multifractionated radiotherapy surviving NSCLC cells compared to parental cells post-IR at extra single doses of 2, 4, and 6 Gy. Furthermore, our previous data have detected that Rad51 protein expression might play a key role in enhancing DNA DSB repair by the HR pathway in multifractionated radiotherapy survival of p53 null NSCLC cells [391].

## 4. Epithelial-to-Mesenchymal Transition and Migratory Activity in Glioblastoma and Non-Small-Cell Lung Cancer

The heterogeneity of tumor cell populations allows for the movements of either individual cells or clusters of cells. EMT, a reversible molecular and cellular process, has been invoked as a mechanism by which immotile tumor cells can acquire a migratory, invasive, and motile phenotype by attenuating adherens junction and avoiding anoikis in the TME [392,393]. The reverse process is termed mesenchymal-to-epithelial transition (MET) [394]. MET and EMT have been closely linked to the acquisition of stemness characteristics in tumorigenesis [28,30,50,395,396,397]. Tumor cells undergoing EMT have been observed to lose their apical basal cell polarity and acquire a more spindle-shaped form [398], facilitating their dissemination into the blood circulation [28]. Moreover, EMT allows tumor cells to degrade basal extracellular matrix by MMPs activation to help these transformed cells to migrate [399,400]. A partial EMT observed among tumor cells can be explicated by the different tumorigenic capabilities of tumor cells from various niches inside tumor. Tumor cells with a partial EMT have been found to be more efficient in tumor budding, invasion, and metastasis because these processes evidently require both EMT and MET [401]. Loss of expression of tight junction proteins, including E-cadherin, and upregulation of mesenchymal markers, such as N-cadherin, Vimentin, and Fibronectin, have been considered as the key molecular events of EMT [402]. EMT-inducing TFs, such as basic helix–loop–helix (bHLH) factors (e.g., E2A, an inhibitor of DNA binding (Id2, Id3), and Twist 1/2), Snail family members (e.g., Snail and Slug), finger E-box-binding homeobox factor (ZEB) family members (e.g., ZEB1/2, SMAD interacting protein-1 (SIP1)) [403,404], Goosecoid [405], ZNF281 [406], Brachyury, sine oculis homeobox homolog 1 (SIX1), transcription factor 4, FOXC2, paired related homeobox 1 [407], and others, have been demonstrated to enhance the expression of genes associated with the mesenchymal state, such as N-cadherin, Vimentin, Fibronectin, β-integrins, and ECM-cleaving proteases [407] and directly repress mediators of epithelial adhesion proteins, such as E-Cadherin, Occludins, Claudins, Desmosomes, and cytokeratins [274,403,404,408,409]. Several EMT-related signaling pathways have been identified, including signaling pathways mediated by TGF-β [410,411], bone morphogenetic proteins, WNT–β-catenin, Notch, Hh, RTKs [402], SMADs, STATs, PI3K/Akt, MAPKs [412,413], JAK/STAT3 [414], NFkB [415], Src, and Ras [407,416]. It has been reported that radiotherapy induces tumor cells to undergo EMT, resulting in marked radioresistance [417,418,419]. Moreover, EMT can be induced by TME stresses (e.g., hypoxia) [420,421]. EMT has been shown to confer tumor cells’ resistance to apoptotic stimuli [50]. EMT has known to be a motor of cellular plasticity [422] since it is accompanied by immunosuppressive TME [400,423,424], tumor-initiation potential [425], cell proliferation [426,427], and cellular senescence [428]. Furthermore, EMT has been shown to be associated with catabolic reprogramming for tumor cell survival during metabolic stress [429]. A high infiltrative nature and an increased migratory potential of GBM and NSCLC have been shown to be tightly associated with relapse [430,431]. EMT has been pointed out as one of the mechanisms that confer invasive and metastatic property to GBM and NSCLC after exposure to IR [432,433]. The most important adhesion and cell–cell contact factors, E-cadherin and β-catenin [434,435], have been found to be rarely expressed in GBM cells [50]. Sublethal doses of IR have been reported to induce cell migration and invasiveness of GBM [436]. Furthermore, multifractionated radiotherapy has been found to enhance the migratory capability of GBM cells in vivo [437]. STAT3/NF-κB and Slug signaling activation has been reported to enhance IR-induced tumor migration, invasion, and EMT properties in GBM via the upregulation of ICAM-1 [438]. NSCLC cells have been found to possess a spindle or rounded morphology and express high levels of EMT markers after prolonged exposure to IR [439]. It has been demonstrated that IR can increase EMT phenotype in NSCLC cells by regulating EMT markers via activating the JAK2 tyrosine kinase phosphorylates PAK1 (JAK2–PAK1)–Snail signaling pathway [440]. IR-surviving A549 and H460 cells at a dose of 5 Gy have been reported to express significantly higher levels of EMT markers (Snail1, Vimentin, and N-cadherin) compared to non-irradiated NSCLC cells [324]. Furthermore, it has been observed that the expression of Oct-4, SOX2, and β-catenin proteins markedly increases in adherent H460 cells maintained in a monolayer after IR at a dose of 5 Gy [324]. Our previous data have suggested that a fraction dose escalation regimen at a total dose of 60 Gy probably causes partial (or hybrid) EMT program activation in multifractionated radiotherapy surviving NSCLC cells through either Vimentin upregulation in p53null or an aberrant N-cadherin upregulation in p53wt cells [441]. Moreover, we have indicated previously that the hypofractionation regimen IR does not significantly influence horizontal 1D cell migration of multifractionated radiotherapy surviving NSCLC cells, though promoting their migration by 24 h after scratching [441].

## 5. Radiation-Induced Dormancy

Tumor niches, including metastatic, perivascular, and bone marrow cells, have been found to harbor dormant tumor cells [23]. Cellular dormancy can be reached in one of two ways: either each tumor cell arrests its cell cycling or the entire neoplasm exhibits balanced growth/apoptosis rates, but too often discussed in terms of two growth arrest mechanisms: **cellular quiescence** (G0), in which cells are in a non-proliferative/slow-cycling state with a reversible growth arrest [442,443], and **cellular senescence**, in which cell cycle arrest is largely irreversible [444,445,446,447,448]. Therefore, mechanisms of tumor relapse induced by the reactivation of dormant tumor cells depend on whether the cells became dormant via quiescence or senescence [449]. Many cues have been known to induce cellular dormancy, such as endoplasmic reticulum stress [450], angiogenic switching, immunological surveillance, anoikis, autophagy, senescence [23], TME (e.g., extracellular matrix, inflammatory signals, genetic, and epigenetics alterations) [445], and IR [24]. In tumor cells, including CSCs, dormancy has been shown to be critical for adaptation and protection against environmental stress and toxicity [451], leading to a tumor relapse [23]. However, several lines of evidence have reported that CSCs can contain heterogeneous subpopulations that either include rapid-cycling or quiescent subpopulations [452].

### 5.1. Quiescence-Associated Radioresistance

Quiescence is defined as a sleep-like state in which cells cease to divide but retain their ability to re-enter a novel cell cycle readily and rapidly [453,454]. It has been reported that tumor cells switch between phases of growth and quiescence to gain the genetic and epigenetic modifications that are imperative for survival [451]. Cellular radiosensitivity shows a heterogeneous pattern through different cell cycle phases [455,456]. Quiescent cancer cells also referred to as slow-proliferating/slow-cycling cancer cells, are considered an attractive therapeutic target for tumor treatment since they are significantly resistant to conventional radiotherapy with a higher repair capacity than cycling cells [443,457,458,459]. It has been confirmed that Ki-67 is degraded constantly in G0/G1 and accumulates in S/M phases [460]. Moreover, Ki-67 levels during G0/G1 have been found to indicate how long a cell has spent in these phases [460]. It has been shown that the more protracted a cell has spent in quiescence, the lower the Ki-67 level will be upon re-entering the cell cycle [460]. Furthermore, quiescent cancer cells have been known to display a low rate of BrdU incorporation [461] and a low ERK 1/2: p38 MAPK ratio [462]. The entrance into the quiescence state allows tumor cells to hamper stress and toxic stimuli. After repairing the cellular damage, the cells may re-enter the cell cycle upon stimulation by specific growth factors, such as E2F and CDK2 [463]. It has been shown that, after IR, GSCs are more quiescent than GBM cells that express elevated levels of glycolysis and oxidative phosphorylation, the so-called “Warburg effect”, whereas GSCs show metabolic signs of quiescence, such as a diminished non-essential amino-acid synthesis, and unchanged levels of glycolytic and oxidative metabolites [251]. Earlier studies have demonstrated that Ras-related C3 botulinum toxin substrate 2 induces aberrant proliferation of quiescent NSCLC after IR exposure to a single dose of 2 Gy via promoting JUN-B expression through megakaryoblastic leukemia 1—serum response factor (MAL-SRF) pathway [464].

### 5.2. Radiation-Induced Senescence

Therapy-induced senescence (TIS), a prolonged state of cell-cycle arrest, is reported in tumor cells treated by various therapeutic agents, including IR [465]. Senescent cells have been reported to contribute to the composition of pre-malignant [466,467,468] and malignant lesions [469]; thus, they play an indubitable role in tumor cell fate [470,471]. Cellular senescence can be triggered by short or dysfunctional telomeres, known as replicative senescence, but also prematurely, by a variety of stress signals [472]. Interestingly, IR prematurely promotes the same phenotypes as replicative senescence prior to the Hayflick limit. This process is known as stress-induced premature senescence (SIPS) [473]. Unlike in apoptosis, cells that enter senescence are not killed; they retain some metabolic activities and secretory activity despite not undergoing cell division [474]. TIS state is accompanied by induced lysosomal biogenesis [475], macromolecular [476,477] and transcriptomic alterations [478], often leading to the synthesis and secretion of a wide spectrum of mediators, a phenomenon termed the senescence-associated secretory phenotype (SASP) [479,480,481,482]. SASP-related biomarkers include: 1- soluble factors, such as growth factors (e.g., amphiregulin, epiregulin, heregulin, EGF, basic fibroblast growth factor (bFGF), FGF7, hepatocyte growth factor, VEGF, angiogenin, stem cell factor, stromal cell-derived factor-1, placental growth factor, nerve growth factor, IGFBP-2, -3, -4, -6, -7), cytokines (e.g., IL-6, IL-7, IL-1a, -1b, IL-13, IL-15), chemokines (e.g., IL-8, CXCL1, -b, -gc, monocyte chemoattractant protein 2 (MCP-2, MCP-4, MIP-1a, MIP-3a), hepatocellular carcinoma-4, Eotaxin, Eotaxin-3, epithelial neutrophil activating peptide, I-309, interferon–inducible T cell alpha chemoattractant), other inflammatory factors (e.g., interferon -γ, CXCL13, glycosylation-inhibiting factor), proteases and regulators (e.g., MMP-1, -3, -10, -12, -13, -14, TIMP-1, TIMP-2, serpin E1) [482], receptors shedding or ligands (e.g., ICAM-1, -3, osteoprotegerin, soluble tumor necrosis factor receptor I, CD263, CD120, CD95, urokinase plasminogen activator surface receptor, soluble gp130, EGF-R), nonprotein soluble factors (e.g., prostaglandin E2, nitric oxide, ROS); 2- insoluble factors (ECM) (Fibronectin, Collagens, Laminin) [482]. Upon secretion from senescent cells, these SASP factors usually act in a paracrine manner to stimulate the proliferation and/or transformation of adjacent immortalized cancer cells or even can trigger the senescence of other cells in the TME [483]. This SASP induces an EMT and invasiveness, hallmarks of malignancy, by a paracrine mechanism that depends largely on the SASP factors IL-6 and IL-8 [484]. Senescent cells have been reported to show morphological alterations, such as an enlarged, flattened, and irregular shape with increased cytoplasmic granularity bearing more vacuoles, an increase in senescence-associated -galactosidase (SA-Gal) activity, altered mitochondria in terms of both morphology (e.g., increased mass [485]) and function [486], the expression of the pH-restricted (pH 6) [487,488,489], and an altered chromatin organization known as senescence-associated heterochromatin foci (SAHF) [490] contributing to the silencing of proliferation-promoting genes, including E2F target genes, such as cyclin A [491]. Senescence-associated cell enlargement is ascribed to several mechanisms, and one of them is cellular hypertrophy, in which a cell gets bigger due to an accumulation of proteins [492]. Senescent cells also lose monolayer integrity, which may result from the downregulation of intercellular junctions [493,494]. Unfortunately, TIS is reversible state for only some subsets of the senescent cell population, leading to cellular re-proliferation and, ultimately, tumor progression [444,495,496]. For example, several studies on various tumor types, including GBM and NSCLC, have shown that therapy-induced senescent cells can re-enter the cell cycle to trigger relapse [465,496,497,498]. It has been demonstrated that, after IR, senescent non-CSCs secrete chemokines contributing to the maintenance and migration of CSCs [499]. It has been noted that the long-term G2 arrest and subsequent senescence by G2-slippage are more preponderate at a high dose of IR than at a moderate dose [500,501]. TIS has a profound influence on the radiotherapeutic outcome, particularly in multifractionated regimens where the IR dose is increased incrementally. Because every single dose of IR will convert some tumor cells into senescent cells, thus treatment may not contribute to the anticipated antitumor effect by the time a patient receives the highest doses of IR. An emerging body of evidence has also confirmed that ‘‘irreversible’’ senescence can be overcome following radiotherapy. Of note, tumor suppressor proteins, such as PTEN, p53, or hypo-phosphorylated Rb, can be used to detect cellular senescence. Even the absence of markers can be used, including the absence of Ki-67 or the lack of bromodeoxyuridine (BrdU) incorporation [483]. It has been reported that the conditional expression of p53, p16^INK4A^, or p21 ^waf1^/^cip1^ alone in neoplastic cell lines results in irreversible growth arrest and senescence phenotype [502]. It is worth emphasizing that at present, the list of reliable markers reflecting the causes and features of cellular senescence in vitro and in vivo goes far beyond SA-β-Gal expression, including high expression levels of the CDK inhibitor, p16^INK4A^, p21^cip1^ [503], SASP [484,504], Lipofuscin [505], Lamin B1 downregulation [488], γ-H2A.X, as well as SAHF [473]. The radiation-induced senescent cancer cells express SASP that is required for triggering the proliferation, invasion, and migration of surrounding cells in vitro [484,506]. Tumor cells can undergo senescence following radiotherapy in vitro and in vivo [507]. Further investigations have revealed that the increase in SASP-expressing senescent GBM cells is likely one of the main reasons for GBM recurrence post-radiotherapy [482,508]. It has been observed that ^137^Cs γ-ray IR at single acute doses (0, 2, 5, 10, and 20 Gy) renders 17–20% of U87MG and LN229 cells dead but gives rise to 60–80% of growth-arrested GBM cells with elevation of senescence markers, such as SA-β-Gal+ cells, H3K9me3+ cells, and p53-p21^cip1^ + cells. Furthermore, it has been reported that 24 h after IR with a total dose of 20 Gy, expression of SIPS factors, such as IL6, IL8, IL1α, IL1β, chemokine (C-C motif) ligand 2 (CCL2), CXCL1, SASP mRNAs, and p21^cip1^, increases significantly in irradiated U87MG and LN229 cells compared to non-irradiated counterpart cells. It has been suggested that IR likely triggers SASP induction in GBM cells via activation of NFκB signaling [508]. Upon treatment by IR, the primary response of GBM cells has been reported to be proliferation arrest. The arrested GBM cells then undergo premature senescence within 4–8 days following IR as alternative responses to apoptosis [509]. It has been demonstrated that PARP-1 activity in GBM during radiotherapy is required for residual GBM cells to escape from TIS [498]. IR has been shown to induce primarily premature senescence rather than apoptosis in human NSCLC in a dose-dependent manner [510]. The antitumor effect of IR doses (0–6 Gy) has been reported to correlate well with IR-induced premature senescence, as evidenced by increased SA-β-Gal staining, decreased BrdU incorporation, and elevated expression of p16^INK4A^ in irradiated NSCLC [510]. Previous studies have shown that IR-induced senescence in NSCLC cells is associated with p53 and p21 expression [471,511]. It has been demonstrated that IR induces the expression of phosphorylated p53 and p21 in a dose-dependent manner in H460 cells [510]. It has been reported that escape from TIS in both a p53 null NSCLC in vitro and in primary tumors is due to overexpression of CDK1 [512] and survivin [513] and that aberrant expression of CDK1 promotes the formation of polyploid senescent cells, which are an important intermediary through which escape preferentially occurs [495]. It has been demonstrated that the concurrent radiotherapy with the blockade of DNA-PK and PARP-1 enhances the senescence of irradiated H460 cells in vitro and in vivo further than that accomplished with IR alone [514].

## 6. Polyploid Giant Cancer Cells/Multinucleated Giant Cancer Cells in Glioblastoma and Non-Small-Cell Lung Cancer

**Figure 4:**
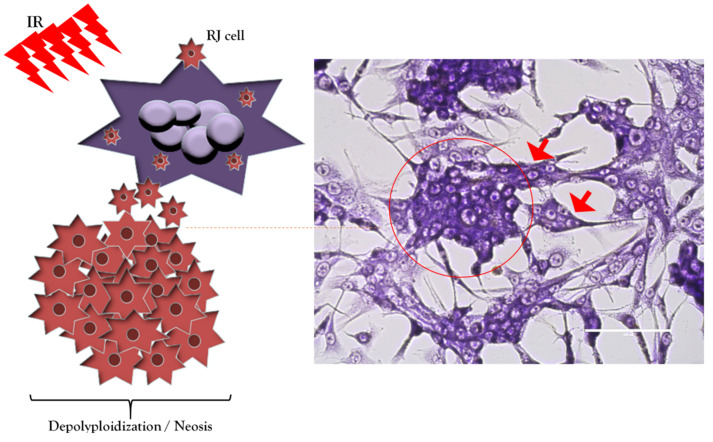
PGCC/MNGC-cell-derived RJ cells in X-ray irradiated GBM U-87 cells.

Aneuploidy is a ubiquitous characteristic of tumors. Over 90% of human solid tumors are aneuploid [515]. A large amount of data has been provided irrefutable evidence that IR can activate cell cycle checkpoints that inhibit entrance into or progression through mitosis [516,517]. The frequency of polyploid giant cancer cells (PGCCs) and multinucleated giant cancer cells (MNGCs) have been reported to be positively correlated with high tumor recurrence, malignancy grade [518,519], poor prognosis, and resistance to tumor therapy [520,521,522,523,524,525]. PGCCs/MNGCs have been shown to contribute to solid tumor heterogeneity [526] and to be an integral part of the tumor cell life cycle [526]. PGCCs/MNGCs are not dormant as formerly thought [13,526,527]. It has been revealed that tumor cells can escape cell death following IR by endopolyploidization [41], as well as PGCCs/MNGCs have been observed to be more radioresistant than their diploid counterparts [528,529]. Polyploid tumor cells formed through IR-induced mitotic catastrophe have been demonstrated to be able to survive long enough to establish a growing population of cells (for weeks) post-IR [530,531,532,533]. PGCCs/MNGCs have been described as flattened tumor cells with extremely enlarged or multiple nuclei with an elevated genomic content when compared to other tumor cells in the same tumor [525,534,535], which confer on them the ability to generate the functions of different cell types via genetic and epigenetic modifications [40]. These cells have been shown to cease to proliferate or proliferate very slowly such that they are often considered as dead cells in the traditional colony formation assay, also referred to as “clonogenic survival”, the gold standard for assessing radiosensitivity of human cells in vitro [40,42]. Several studies involving various tumor cell types have demonstrated that these giant cells are highly adaptable to hypoxic stress and acquire a mesenchymal phenotype with increased expression of CSCs markers, such as CD44, CD133, Oct4, stage-specific embryonic antigen-1 (SSEA-1), NANOG, and SOX2 [526] and ZEB1 [39,522,526,536,537,538]. The main mechanisms responsible for the formation of PGCCs/MNGCs appear to be associated with cell fusion [539], endoreplication/mitotic bypass [540,541], cytokinesis failure [540,541], and cell cannibalism by entosis [542]. Of note, polyploidy can either be reversible and irreversible [543]. Irreversible polyploidy has been known to occur through DNA re-replication in the absence of mitosis and can reach very high levels of genome duplication up to several thousand or more [544], while PGCCs/MNGCs, which typically do not exceed 32 n, can revert to mitosis and initial para-diploidy [38,533,545]. While most of these cells will undergo cell death following mitotic catastrophe [546], some of them can release continuously small rapidly proliferating viable para-diploid tumor cells termed “Raju, RJ” with extended mitotic life span via “neosis” or “de-polyploidization” [514,546,547,548,549]. Neosis, a novel manner of cell division in tumors, was first reported by Sundaram et al. in 2003 [548]. This peculiar parasexual pattern of somatic reduction division of PGCCs/MNGCs is characterized by karyokinesis through efficient mechanisms, such as nuclear budding/bursting, giving rise to small daughter nuclei; these nuclei then acquire cytoplasm, desperate from the giant mother cells, and exhibit long-term proliferation. The authors referred to this process as the “giant cell cycle” [550]. Additionally, it has been shown that this process involves nuclei remodeling, telomere clustering, and chromosome double loop formation [530,533]. The giant cell cycle is controlled by key regulators of stemness (e.g., Oct4), mitosis (e.g., cyclin B1 and aurora B kinase), and meiosis (e.g., MOS) [537,543,551]. Reduction division of polyploidy cells has been shown to express features of meiotic divisions in a disordered fashion and contribute to genetic diversity rendering tumor cells more apt to survive following antitumor treatments [530,552]. Several studies have demonstrated that RJ cells give rise to transformed cell lines with genomic instability and also display a phenotype and transcriptome different from the mother cell [548]. Compared to diploid cancer cells, RJ cells have been shown to express fewer epithelial markers and gain a mesenchymal phenotype [524]; thus, these cells can stimulate migration, invasion, and anchorage-independent growth [548]. Although their depolyploidization processes can occur at any time post-treatment, it can take several weeks or months until a stable population of daughter cells appears [42,526,553]. It has been found that the retreatment of the recovered cells causes the same process again [41]. In addition, the newly formed RJ cells have been reported to play a role in self-renewal in tumors [554,555] due to their stem-like traits [526,536,556]. Diaz-Carballo et al. [557] documented that PGCCs/MNGCs can confer the surrounding cells’ stemness properties through lateral transfer of a sub-genome, in which PGCCs/MNGCs form intra-cytoplasmic daughter cells that express increased levels of CSCs markers and then transmit into neighboring cells via cytoplasmic tunnels [40]. It has been demonstrated that p53 deficiency is permissive for multipolar and asymmetric divisions of tetraploid cells, resulting in ample alterations in cell cycle progression and formation of aneuploid cells [41,552,553]. It has been shown that the response of radioresistant p53 mutated tumors to genotoxic damage is characterized by a failure to arrest in the G1 phase and induction of mitotic catastrophe [549]. Data on the enrichment of PGCCs/MNGCs following IR exposure were published first by Puck and Marcus for the human HeLa cervical carcinoma cell lines in 1956 [558]. The authors observed using CFA that a large proportion of HeLa cells lost their ability to produce macroscopic colonies (≥50 cells) within 9 days post-IR at a single dose of 7 Gy [558]. Furthermore, they also showed that these cells remained metabolically active for long times post-IR (e.g., 3 weeks), indicating their ability to change medium pH, if the medium was periodically changed. Genotoxic treatment-induced PGCCs/ MNGCs have been demonstrated to exhibit increased resistance to DNA damage [41,42,548,553]. Increasing evidence has shown that curbing the genotoxic insults is clearly linked to reversible polyploidy, which itself is associated to a stemness phenotype induction [543]. It has been reported by Weihua et al. that grafting only a single MNGC was sufficient to produce metastatic lung tumors in murine fibrosarcoma model [13]. We have previously demonstrated that hypofractionation regimen causes an increase in the proportion of polyploidy in both p53-null and p53-wt radiotherapy surviving NSCLC sublines compared to parental cells [441]. They provide a powerful survival advantage to cells carrying DNA damage [543]. Polyploidization cycle has been shown to continue on days 3–5, ultimately leading to a polyploidization phase (8–32n). On days 5–6 post-IR, the switch from polyploidization to ploidy reduction divisions emerges [41]. Mirzayans et al. found that the lowest frequency of PGCCs/MNGCs in low-passage primary GBM cell lines was 1 in 20 cells (~5% of total cells) [40]. Based on such observation, the authors evaluated that each ~1 cm^3^ of brain tumor contains at least 5 million of MNGCs/PGCCs [40]. It has been pointed out that PGCCs/MNGCs are not pre-existing giant cells from the parent population but generate via IR-induced homotypic cell fusion among radioresistant GBM cells [547]. Data from our very recent study have suggested the significance of TP53wt/PTENmut status in the maintenance of in vitro cycling and migration of radioresistant GBM cells to produce a high number of PGCCs/MNGCs in response to therapeutic IR doses (2–6 Gy). Our current general data have revealed that some TP53wt/PTENmutGBM cells-derived PGCCs/MNGCs can generate RJ cells and finally form large colonies 24 h post-IR (Figure 4). In addition, this work has indicated that differences in the proliferative activity, colony formation, and GBM cell lines radioresistance seem to be related to aneuploidy and neosis and not to a mutant p53 expression (Lina Alhaddad et al., 2022, unpublished data).

## 7. Tumor Microenvironment (TME)

Because the sites of recurrence in GBM and NSCLC following radiotherapy are located around the radiation-treated areas, it has been suggested that IR may contribute to the induction of the TME [559,560]. The TME is comprised of a variety of cell types, including proliferating tumor cells, non-neoplastic stromal cells, endothelial cells (EC), ECM, blood vessels, infiltrating immune/inflammatory cells, cancer-associated fibroblasts, myeloid suppressor cells (MSCs), regulatory T cells (T_reg_), tumor-associated macrophages (TAMs) [137], and tumor-infiltrating lymphocytes (TILs). Furthermore, the TME also consists of various immunosuppressive factors released by all cell types within the tumor to support its growth, progression, and malignancy, such as prostaglandin E2 [561], adenosine [562], NF-κB, tumor necrosis factor-alpha (TNF-α) [563], tumor-associated gangliosides [564], immunosuppressive cytokines (for example TGF-β [565], IL-8 [566], and others [567]. All these networks of various cells and biomolecules in the TME have been shown to contribute to the radiation response [568]. The defective function of dendritic cells (DCs) has been known to represent one of the mechanisms of tumor evasion from immune system control [569]. Natural killer cells, which mediate the innate immune system and engage in reciprocal interactions with macrophages, DCs, T cells, and endothelial cells, are conspicuously absent from most tumor infiltrates [570,571]. Recent studies have pointed to the potential of the TME to initiate SC programs. TILs, containing various proportions of CD3+CD4+ and CD3+CD8+ T cells, are usually a major component of the TME [572]. TAMs have been known to be involved in inducing angiogenesis, tumor growth, migration, metastasis, invasion, immunosuppression, and resistance against radiotherapy through secreting many inhibitory chemokines and cytokines, such as IL-6, IL-8, IL-10, IL-34, colony-stimulating factor 1 (CSF-1), tumor necrosis factor, prostaglandin E2, MMPs, and CCL2, CCL5, and CCL18 [573,574]. It has been well documented that MSCs regulate the immune response under normal physiologic conditions by interacting with various immune cells [575] and the maturation, differentiation, proliferation, and functional activation of peripheral blood mononuclear cells [576], but in the tumor, presence are subverted to induce its escape [577,578]. MSCs have been shown to be increased in the peripheral blood of patients with various tumors [579]. MSCs present in the TME have been found to promote tumor growth and suppress immune cell functions, as well as display radioprotective activity through copious production of an arginase 1, an enzyme involved in the metabolism of L-arginine, which synergizes with nitric oxide synthase to increase superoxide and nitric oxide production, blunting lymphocyte responses [580,581]. MSCs also suppress T-cell responses in the TME. Tumors release TGF-β or promote TGF-β secretion from MSCs [582]. In addition, indoleamine-2,3-dioxygenase (IDO) secreted by MSCs has been reported to be involved in the breaking down of tryptophan, an essential amino acid for differentiation and T-cell proliferation [583]. Tumors produce ample factors, including IL-6, IL-10, CSF-1, granulocyte- macrophage CSF (GM-CSF), VEGF, which elicit MSC recruitment and block lymphocyte functions, as well as DCs maturation [582]. Polymorphonuclear leukocytes have been infrequently seen in tumor infiltrates [584]. Inflammatory cells present in the TME have been reported to contribute to tumor progression [577]. Tregs are a characteristic feature of the TME and represent potent mediators of dominant self-tolerance in the periphery [585,586]. Accumulations of Tregs in the TME characterized by the expression of the forkhead/winged helix transcription factor (Foxp3) have been reported to promote tumor progression and prognosis, as well as downregulate effective antitumor immune responses in tumor-bearing hosts, thereby deterring tumor immune surveillance [587,588,589]. In the process of tumor immune escape, Tregs have been shown to suppress antigen presentation by myeloid-derived suppressor cells [590], DCs, CD4+ T helper (Th) cells and generate tumor-specific CD8+ cytotoxic T lymphocytes through TGF-β, IL-10, and IL-35 secretion (epstein-barr virus induced 3-IL-12α heterodimer) [586,591]. Treg-expressing cytotoxic T lymphocytes associated antigen 4 have been found to combine with CD80 and CD86 on the surface of DCs, leading to reduced DCs maturation [592], as well as Tregs promote the immunosuppressive capacity of myeloid-derived suppressor cells via the programmed cell death ligand 1 (PD-L1) pathway [590]. Furthermore, it has been suggested that Tregs interfere with cell metabolism mainly in two ways: (a) IL-2 deficiency in the TME, thus inhibiting the growth of effector T cells [593]; (b) CD39 and CD73, which are constitutionally expressed in human Tregs, can hydrolyze extracellular ATP or ADP into AMP and produce adenosine [594]. Several Tregs subsets have been recognized in tumors: (a) natural Tregs (nTregs_)_, which obstruct the proliferation of other T cells in the TME through contact-dependent mechanisms involving the CD95 or granzyme B/perforin pathways, and they have been found to be responsible for maintaining peripheral tolerance to self [595]; (b) inducible Tregs (iTregs) also referred to as type 1 regulatory T cells (Tr1), which are induced in the periphery following chronic antigenic stimulation in the presence of IL-10 derived from tolerogenic antigen-presenting cells [596]. Additionally, FOXP3+CD3+CD4+CD25+ phenotype has been found to occur in nTreg [597], while CD4+CD25lowCD132+TGF-β+IL-10+IL-4- phenotype has been considered to be a classical combined marker of Tr1 [586]. The GBM TME has been shown to be more immunosuppressive compared to other malignancies due to the release of potent immunosuppressive cytokines (e.g., IL-10 and TGF-β) [598]), negative regulators of effector cell functions (e.g., programmed death-ligand 1 and IDO), and oncometabolites (e.g., (R)-2-hydroxyglutarate6 and O6-methylguanine-DNA methyltransferase promoter methylation) [599]. It has been demonstrated that tumor-infiltrating neutrophils facilitate GSCs accumulation through S100A41 upregulation [600]. It has been shown that soluble factors secreted by endothelial cells maintain the self-renewal of GSCs and facilitate the initiation and growth of tumors [601]. It has been indicated that IR enhances the invasiveness of NSCLC via GM-CSF [602]. The expression level of IL-23 has been reported to be elevated in NSCLC patients after radiotherapy in response to the secretion of growth factors, signaling molecules, and anti-apoptosis factors compared to non-irradiated serum samples [333].

## 8. The Potential Treatment of Radioresistance in Glioblastoma and Non-Small-Cell Lung Cancer

Radiotherapy is a modality of oncologic treatment that can be used to treat about 50% of all cancer patients either alone or in combination with other treatment modalities such as chemotherapy, surgery, immunotherapy, and therapeutic targeting. Standard radiotherapy for GBM and NSCLC malignancies is not target-specific against them and is often not fully effective. The need to improve additional strategies for the treatment of these cancers is urgent. As mentioned previously, a major factor related to radioresistance is the existence of CSCs inside tumors, which are responsible for metastases, relapses, and radiotherapy failure. The intrinsic radioresistance of CSCs reveals the need to reassess the underlying mechanisms of the response of tumors to conventional and novel radiotherapy with a specific focus on CSCs. The identification of molecular targets that control CSCs can contribute to the development of novel chemotherapeutic drugs able to eliminate and prevent new CSCs growth in patients. This will help prevent metastasis and tumor relapse with a reduction of morbidity and toxicity, ultimately improving the outcomes in cancer patients. In order to conquer CSCs’ radioresistance to conventional radiotherapy, different strategies such as immunotherapy, gene therapy, molecular inhibition, and combination therapy have been widely investigated. Moreover, although many patients are still treated with conventional radiotherapy, other modern radiotherapy techniques have been developed, such as stereotactic body radiotherapy, hadron, and ultra-high dose-rate radiation therapy, which delivers precise high doses of radiation to target local tumors.

## 9. Conclusions and Perspectives

Collectively, the unique proprieties of CSCs, such as the ability to sustain a pool of undifferentiated stem cells through self-renewal, a high level of plasticity due to their adaptation to the TME pressures, including oxidative stress and immunosuppression, remarkable tumorigenic and metastatic capabilities, and an efficient DNA damage repair, make them the root of tumor relapse. The identification of CSCs within GBM and NSCLC may therefore be critical to hinder tumor radioresistance. IR-induced proliferation arrest and polyploidy can favor the emergence of highly tolerable stem-like phenotype and self-renewal potential in these tumors, thereby targeting quiescent cancer cells, prematurely senescent, and PGCCs/MNGCs in conjunction with radiotherapy for patients diagnosed with GBM and NSCLC may also represent an attractive avenue to circumvent their advanced malignancy and recurrence.

## Data Availability

Not applicable.

## References

[1] Berger F., Gay E., Pelletier L., Tropel P., Wion D. (2004). Development of gliomas: Potential role of asymmetrical cell division of neural stem cells. Lancet Oncol..

[2] Brandes A.A. (2003). State-of-the-art treatment of high-grade brain tumors. Semin. Oncol..

[3] Kleihues P., Louis D.N., Scheithauer B.W., Rorke L.B., Reifenberger G., Burger P.C., Cavenee W.K. (2002). The WHO classification of tumors of the nervous system. J. Neuropathol. Exp. Neurol..

[4] Keles G.E., Anderson B., Berger M.S. (1999). The effect of extent of resection on time to tumor progression and survival in patients with glioblastoma multiforme of the cerebral hemisphere. Surg. Neurol..

[5] Furnari F.B., Fenton T., Bachoo R.M., Mukasa A., Stommel J.M., Stegh A., Hahn W.C., Ligon K.L., Louis D.N., Brennan C. (2007). Malignant astrocytic glioma: Genetics, biology, and paths to treatment. Genes Dev..

[6] Gridelli C., Rossi A., Carbone D.P., Guarize J., Karachaliou N., Mok T., Petrella F., Spaggiari L., Rosell R. (2015). Non-small-cell lung cancer. Nat. Rev. Dis. Prim..

[7] Ko E.C., Raben D., Formenti S.C. (2018). The Integration of Radiotherapy with Immunotherapy for the Treatment of Non–Small Cell Lung Cancer. Clin. Cancer Res..

[8] Liu C., Sarkaria J.N., Petell C.A., Paraskevakou G., Zollman P.J., Schroeder M., Carlson B., Decker P.A., Wu W., James C.D. (2007). Combination of Measles Virus Virotherapy and Radiation Therapy Has Synergistic Activity in the Treatment of Glioblastoma Multiforme. Clin. Cancer Res..

[9] Hegi M.E., Diserens A.-C., Gorlia T., Hamou M.-F., de Tribolet N., Weller M., Kros J.M., Hainfellner J.A., Mason W., Mariani L. (2005). MGMTGene Silencing and Benefit from Temozolomide in Glioblastoma. N. Engl. J. Med..

[10] Rich J.N. (2007). Cancer Stem Cells in Radiation Resistance. Cancer Res..

[11] Perry J.R., Laperriere N., O’Callaghan C.J., Brandes A.A., Menten J., Phillips C., Fay M., Nishikawa R., Cairncross J.G., Roa W. (2017). Short-Course Radiation plus Temozolomide in Elderly Patients with Glioblastoma. N. Engl. J. Med..

[12] Wisnivesky J.P., Bonomi M., Henschke C., Iannuzzi M., McGinn T. (2005). Radiation Therapy for the Treatment of Unresected Stage I-II Non-small Cell Lung Cancer. Chest.

[13] Weihua Z., Lin Q., Ramoth A.J., Fan D., Fidler I.J. (2011). Formation of solid tumors by a single multinucleated cancer cell. Cancer.

[14] Ishii H., Iwatsuki M., Ieta K., Ohta D., Haraguchi N., Mimori K., Mori M. (2008). Cancer stem cells and chemoradiation resistance. Cancer Sci..

[15] Tu S.-M., Lin S.-H., Logothetis C.J. (2002). Stem-cell origin of metastasis and heterogeneity in solid tumours. Lancet Oncol..

[16] Passegué E., Jamieson C.H.M., Ailles L.E., Weissman I.L. (2003). Normal and leukemic hematopoiesis: Are leukemias a stem cell disorder or a reacquisition of stem cell characteristics?. Proc. Natl. Acad. Sci. USA.

[17] Maugeri-Saccà M., Vigneri P., De Maria R. (2011). Cancer Stem Cells and Chemosensitivity. Clin. Cancer Res..

[18] Arnold C.R., Mangesius J., Skvortsova I.-I., Ganswindt U. (2020). The Role of Cancer Stem Cells in Radiation Resistance. Front. Oncol..

[19] Jordan C.T., Guzman M.L., Noble M. (2006). Cancer Stem Cells. N. Engl. J. Med..

[20] Creighton C.J., Li X., Landis M., Dixon J.M., Neumeister V.M., Sjolund A., Rimm D.L., Wong H., Rodriguez A., Herschkowitz J.I. (2009). Residual breast cancers after conventional therapy display mesenchymal as well as tumor-initiating features. Proc. Natl. Acad. Sci. USA.

[21] Han J., Won M., Kim J.H., Jung E., Min K., Jangili P., Kim J.S. (2020). Cancer stem cell-targeted bio-imaging and chemotherapeutic perspective. Chem. Soc. Rev..

[22] Plaks V., Kong N., Werb Z. (2015). The Cancer Stem Cell Niche: How Essential Is the Niche in Regulating Stemness of Tumor Cells?. Cell Stem Cell.

[23] Talukdar S., Bhoopathi P., Emdad L., Das S., Sarkar D., Fisher P.B. (2019). Dormancy and cancer stem cells: An enigma for cancer therapeutic targeting. Adv. Cancer Res..

[24] Liang H., Deng L., Burnette B., Weichselbaum R.R., Fu Y.X. (2013). Radiation-induced tumor dormancy reflects an equilibrium between the proliferation and T lymphocyte-mediated death of malignant cells. Oncoimmunology.

[25] Vollmann-Zwerenz A., Leidgens V., Feliciello G., Klein C.A., Hau P. (2020). Tumor Cell Invasion in Glioblastoma. Int. J. Mol. Sci..

[26] Wang W., Xiong Y., Ding X., Wang L., Zhao Y., Fei Y., Zhu Y., Shen X., Tan C., Liang Z. (2019). Cathepsin L activated by mutant p53 and Egr-1 promotes ionizing radiation-induced EMT in human NSCLC. J. Exp. Clin. Cancer Res..

[27] Iser I.C., Pereira M.B., Lenz G., Wink M.R. (2017). The Epithelial-to-Mesenchymal Transition-Like Process in Glioblastoma: An Updated Systematic Review and In Silico Investigation. Med. Res. Rev..

[28] Nieto M.A., Huang R.Y., Jackson R.A., Thiery J.P. (2016). Emt: 2016. Cell.

[29] Loh C.-Y., Chai J., Tang T., Wong W., Sethi G., Shanmugam M., Chong P., Looi C. (2019). The E-Cadherin and N-Cadherin Switch in Epithelial-to-Mesenchymal Transition: Signaling, Therapeutic Implications, and Challenges. Cells.

[30] Mani S.A., Guo W., Liao M.J., Eaton E.N., Ayyanan A., Zhou A.Y., Brooks M., Reinhard F., Zhang C.C., Shipitsin M. (2008). The epithelial-mesenchymal transition generates cells with properties of stem cells. Cell.

[31] Zhang F., Zhang T., Teng Z.-h., Zhang R., Wang J.-B., Mei Q.-B. (2014). Sensitization to γ-irradiation-induced cell cycle arrest and apoptosis by the histone deacetylase inhibitor trichostatin A in non-small cell lung cancer (NSCLC) cells. Cancer Biol. Ther..

[32] Madhusudan S., Middleton M.R. (2005). The emerging role of DNA repair proteins as predictive, prognostic and therapeutic targets in cancer. Cancer Treat. Rev..

[33] Karagiannis T.C., El-Osta A. (2004). Double-strand breaks: Signaling pathways and repair mechanisms. Cell Mol. Life Sci..

[34] Sikora E., Czarnecka-Herok J., Bojko A., Sunderland P. (2022). Therapy-induced polyploidization and senescence: Coincidence or interconnection?. Semin. Cancer Biol..

[35] d’Adda di Fagagna F., Reaper P.M., Clay-Farrace L., Fiegler H., Carr P., Von Zglinicki T., Saretzki G., Carter N.P., Jackson S.P. (2003). A DNA damage checkpoint response in telomere-initiated senescence. Nature.

[36] Shen Z., Huhn S.C., Haffty B.G. (2014). Escaping death to quiescence: Avoiding mitotic catastrophe after DNA damage. Cell Cycle.

[37] Kodym E., Kodym R., Reis A.E., Habib A.A., Story M.D., Saha D. (2009). The small-molecule CDK inhibitor, SNS-032, enhances cellular radiosensitivity in quiescent and hypoxic non-small cell lung cancer cells. Lung Cancer.

[38] Ivanov A., Cragg M.S., Erenpreisa J., Emzinsh D., Lukman H., Illidge T.M. (2003). Endopolyploid cells produced after severe genotoxic damage have the potential to repair DNA double strand breaks. J. Cell Sci..

[39] Erenpreisa J., Wheatley D. (2005). Endopolyploidy in development and cancer; “survival of the fattest?”. Cell Biology Int..

[40] Mirzayans R., Andrais B., Murray D. (2018). Roles of Polyploid/Multinucleated Giant Cancer Cells in Metastasis and Disease Relapse Following Anticancer Treatment. Cancers.

[41] Illidge T. (2000). Polyploid giant cells provide a survival mechanism for p53 mutant cells after dna damage. Cell Biol. Int..

[42] Puig P.-E., Guilly M.-N., Bouchot A., Droin N., Cathelin D., Bouyer F., Favier L., Ghiringhelli F., Kroemer G., Solary E. (2008). Tumor cells can escape DNA-damaging cisplatin through DNA endoreduplication and reversible polyploidy. Cell Biol. Int..

[43] Li R., Wang H., Liang Q., Chen L., Ren J. (2022). Radiotherapy for glioblastoma: Clinical issues and nanotechnology strategies. Biomater. Sci..

[44] Li Q., Zong Y., Li K., Jie X., Hong J., Zhou X., Wu B., Li Z., Zhang S., Wu G. (2019). Involvement of endothelial CK2 in the radiation induced perivascular resistant niche (PVRN) and the induction of radioresistance for non-small cell lung cancer (NSCLC) cells. Biol. Res..

[45] Friedmann-Morvinski D. (2014). Glioblastoma heterogeneity and cancer cell plasticity. Crit. Rev. Oncog..

[46] Chen Z., Fillmore C.M., Hammerman P.S., Kim C.F., Wong K.K. (2014). Non-small-cell lung cancers: A heterogeneous set of diseases. Nat. Rev. Cancer.

[47] Yaes R.J. (1989). Tumor heterogeneity, tumor size, and radioresistance. Int. J. Radiat. Oncol. Biol. Phys..

[48] Lim Z.F., Ma P.C. (2019). Emerging insights of tumor heterogeneity and drug resistance mechanisms in lung cancer targeted therapy. J. Hematol. Oncol..

[49] Soubannier V., Stifani S. (2017). NF-kappaB Signalling in Glioblastoma. Biomedicines.

[50] Iwadate Y. (2016). Epithelial-mesenchymal transition in glioblastoma progression. Oncol. Lett..

[51] Dagogo-Jack I., Shaw A.T. (2017). Tumour heterogeneity and resistance to cancer therapies. Nat. Rev. Clin. Oncol..

[52] Chaffer C.L., Weinberg R.A., Marjanovic N.D. (2013). Cell Plasticity and Heterogeneity in Cancer. Clin. Chem..

[53] Nowell P.C. (1976). The Clonal Evolution of Tumor Cell Populations. Science.

[54] Lovly C.M., Salama A.K., Salgia R. (2016). Tumor Heterogeneity and Therapeutic Resistance. Am. Soc. Clin. Oncol. Educ. Book.

[55] Rich J.N. (2016). Cancer stem cells. Medicine.

[56] Bonnet D., Dick J.E. (1997). Human acute myeloid leukemia is organized as a hierarchy that originates from a primitive hematopoietic cell. Nat. Med..

[57] Jin D.-Y., Leung E.L.-H., Fiscus R.R., Tung J.W., Tin V.P.-C., Cheng L.C., Sihoe A.D.-L., Fink L.M., Ma Y., Wong M.P. (2010). Non-Small Cell Lung Cancer Cells Expressing CD44 Are Enriched for Stem Cell-Like Properties. PLoS ONE.

[58] Visvader J.E., Lindeman G.J. (2008). Cancer stem cells in solid tumours: Accumulating evidence and unresolved questions. Nat. Rev. Cancer.

[59] Alamgeer M., Peacock C.D., Matsui W., Ganju V., Watkins D.N. (2013). Cancer stem cells in lung cancer: Evidence and controversies. Respirology.

[60] Ghisolfi L., Keates A.C., Hu X., Lee D.K., Li C.J. (2012). Ionizing radiation induces stemness in cancer cells. PLoS ONE.

[61] Liu Y., Yang M., Luo J., Zhou H. (2020). Radiotherapy targeting cancer stem cells "awakens" them to induce tumour relapse and metastasis in oral cancer. Int. J. Oral Sci..

[62] Koury J., Zhong L., Hao J. (2017). Targeting Signaling Pathways in Cancer Stem Cells for Cancer Treatment. Stem Cells Int..

[63] Najafi M., Farhood B., Mortezaee K. (2018). Cancer stem cells (CSCs) in cancer progression and therapy. J. Cell. Physiol..

[64] Liu G., Yuan X., Zeng Z., Tunici P., Ng H., Abdulkadir I.R., Lu L., Irvin D., Black K.L., Yu J.S. (2006). Analysis of gene expression and chemoresistance of CD133+ cancer stem cells in glioblastoma. Mol. Cancer.

[65] Zou Y.M., Hu G.Y., Zhao X.Q., Lu T., Zhu F., Yu S.Y., Xiong H. (2014). Hypoxia-induced autophagy contributes to radioresistance via c-Jun-mediated Beclin1 expression in lung cancer cells. J. Huazhong Univ. Sci. Technol. Med. Sci..

[66] Li F., Zhou K., Gao L., Zhang B., Li W., Yan W., Song X., Yu H., Wang S., Yu N. (2016). Radiation induces the generation of cancer stem cells: A novel mechanism for cancer radioresistance. Oncol. Lett..

[67] Yu Z., Pestell T.G., Lisanti M.P., Pestell R.G. (2012). Cancer stem cells. Int. J. Biochem. Cell Biol..

[68] Tabu K., Kimura T., Sasai K., Wang L., Bizen N., Nishihara H., Taga T., Tanaka S. (2010). Analysis of an alternative human CD133 promoter reveals the implication of Ras/ERK pathway in tumor stem-like hallmarks. Mol. Cancer.

[69] Isobe T., Hisamori S., Hogan D.J., Zabala M., Hendrickson D.G., Dalerba P., Cai S., Scheeren F., Kuo A.H., Sikandar S.S. (2014). miR-142 regulates the tumorigenicity of human breast cancer stem cells through the canonical WNT signaling pathway. eLife.

[70] Shimono Y., Zabala M., Cho R.W., Lobo N., Dalerba P., Qian D., Diehn M., Liu H., Panula S.P., Chiao E. (2009). Downregulation of miRNA-200c links breast cancer stem cells with normal stem cells. Cell.

[71] Huntly B.J., Gilliland D.G. (2005). Leukaemia stem cells and the evolution of cancer-stem-cell research. Nat. Rev. Cancer.

[72] Fiala S. (1968). The cancer cell as a stem cell unable to differentiate. A theory of carcinogenesis. Neoplasma.

[73] Clarkson B., Fried J., Strife A., Sakai Y., Ota K., Ohkita T. (1970). Studies of cellular proliferation in human leukemia.III. Behavior of leukemic cells in three adults with acute leukemia given continuous infusions of3H-thymidine for 8 or 10 days. Cancer.

[74] Clarkson B.D., Dowling M.D., Gee T.S., Cunningham I.B., Burchenal J.H. (1975). Treatment of acute leukemia in adults. Cancer.

[75] Hamburger A.W., Salmon S.E. (1977). Primary Bioassay of Human Tumor Stem Cells. Science.

[76] Lapidot T., Sirard C., Vormoor J., Murdoch B., Hoang T., Caceres-Cortes J., Minden M., Paterson B., Caligiuri M.A., Dick J.E. (1994). A cell initiating human acute myeloid leukaemia after transplantation into SCID mice. Nature.

[77] Lessard J., Sauvageau G. (2003). Bmi-1 determines the proliferative capacity of normal and leukaemic stem cells. Nature.

[78] Al-Hajj M., Wicha M.S., Benito-Hernandez A., Morrison S.J., Clarke M.F. (2003). Prospective identification of tumorigenic breast cancer cells. Proc. Natl. Acad. Sci. USA.

[79] Ailles L.E., Weissman I.L. (2007). Cancer stem cells in solid tumors. Curr. Opin. Biotechnol..

[80] Singh S.K., Clarke I.D., Terasaki M., Bonn V.E., Hawkins C., Squire J., Dirks P.B. (2003). Identification of a cancer stem cell in human brain tumors. Cancer Res..

[81] Matsui W., Huff C.A., Wang Q., Malehorn M.T., Barber J., Tanhehco Y., Smith B.D., Civin C.I., Jones R.J. (2004). Characterization of clonogenic multiple myeloma cells. Blood.

[82] Clarke M.F., Dick J.E., Dirks P.B., Eaves C.J., Jamieson C.H., Jones D.L., Visvader J., Weissman I.L., Wahl G.M. (2006). Cancer stem cells--perspectives on current status and future directions: AACR Workshop on cancer stem cells. Cancer Res..

[83] Zhang W.C., Shyh-Chang N., Yang H., Rai A., Umashankar S., Ma S., Soh B.S., Sun L.L., Tai B.C., Nga M.E. (2012). Glycine decarboxylase activity drives non-small cell lung cancer tumor-initiating cells and tumorigenesis. Cell.

[84] Li C., Heidt D.G., Dalerba P., Burant C.F., Zhang L., Adsay V., Wicha M., Clarke M.F., Simeone D.M. (2007). Identification of pancreatic cancer stem cells. Cancer Res..

[85] Collins A.T., Berry P.A., Hyde C., Stower M.J., Maitland N.J. (2005). Prospective identification of tumorigenic prostate cancer stem cells. Cancer Res..

[86] Aguglia U., Gambarelli D., Farnarier G., Quattrone A. (1991). Different susceptibilities of the geniculate and extrageniculate visual pathways to human Creutzfeldt-Jakob disease (a combined neurophysiological-neuropathological study). Electroencephalogr. Clin. Neurophysiol..

[87] Terris B., Cavard C., Perret C. (2010). EpCAM, a new marker for cancer stem cells in hepatocellular carcinoma. J. Hepatol..

[88] Zou G.M. (2007). Cancer stem cells in leukemia, recent advances. J. Cell Physiol..

[89] Boiko A.D., Razorenova O.V., van de Rijn M., Swetter S.M., Johnson D.L., Ly D.P., Butler P.D., Yang G.P., Joshua B., Kaplan M.J. (2010). Human melanoma-initiating cells express neural crest nerve growth factor receptor CD271. Nature.

[90] Liang M.H., Robb-Nicholson C. (1987). Health status and utility measurement viewed from the right brain: Experience from the rheumatic diseases. J. Chronic. Dis..

[91] Organista-Nava J., Gomez-Gomez Y., Garibay-Cerdenares O.L., Leyva-Vazquez M.A., Illades-Aguiar B. (2019). Cervical cancer stem cell-associated genes: Prognostic implications in cervical cancer. Oncol. Lett..

[92] Curley M.D., Therrien V.A., Cummings C.L., Sergent P.A., Koulouris C.R., Friel A.M., Roberts D.J., Seiden M.V., Scadden D.T., Rueda B.R. (2009). CD133 expression defines a tumor initiating cell population in primary human ovarian cancer. Stem Cells.

[93] Takaishi S., Okumura T., Tu S., Wang S.S., Shibata W., Vigneshwaran R., Gordon S.A., Shimada Y., Wang T.C. (2009). Identification of gastric cancer stem cells using the cell surface marker CD44. Stem Cells.

[94] Eramo A., Lotti F., Sette G., Pilozzi E., Biffoni M., Di Virgilio A., Conticello C., Ruco L., Peschle C., De Maria R. (2007). Identification and expansion of the tumorigenic lung cancer stem cell population. Cell Death Differ..

[95] Dubrovska A. (2014). Report on the International Workshop ‘Cancer stem cells: The mechanisms of radioresistance and biomarker discovery’. Int. J. Radiat. Biol..

[96] Peitzsch C., Nathansen J., Schniewind S.I., Schwarz F., Dubrovska A. (2019). Cancer Stem Cells in Head and Neck Squamous Cell Carcinoma: Identification, Characterization and Clinical Implications. Cancers.

[97] Gilbert C.A., Ross A.H. (2009). Cancer stem cells: Cell culture, markers, and targets for new therapies. J. Cell Biochem..

[98] Vlashi E., Pajonk F. (2015). The metabolic state of cancer stem cells—A valid target for cancer therapy?. Free Radic. Biol. Med..

[99] Siegel R.L., Miller K.D., Jemal A. (2019). Cancer statistics, 2019. CA Cancer J. Clin..

[100] Prieto-Vila M., Takahashi R.U., Usuba W., Kohama I., Ochiya T. (2017). Drug Resistance Driven by Cancer Stem Cells and Their Niche. Int. J. Mol. Sci..

[101] Rycaj K., Tang D.G. (2014). Cancer stem cells and radioresistance. Int. J. Radiat. Biol..

[102] Eyler C.E., Rich J.N. (2008). Survival of the fittest: Cancer stem cells in therapeutic resistance and angiogenesis. J. Clin. Oncol..

[103] Borah A., Raveendran S., Rochani A., Maekawa T., Kumar D.S. (2015). Targeting self-renewal pathways in cancer stem cells: Clinical implications for cancer therapy. Oncogenesis.

[104] Zhang M., Atkinson R.L., Rosen J.M. (2010). Selective targeting of radiation-resistant tumor-initiating cells. Proc. Natl. Acad. Sci. USA.

[105] Talukdar S., Pradhan A.K., Bhoopathi P., Shen X.-N., August L.A., Windle J.J., Sarkar D., Furnari F.B., Cavenee W.K., Das S.K. (2018). MDA-9/Syntenin regulates protective autophagy in anoikis-resistant glioma stem cells. Proc. Natl. Acad. Sci. USA.

[106] Talukdar S., Emdad L., Das S.K., Sarkar D., Fisher P.B. (2016). Evolving Strategies for Therapeutically Targeting Cancer Stem Cells. Adv. Cancer Res..

[107] Lyakhovich A., Lleonart M.E. (2016). Bypassing Mechanisms of Mitochondria-Mediated Cancer Stem Cells Resistance to Chemo- and Radiotherapy. Oxidative Med. Cell. Longev..

[108] Kurth I., Hein L., Mabert K., Peitzsch C., Koi L., Cojoc M., Kunz-Schughart L., Baumann M., Dubrovska A. (2015). Cancer stem cell related markers of radioresistance in head and neck squamous cell carcinoma. Oncotarget.

[109] Chang L., Graham P., Hao J., Ni J., Deng J., Bucci J., Malouf D., Gillatt D., Li Y. (2016). Cancer stem cells and signaling pathways in radioresistance. Oncotarget.

[110] Cojoc M., Mabert K., Muders M.H., Dubrovska A. (2015). A role for cancer stem cells in therapy resistance: Cellular and molecular mechanisms. Semin. Cancer Biol..

[111] Shin M.K., Cheong J.H. (2019). Mitochondria-centric bioenergetic characteristics in cancer stem-like cells. Arch. Pharm. Res..

[112] Dayem A.A., Choi H.Y., Kim J.H., Cho S.G. (2010). Role of oxidative stress in stem, cancer, and cancer stem cells. Cancers.

[113] Dando I., Cordani M., Dalla Pozza E., Biondani G., Donadelli M., Palmieri M. (2015). Antioxidant Mechanisms and ROS-Related MicroRNAs in Cancer Stem Cells. Oxid. Med. Cell Longev..

[114] Diehn M., Cho R.W., Lobo N.A., Kalisky T., Dorie M.J., Kulp A.N., Qian D., Lam J.S., Ailles L.E., Wong M. (2009). Association of reactive oxygen species levels and radioresistance in cancer stem cells. Nature.

[115] Mondal S., Bhattacharya K., Mandal C. (2018). Nutritional stress reprograms dedifferention in glioblastoma multiforme driven by PTEN/Wnt/Hedgehog axis: A stochastic model of cancer stem cells. Cell Death Discov..

[116] Szotek P.P., Pieretti-Vanmarcke R., Masiakos P.T., Dinulescu D.M., Connolly D., Foster R., Dombkowski D., Preffer F., Maclaughlin D.T., Donahoe P.K. (2006). Ovarian cancer side population defines cells with stem cell-like characteristics and Mullerian Inhibiting Substance responsiveness. Proc. Natl. Acad. Sci. USA.

[117] Müller E., Ansorge M., Werner C., Pompe T. (2014). Mimicking the Hematopoietic Stem Cell Niche by Biomaterials. Bio-Inspired Materials for Biomedical Engineering.

[118] Bissell M.J., Labarge M.A. (2005). Context, tissue plasticity, and cancer: Are tumor stem cells also regulated by the microenvironment?. Cancer Cell.

[119] Li Z., Bao S., Wu Q., Wang H., Eyler C., Sathornsumetee S., Shi Q., Cao Y., Lathia J., McLendon R.E. (2009). Hypoxia-Inducible Factors Regulate Tumorigenic Capacity of Glioma Stem Cells. Cancer Cell.

[120] Korkaya H., Liu S., Wicha M.S. (2011). Regulation of cancer stem cells by cytokine networks: Attacking cancer’s inflammatory roots. Clin. Cancer Res..

[121] Fukumura D., Xu L., Chen Y., Gohongi T., Seed B., Jain R.K. (2001). Hypoxia and acidosis independently up-regulate vascular endothelial growth factor transcription in brain tumors in vivo. Cancer Res..

[122] Hjelmeland A.B., Wu Q., Heddleston J.M., Choudhary G.S., MacSwords J., Lathia J.D., McLendon R., Lindner D., Sloan A., Rich J.N. (2010). Acidic stress promotes a glioma stem cell phenotype. Cell Death Differ..

[123] Liu J., Xiao Z., Wong S.K., Tin V.P., Ho K.Y., Wang J., Sham M.H., Wong M.P. (2013). Lung cancer tumorigenicity and drug resistance are maintained through ALDH(hi)CD44(hi) tumor initiating cells. Oncotarget.

[124] Lee C.J., Li C., Simeone D.M. (2008). Human Pancreatic Cancer Stem Cells: Implications for How We Treat Pancreatic Cancer. Transl. Oncol..

[125] Vail D.M. (2013). Withrow and MacEwen’s Small Animal Clinical Oncology.

[126] Ho M.M., Ng A.V., Lam S., Hung J.Y. (2007). Side population in human lung cancer cell lines and tumors is enriched with stem-like cancer cells. Cancer Res..

[127] Kim W.T., Ryu C.J. (2017). Cancer stem cell surface markers on normal stem cells. BMB Rep..

[128] Tan D., Roth I., Wickremesekera A., Davis P., Kaye A., Mantamadiotis T., Stylli S., Tan S. (2019). Therapeutic Targeting of Cancer Stem Cells in Human Glioblastoma by Manipulating the Renin-Angiotensin System. Cells.

[129] Zhang X., Zhao W., Li Y. (2017). Stemness-related markers in cancer. Cancer Transl. Med..

[130] Klonisch T., Wiechec E., Hombach-Klonisch S., Ande S.R., Wesselborg S., Schulze-Osthoff K., Los M. (2008). Cancer stem cell markers in common cancers—Therapeutic implications. Trends Mol. Med..

[131] Reya T., Morrison S.J., Clarke M.F., Weissman I.L. (2001). Stem cells, cancer, and cancer stem cells. Nature.

[132] Shackleton M., Vaillant F., Simpson K.J., Stingl J., Smyth G.K., Asselin-Labat M.-L., Wu L., Lindeman G.J., Visvader J.E. (2006). Generation of a functional mammary gland from a single stem cell. Nature.

[133] Redmer T., Walz I., Klinger B., Khouja S., Welte Y., Schäfer R., Regenbrecht C. (2017). The role of the cancer stem cell marker CD271 in DNA damage response and drug resistance of melanoma cells. Oncogenesis.

[134] Lupia M., Angiolini F., Bertalot G., Freddi S., Sachsenmeier K.F., Chisci E., Kutryb-Zajac B., Confalonieri S., Smolenski R.T., Giovannoni R. (2018). CD73 Regulates Stemness and Epithelial-Mesenchymal Transition in Ovarian Cancer-Initiating Cells. Stem Cell Rep..

[135] Taddei A., Giampietro C., Conti A., Orsenigo F., Breviario F., Pirazzoli V., Potente M., Daly C., Dimmeler S., Dejana E. (2008). Endothelial adherens junctions control tight junctions by VE-cadherin-mediated upregulation of claudin-5. Nat. Cell Biol..

[136] Medema J.P. (2013). Cancer stem cells: The challenges ahead. Nat. Cell Biol..

[137] Begicevic R.-R., Falasca M. (2017). ABC Transporters in Cancer Stem Cells: Beyond Chemoresistance. Int. J. Mol. Sci..

[138] Padmanabhan R., Chen K.G., Gottesman M.M. (2014). Lost in Translation: Regulation of ABCG2 Expression in Human Embryonic Stem Cells. J. Stem Cell Res..

[139] Markovsky E., Vax E., Ben-Shushan D., Eldar-Boock A., Shukrun R., Yeini E., Barshack I., Caspi R., Harari-Steinberg O., Pode-Shakked N. (2017). Wilms Tumor NCAM-Expressing Cancer Stem Cells as Potential Therapeutic Target for Polymeric Nanomedicine. Mol. Cancer Ther..

[140] Pustovalova M., Blokhina T., Alhaddad L., Chigasova A., Chuprov-Netochin R., Veviorskiy A., Filkov G., Osipov A.N., Leonov S. (2022). CD44+ and CD133+ Non-Small Cell Lung Cancer Cells Exhibit DNA Damage Response Pathways and Dormant Polyploid Giant Cancer Cell Enrichment Relating to Their p53 Status. Int. J. Mol. Sci..

[141] Roy S.K., Shrivastava A., Srivastav S., Shankar S., Srivastava R.K. (2020). SATB2 is a novel biomarker and therapeutic target for cancer. J. Cell. Mol. Med..

[142] Bao B., Wang Z., Ali S., Kong D., Banerjee S., Ahmad A., Li Y., Azmi A.S., Miele L., Sarkar F.H. (2011). Over-expression of FoxM1 leads to epithelial-mesenchymal transition and cancer stem cell phenotype in pancreatic cancer cells. J. Cell. Biochem..

[143] Qi Y., Wei J., Zhang X. (2021). Requirement of transcription factor NME2 for the maintenance of the stemness of gastric cancer stem-like cells. Cell Death Dis..

[144] Zhang Q., Han Z., Zhu Y., Chen J., Li W. (2021). Role of hypoxia inducible factor-1 in cancer stem cells (Review). Mol. Med. Rep..

[145] Liu A., Yu X., Liu S. (2013). Pluripotency transcription factors and cancer stem cells: Small genes make a big difference. Chin. J. Cancer.

[146] Pandit H., Li Y., Li X., Zhang W., Li S., Martin R.C.G. (2018). Enrichment of cancer stem cells via beta-catenin contributing to the tumorigenesis of hepatocellular carcinoma. BMC Cancer.

[147] Wurdak H., Zhu S., Romero A., Lorger M., Watson J., Chiang C.Y., Zhang J., Natu V.S., Lairson L.L., Walker J.R. (2010). An RNAi screen identifies TRRAP as a regulator of brain tumor-initiating cell differentiation. Cell Stem Cell.

[148] Hao J., Zhang Y., Jing D., Li Y., Li J., Zhao Z. (2014). Role of Hippo Signaling in Cancer Stem Cells. J. Cell. Physiol..

[149] Safa A.R. (2016). Resistance to Cell Death and Its Modulation in Cancer Stem Cells. Crit. Rev. Oncog..

[150] Hong M., Tan H., Li S., Cheung F., Wang N., Nagamatsu T., Feng Y. (2016). Cancer Stem Cells: The Potential Targets of Chinese Medicines and Their Active Compounds. Int. J. Mol. Sci..

[151] Zakaria N., Mohd Yusoff N., Zakaria Z., Widera D., Yahaya B.H. (2018). Inhibition of NF-kappaB Signaling Reduces the Stemness Characteristics of Lung Cancer Stem Cells. Front. Oncol..

[152] Kelly P.N., Strasser A. (2011). The role of Bcl-2 and its pro-survival relatives in tumourigenesis and cancer therapy. Cell Death Differ..

[153] Visvader J.E., Lindeman G.J. (2012). Cancer stem cells: Current status and evolving complexities. Cell Stem Cell.

[154] Badve S., Nakshatri H. (2012). Breast-cancer stem cells-beyond semantics. Lancet Oncol..

[155] Liu S., Cong Y., Wang D., Sun Y., Deng L., Liu Y., Martin-Trevino R., Shang L., McDermott S.P., Landis M.D. (2014). Breast Cancer Stem Cells Transition between Epithelial and Mesenchymal States Reflective of their Normal Counterparts. Stem Cell Rep..

[156] Aghajani M., Mansoori B., Mohammadi A., Asadzadeh Z., Baradaran B. (2019). New emerging roles of CD133 in cancer stem cell: Signaling pathway and miRNA regulation. J. Cell. Physiol..

[157] Desai A., Webb B., Gerson S.L. (2014). CD133+ cells contribute to radioresistance via altered regulation of DNA repair genes in human lung cancer cells. Radiother. Oncol..

[158] Levina V., Marrangoni A., Wang T., Parikh S., Su Y., Herberman R., Lokshin A., Gorelik E. (2010). Elimination of Human Lung Cancer Stem Cells through Targeting of the Stem Cell Factor–c-kit Autocrine Signaling Loop. Cancer Res..

[159] Saya H. (2017). MS 26.01 Therapeutic Strategies Targeting Cancer Stem Cells. J. Thorac. Oncol..

[160] Tsubouchi K., Minami K., Hayashi N., Yokoyama Y., Mori S., Yamamoto H., Koizumi M. (2017). The CD44 standard isoform contributes to radioresistance of pancreatic cancer cells. J. Radiat. Res..

[161] Kaidi A., Williams A.C., Paraskeva C. (2007). Interaction between β-catenin and HIF-1 promotes cellular adaptation to hypoxia. Nat. Cell Biol..

[162] Moeller B.J., Dreher M.R., Rabbani Z.N., Schroeder T., Cao Y., Li C.Y., Dewhirst M.W. (2005). Pleiotropic effects of HIF-1 blockade on tumor radiosensitivity. Cancer Cell.

[163] Dimri G.P., Martinez J.L., Jacobs J.J., Keblusek P., Itahana K., Van Lohuizen M., Campisi J., Wazer D.E., Band V. (2002). The Bmi-1 oncogene induces telomerase activity and immortalizes human mammary epithelial cells. Cancer Res..

[164] Tao W., Zhang A., Zhai K., Huang Z., Huang H., Zhou W., Huang Q., Fang X., Prager B.C., Wang X. (2020). SATB2 drives glioblastoma growth by recruiting CBP to promote FOXM1 expression in glioma stem cells. EMBO Mol. Med..

[165] Gottesman M.M., Fojo T., Bates S.E. (2002). Multidrug resistance in cancer: Role of ATP-dependent transporters. Nat. Rev. Cancer.

[166] Copsel S., Garcia C., Diez F., Vermeulem M., Baldi A., Bianciotti L.G., Russel F.G., Shayo C., Davio C. (2011). Multidrug resistance protein 4 (MRP4/ABCC4) regulates cAMP cellular levels and controls human leukemia cell proliferation and differentiation. J. Biol. Chem..

[167] Glavinas H., Krajcsi P., Cserepes J., Sarkadi B. (2004). The role of ABC transporters in drug resistance, metabolism and toxicity. Curr. Drug Deliv..

[168] Bleau A.-M., Hambardzumyan D., Ozawa T., Fomchenko E.I., Huse J.T., Brennan C.W., Holland E.C. (2009). PTEN/PI3K/Akt Pathway Regulates the Side Population Phenotype and ABCG2 Activity in Glioma Tumor Stem-like Cells. Cell Stem Cell.

[169] Moitra K. (2015). Overcoming Multidrug Resistance in Cancer Stem Cells. BioMed Res. Int..

[170] Pustovalova M., Alhaddad L., Smetanina N., Chigasova A., Blokhina T., Chuprov-Netochin R., Osipov A.N., Leonov S. (2020). The p53-53BP1-Related Survival of A549 and H1299 Human Lung Cancer Cells after Multifractionated Radiotherapy Demonstrated Different Response to Additional Acute X-ray Exposure. Int. J. Mol. Sci..

[171] Castellan M., Guarnieri A., Fujimura A., Zanconato F., Battilana G., Panciera T., Sladitschek H.L., Contessotto P., Citron A., Grilli A. (2020). Single-cell analyses reveal YAP/TAZ as regulators of stemness and cell plasticity in glioblastoma. Nat. Cancer.

[172] Benham-Pyle B.W., Pruitt B.L., Nelson W.J. (2015). Cell adhesion. Mechanical strain induces E-cadherin-dependent Yap1 and beta-catenin activation to drive cell cycle entry. Science.

[173] Shreberk-Shaked M., Dassa B., Sinha S., Di Agostino S., Azuri I., Mukherjee S., Aylon Y., Blandino G., Ruppin E., Oren M. (2020). A Division of Labor between YAP and TAZ in Non-Small Cell Lung Cancer. Cancer Res..

[174] Overholtzer M., Zhang J., Smolen G.A., Muir B., Li W., Sgroi D.C., Deng C.X., Brugge J.S., Haber D.A. (2006). Transforming properties of YAP, a candidate oncogene on the chromosome 11q22 amplicon. Proc. Natl. Acad. Sci. USA.

[175] Neradil J., Veselska R. (2015). Nestin as a marker of cancer stem cells. Cancer Sci..

[176] O’Brien C.A., Kreso A., Jamieson C.H. (2010). Cancer stem cells and self-renewal. Clin. Cancer Res..

[177] Stahl S., Fung E., Adams C., Lengqvist J., Mork B., Stenerlow B., Lewensohn R., Lehtio J., Zubarev R., Viktorsson K. (2009). Proteomics and pathway analysis identifies JNK signaling as critical for high linear energy transfer radiation-induced apoptosis in non-small lung cancer cells. Mol. Cell Proteom..

[178] Yang L., Shi P., Zhao G., Xu J., Peng W., Zhang J., Zhang G., Wang X., Dong Z., Chen F. (2020). Targeting cancer stem cell pathways for cancer therapy. Signal Transduct. Target. Ther..

[179] Khan A.Q., Ahmed E.I., Elareer N.R., Junejo K., Steinhoff M., Uddin S. (2019). Role of miRNA-Regulated Cancer Stem Cells in the Pathogenesis of Human Malignancies. Cells.

[180] Correnti M., Booijink R., Di Maira G., Raggi C., Marra F. (2018). Stemness features in liver cancer. Hepatoma Res..

[181] Kim K.W., Kim J.Y., Qiao J., Clark R.A., Powers C.M., Correa H., Chung D.H. (2019). Dual-Targeting AKT2 and ERK in cancer stem-like cells in neuroblastoma. Oncotarget.

[182] Wang J., Wakeman T.P., Lathia J.D., Hjelmeland A.B., Wang X.F., White R.R., Rich J.N., Sullenger B.A. (2010). Notch promotes radioresistance of glioma stem cells. Stem Cells.

[183] Stewart D.J. (2014). Wnt signaling pathway in non-small cell lung cancer. J. Natl. Cancer Inst..

[184] Matsui W.H. (2016). Cancer stem cell signaling pathways. Medicine.

[185] Schreck K.C., Taylor P., Marchionni L., Gopalakrishnan V., Bar E.E., Gaiano N., Eberhart C.G. (2010). The Notch target Hes1 directly modulates Gli1 expression and Hedgehog signaling: A potential mechanism of therapeutic resistance. Clin. Cancer Res..

[186] Vlashi E., Pajonk F. (2015). Cancer stem cells, cancer cell plasticity and radiation therapy. Semin. Cancer Biol..

[187] Yang Y., Zhou H., Zhang G., Xue X. (2019). Targeting the canonical Wnt/beta-catenin pathway in cancer radioresistance: Updates on the molecular mechanisms. J. Cancer Res. Ther..

[188] Liu C., Wang R. (2019). The Roles of Hedgehog Signaling Pathway in Radioresistance of Cervical Cancer. Dose-Response.

[189] Kim R.K., Kaushik N., Suh Y., Yoo K.C., Cui Y.H., Kim M.J., Lee H.J., Kim I.G., Lee S.J. (2016). Radiation driven epithelial-mesenchymal transition is mediated by Notch signaling in breast cancer. Oncotarget.

[190] Kong D., Banerjee S., Ahmad A., Li Y., Wang Z., Sethi S., Sarkar F.H. (2010). Epithelial to mesenchymal transition is mechanistically linked with stem cell signatures in prostate cancer cells. PLoS ONE.

[191] Hassan K.A., Wang L., Korkaya H., Chen G., Maillard I., Beer D.G., Kalemkerian G.P., Wicha M.S. (2013). Notch pathway activity identifies cells with cancer stem cell-like properties and correlates with worse survival in lung adenocarcinoma. Clin. Cancer Res..

[192] Park J.H., Shin J.E., Park H.W. (2018). The Role of Hippo Pathway in Cancer Stem Cell Biology. Mol. Cells.

[193] Zhang Y., Wang Y., Zhou D., Wang K., Wang X., Wang X., Jiang Y., Zhao M., Yu R., Zhou X. (2021). Radiation-induced YAP activation confers glioma radioresistance via promoting FGF2 transcription and DNA damage repair. Oncogene.

[194] Yang K., Zhao Y., Du Y., Tang R. (2021). Evaluation of Hippo Pathway and CD133 in Radiation Resistance in Small-Cell Lung Cancer. J. Oncol..

[195] Krause M., Dubrovska A., Linge A., Baumann M. (2017). Cancer stem cells: Radioresistance, prediction of radiotherapy outcome and specific targets for combined treatments. Adv. Drug Deliv. Rev..

[196] Maugeri-Saccà M., Bartucci M., De Maria R. (2012). DNA Damage Repair Pathways in Cancer Stem Cells. Mol. Cancer Ther..

[197] Eriksson D., Stigbrand T. (2010). Radiation-induced cell death mechanisms. Tumor Biol..

[198] Babayan N., Vorobyeva N., Grigoryan B., Grekhova A., Pustovalova M., Rodneva S., Fedotov Y., Tsakanova G., Aroutiounian R., Osipov A. (2020). Low Repair Capacity of DNA Double-Strand Breaks Induced by Laser-Driven Ultrashort Electron Beams in Cancer Cells. Int. J. Mol. Sci..

[199] Mladenov E., Magin S., Soni A., Iliakis G. (2013). DNA Double-Strand Break Repair as Determinant of Cellular Radiosensitivity to Killing and Target in Radiation Therapy. Front. Oncol..

[200] Bushmanov A., Vorobyeva N., Molodtsova D., Osipov A.N. (2022). Utilization of DNA double-strand breaks for biodosimetry of ionizing radiation exposure. Environ. Adv..

[201] Biau J., Chautard E., Berthault N., de Koning L., Court F., Pereira B., Verrelle P., Dutreix M. (2019). Combining the DNA Repair Inhibitor Dbait With Radiotherapy for the Treatment of High Grade Glioma: Efficacy and Protein Biomarkers of Resistance in Preclinical Models. Front. Oncol..

[202] Wyman C., Kanaar R. (2006). DNA double-strand break repair: All’s well that ends well. Annu. Rev. Genet..

[203] Morgan M.A., Lawrence T.S. (2015). Molecular Pathways: Overcoming Radiation Resistance by Targeting DNA Damage Response Pathways. Clin. Cancer Res..

[204] Biau J., Chautard E., Verrelle P., Dutreix M. (2019). Altering DNA Repair to Improve Radiation Therapy: Specific and Multiple Pathway Targeting. Front. Oncol..

[205] Ulyanenko S., Pustovalova M., Koryakin S., Beketov E., Lychagin A., Ulyanenko L., Kaprin A., Grekhova A., Ozerova A.M., Ozerov I.V. (2019). Formation of gammaH2AX and pATM Foci in Human Mesenchymal Stem Cells Exposed to Low Dose-Rate Gamma-Radiation. Int. J. Mol. Sci..

[206] Britton S., Coates J., Jackson S.P. (2013). A new method for high-resolution imaging of Ku foci to decipher mechanisms of DNA double-strand break repair. J. Cell Biol..

[207] Park E.J., Chan D.W., Park J.H., Oettinger M.A., Kwon J. (2003). DNA-PK is activated by nucleosomes and phosphorylates H2AX within the nucleosomes in an acetylation-dependent manner. Nucleic Acids Res..

[208] Ma Y., Pannicke U., Schwarz K., Lieber M.R. (2002). Hairpin Opening and Overhang Processing by an Artemis/DNA-Dependent Protein Kinase Complex in Nonhomologous End Joining and V(D)J Recombination. Cell.

[209] Nick McElhinny S.A., Snowden C.M., McCarville J., Ramsden D.A. (2000). Ku Recruits the XRCC4-Ligase IV Complex to DNA Ends. Mol. Cell. Biol..

[210] Ahnesorg P., Smith P., Jackson S.P. (2006). XLF Interacts with the XRCC4-DNA Ligase IV Complex to Promote DNA Nonhomologous End-Joining. Cell.

[211] Belyaev I.Y. (2010). Radiation-induced DNA repair foci: Spatio-temporal aspects of formation, application for assessment of radiosensitivity and biological dosimetry. Mutat. Res./Rev. Mutat. Res..

[212] Tsvetkova A., Ozerov I.V., Pustovalova M., Grekhova A., Eremin P., Vorobyeva N., Eremin I., Pulin A., Zorin V., Kopnin P. (2017). γH2AX, 53BP1 and Rad51 protein foci changes in mesenchymal stem cells during prolonged X-ray irradiation. Oncotarget.

[213] Mao Z., Bozzella M., Seluanov A., Gorbunova V. (2014). DNA repair by nonhomologous end joining and homologous recombination during cell cycle in human cells. Cell Cycle.

[214] Kakarougkas A., Jeggo P.A. (2014). DNA DSB repair pathway choice: An orchestrated handover mechanism. Br. J. Radiol..

[215] Safa A.R., Saadatzadeh M.R., Cohen-Gadol A.A., Pollok K.E., Bijangi-Vishehsaraei K. (2015). Glioblastoma stem cells (GSCs) epigenetic plasticity and interconversion between differentiated non-GSCs and GSCs. Genes Dis..

[216] Vlashi E., Lagadec C., Vergnes L., Matsutani T., Masui K., Poulou M., Popescu R., Della Donna L., Evers P., Dekmezian C. (2011). Metabolic state of glioma stem cells and nontumorigenic cells. Proc. Natl. Acad. Sci. USA.

[217] Ogden A.T., Waziri A.E., Lochhead R.A., Fusco D., Lopez K., Ellis J.A., Kang J., Assanah M., McKhann G.M., Sisti M.B. (2008). Identification of A2b5+Cd133− Tumor-Initiating Cells in Adult Human Gliomas. Neurosurgery.

[218] Aum D.J., Kim D.H., Beaumont T.L., Leuthardt E.C., Dunn G.P., Kim A.H. (2014). Molecular and cellular heterogeneity: The hallmark of glioblastoma. Neurosurg. Focus.

[219] Nakano I. (2015). Stem cell signature in glioblastoma: Therapeutic development for a moving target. J. Neurosurg..

[220] Jackson E.L., Alvarez-Buylla A. (2008). Characterization of adult neural stem cells and their relation to brain tumors. Cells Tissues Organs.

[221] Zhu Y., Guignard F., Zhao D., Liu L., Burns D.K., Mason R.P., Messing A., Parada L.F. (2005). Early inactivation of p53 tumor suppressor gene cooperating with NF1 loss induces malignant astrocytoma. Cancer Cell.

[222] Kondo T., Setoguchi T., Taga T. (2004). Persistence of a small subpopulation of cancer stem-like cells in the C6 glioma cell line. Proc. Natl. Acad. Sci. USA.

[223] Goodell M.A., Brose K., Paradis G., Conner A.S., Mulligan R.C. (1996). Isolation and functional properties of murine hematopoietic stem cells that are replicating in vivo. J. Exp. Med..

[224] Lathia J.D., Mack S.C., Mulkearns-Hubert E.E., Valentim C.L., Rich J.N. (2015). Cancer stem cells in glioblastoma. Genes Dev..

[225] Bao S., Wu Q., McLendon R.E., Hao Y., Shi Q., Hjelmeland A.B., Dewhirst M.W., Bigner D.D., Rich J.N. (2006). Glioma stem cells promote radioresistance by preferential activation of the DNA damage response. Nature.

[226] Lim Y.C., Roberts T.L., Day B.W., Harding A., Kozlov S., Kijas A.W., Ensbey K.S., Walker D.G., Lavin M.F. (2012). A role for homologous recombination and abnormal cell-cycle progression in radioresistance of glioma-initiating cells. Mol. Cancer Ther..

[227] Son M.J., Woolard K., Nam D.H., Lee J., Fine H.A. (2009). SSEA-1 is an enrichment marker for tumor-initiating cells in human glioblastoma. Cell Stem Cell.

[228] Patru C., Romao L., Varlet P., Coulombel L., Raponi E., Cadusseau J., Renault-Mihara F., Thirant C., Leonard N., Berhneim A. (2010). CD133, CD15/SSEA-1, CD34 or side populations do not resume tumor-initiating properties of long-term cultured cancer stem cells from human malignant glio-neuronal tumors. BMC Cancer.

[229] Singh S.K., Hawkins C., Clarke I.D., Squire J.A., Bayani J., Hide T., Henkelman R.M., Cusimano M.D., Dirks P.B. (2004). Identification of human brain tumour initiating cells. Nature.

[230] Huang Z., Cheng L., Guryanova O.A., Wu Q., Bao S. (2010). Cancer stem cells in glioblastoma--molecular signaling and therapeutic targeting. Protein Cell.

[231] Mao X.G., Zhang X., Xue X.Y., Guo G., Wang P., Zhang W., Fei Z., Zhen H.N., You S.W., Yang H. (2009). Brain Tumor Stem-Like Cells Identified by Neural Stem Cell Marker CD15. Transl. Oncol..

[232] Jijiwa M., Demir H., Gupta S., Leung C., Joshi K., Orozco N., Huang T., Yildiz V.O., Shibahara I., de Jesus J.A. (2011). CD44v6 regulates growth of brain tumor stem cells partially through the AKT-mediated pathway. PLoS ONE.

[233] Bao S., Wu Q., Li Z., Sathornsumetee S., Wang H., McLendon R.E., Hjelmeland A.B., Rich J.N. (2008). Targeting cancer stem cells through L1CAM suppresses glioma growth. Cancer Res..

[234] Lathia J.D., Gallagher J., Heddleston J.M., Wang J., Eyler C.E., Macswords J., Wu Q., Vasanji A., McLendon R.E., Hjelmeland A.B. (2010). Integrin alpha 6 regulates glioblastoma stem cells. Cell Stem Cell.

[235] Yoo K.C., Kang J.H., Choi M.Y., Suh Y., Zhao Y., Kim M.J., Chang J.H., Shim J.K., Yoon S.J., Kang S.G. (2022). Soluble ICAM-1 a Pivotal Communicator between Tumors and Macrophages, Promotes Mesenchymal Shift of Glioblastoma. Adv. Sci..

[236] Reifenberger G., Szymas J., Wechsler W. (1987). Differential expression of glial- and neuronal-associated antigens in human tumors of the central and peripheral nervous system. Acta Neuropathol..

[237] Hagiwara H., Aotsuka Y., Yamamoto Y., Miyahara J., Mitoh Y. (2001). Determination of the antigen/epitope that is recognized by human monoclonal antibody CLN-IgG. Hum. Antibodies.

[238] Babic B., Matthias Corvinus F., Hadjijusufovic E., Tagkalos E., Lang H., Grimminger P. (2018). Ps01.162: Is There a Difference in Survival between Younger and Older Gastric Cancer (Including Aeg Ii and Aeg Iii) Patients after Gastrectomy?. Dis. Esophagus.

[239] Hugwil A.V. (2013). The meaning of the anti-cancer antibody CLN-IgG (Pritumumab) generated by human×human hybridoma technology against the cyto-skeletal protein, vimentin, in the course of the treatment of malignancy. Med. Hypotheses.

[240] Clement V., Sanchez P., de Tribolet N., Radovanovic I., Ruiz i Altaba A. (2007). HEDGEHOG-GLI1 Signaling Regulates Human Glioma Growth, Cancer Stem Cell Self-Renewal, and Tumorigenicity. Curr. Biol..

[241] Zhu J., Wang H., Sun Q., Ji X., Zhu L., Cong Z., Zhou Y., Liu H., Zhou M. (2013). Nrf2 is required to maintain the self-renewal of glioma stem cells. BMC Cancer.

[242] Zhu J., Wang H., Fan Y., Lin Y., Zhang L., Ji X., Zhou M. (2014). Targeting the NF-E2-related factor 2 pathway: A novel strategy for glioblastoma (review). Oncol. Rep..

[243] Leelatian N., Ihrie R.A. (2016). Head of the Class: OLIG2 and Glioblastoma Phenotype. Cancer Cell.

[244] Liu M., Dai B., Kang S.H., Ban K., Huang F.J., Lang F.F., Aldape K.D., Xie T.X., Pelloski C.E., Xie K. (2006). FoxM1B is overexpressed in human glioblastomas and critically regulates the tumorigenicity of glioma cells. Cancer Res..

[245] Li X.T., Li J.C., Feng M., Zhou Y.X., Du Z.W. (2019). Novel lncRNA-ZNF281 regulates cell growth, stemness and invasion of glioma stem-like U251s cells. Neoplasma.

[246] Suva M.L., Rheinbay E., Gillespie S.M., Patel A.P., Wakimoto H., Rabkin S.D., Riggi N., Chi A.S., Cahill D.P., Nahed B.V. (2014). Reconstructing and reprogramming the tumor-propagating potential of glioblastoma stem-like cells. Cell.

[247] Qiang L., Wu T., Zhang H.W., Lu N., Hu R., Wang Y.J., Zhao L., Chen F.H., Wang X.T., You Q.D. (2012). HIF-1alpha is critical for hypoxia-mediated maintenance of glioblastoma stem cells by activating Notch signaling pathway. Cell Death Differ..

[248] Dahlrot R.H., Hermansen S.K., Hansen S., Kristensen B.W. (2013). What is the clinical value of cancer stem cell markers in gliomas?. Int. J. Clin. Exp. Pathol..

[249] Banelli B., Forlani A., Allemanni G., Morabito A., Pistillo M.P., Romani M. (2017). MicroRNA in Glioblastoma: An Overview. Int. J. Genom..

[250] Charles N.A., Holland E.C. (2010). TRRAP and the maintenance of stemness in gliomas. Cell Stem Cell.

[251] Spehalski E.I., Peters C., Camphausen K.A., Tofilon P. (2017). Distinctions Between the Metabolic Changes in Glioblastoma Cells and Glioma Stem-like Cells Following Irradiation. Int. J. Radiat. Oncol. Biol. Phys..

[252] Godoy P.R.D.V., Mello S.S., Magalhães D.A.R., Donaires F.S., Nicolucci P., Donadi E.A., Passos G.A., Sakamoto-Hojo E.T. (2013). Ionizing radiation-induced gene expression changes in TP53 proficient and deficient glioblastoma cell lines. Mutat. Res./Genet. Toxicol. Environ. Mutagen..

[253] Camphausen K., Purow B., Sproull M., Scott T., Ozawa T., Deen D.F., Tofilon P.J. (2005). Orthotopic Growth of Human Glioma Cells Quantitatively and Qualitatively Influences Radiation-Induced Changes in Gene Expression. Cancer Res..

[254] Tsai M.-H., Cook J.A., Chandramouli G.V.R., DeGraff W., Yan H., Zhao S., Coleman C.N., Mitchell J.B., Chuang E.Y. (2007). Gene Expression Profiling of Breast, Prostate, and Glioma Cells following Single versus Fractionated Doses of Radiation. Cancer Res..

[255] Zhang L., Cheng F., Wei Y., Zhang L., Guo D., Wang B., Li W. (2018). Inhibition of TAZ contributes radiation-induced senescence and growth arrest in glioma cells. Oncogene.

[256] Behrooz A.B., Syahir A. (2021). Could We Address the Interplay Between CD133, Wnt/beta-Catenin, and TERT Signaling Pathways as a Potential Target for Glioblastoma Therapy?. Front. Oncol..

[257] Olive P.L. (1999). DNA damage and repair in individual cells: Applications of the comet assay in radiobiology. Int. J. Radiat. Biol..

[258] Legler J.M., Ries L.A., Smith M.A., Warren J.L., Heineman E.F., Kaplan R.S., Linet M.S. (1999). Cancer surveillance series [corrected]: Brain and other central nervous system cancers: Recent trends in incidence and mortality. J. Natl. Cancer Inst..

[259] Beier D., Hau P., Proescholdt M., Lohmeier A., Wischhusen J., Oefner P.J., Aigner L., Brawanski A., Bogdahn U., Beier C.P. (2007). CD133(+) and CD133(-) glioblastoma-derived cancer stem cells show differential growth characteristics and molecular profiles. Cancer Res..

[260] Campos B., Zeng L., Daotrong P.H., Eckstein V., Unterberg A., Mairbaurl H., Herold-Mende C. (2011). Expression and regulation of AC133 and CD133 in glioblastoma. Glia.

[261] Gambelli F., Sasdelli F., Manini I., Gambarana C., Oliveri G., Miracco C., Sorrentino V. (2012). Identification of cancer stem cells from human glioblastomas: Growth and differentiation capabilities and CD133/prominin-1 expression. Cell Biol. Int..

[262] Brown D.V., Filiz G., Daniel P.M., Hollande F., Dworkin S., Amiridis S., Kountouri N., Ng W., Morokoff A.P., Mantamadiotis T. (2017). Expression of CD133 and CD44 in glioblastoma stem cells correlates with cell proliferation, phenotype stability and intra-tumor heterogeneity. PLoS ONE.

[263] Kowalski-Chauvel A., Modesto A., Gouaze-Andersson V., Baricault L., Gilhodes J., Delmas C., Lemarie A., Toulas C., Cohen-Jonathan-Moyal E., Seva C. (2018). Alpha-6 integrin promotes radioresistance of glioblastoma by modulating DNA damage response and the transcription factor Zeb1. Cell Death Dis..

[264] Halliday J., Helmy K., Pattwell S.S., Pitter K.L., LaPlant Q., Ozawa T., Holland E.C. (2014). In vivo radiation response of proneural glioma characterized by protective p53 transcriptional program and proneural-mesenchymal shift. Proc. Natl. Acad. Sci. USA.

[265] Trog D., Moenkemann H., Haertel N., Schuller H., Golubnitschaja O. (2005). Expression of ABC-1 transporter is elevated in human glioma cells under irradiation and temozolomide treatment. Amino Acids.

[266] Ray S.K. (2016). The Transcription Regulator Kruppel-Like Factor 4 and Its Dual Roles of Oncogene in Glioblastoma and Tumor Suppressor in Neuroblastoma. Immunopathol. Dis Ther..

[267] Hayes J.D., Dinkova-Kostova A.T. (2014). The Nrf2 regulatory network provides an interface between redox and intermediary metabolism. Trends Biochem. Sci..

[268] Dreger H., Westphal K., Weller A., Baumann G., Stangl V., Meiners S., Stangl K. (2009). Nrf2-dependent upregulation of antioxidative enzymes: A novel pathway for proteasome inhibitor-mediated cardioprotection. Cardiovasc. Res..

[269] Singh A., Bodas M., Wakabayashi N., Bunz F., Biswal S. (2010). Gain of Nrf2 function in non-small-cell lung cancer cells confers radioresistance. Antioxid. Redox. Signal..

[270] Suzuki T., Yamamoto M. (2015). Molecular basis of the Keap1-Nrf2 system. Free Radic. Biol. Med..

[271] Shibata T., Ohta T., Tong K.I., Kokubu A., Odogawa R., Tsuta K., Asamura H., Yamamoto M., Hirohashi S. (2008). Cancer related mutations in NRF2 impair its recognition by Keap1-Cul3 E3 ligase and promote malignancy. Proc. Natl. Acad. Sci. USA.

[272] Facchino S., Abdouh M., Chatoo W., Bernier G. (2010). BMI1 confers radioresistance to normal and cancerous neural stem cells through recruitment of the DNA damage response machinery. J. Neurosci..

[273] Mukherjee B., McEllin B., Camacho C.V., Tomimatsu N., Sirasanagandala S., Nannepaga S., Hatanpaa K.J., Mickey B., Madden C., Maher E. (2009). EGFRvIII and DNA double-strand break repair: A molecular mechanism for radioresistance in glioblastoma. Cancer Res..

[274] Majc B., Sever T., Zaric M., Breznik B., Turk B., Lah T.T. (2020). Epithelial-to-mesenchymal transition as the driver of changing carcinoma and glioblastoma microenvironment. Biochim. Biophys. Acta Mol. Cell Res..

[275] Hovinga K.E., Stalpers L.J., van Bree C., Donker M., Verhoeff J.J., Rodermond H.M., Bosch D.A., van Furth W.R. (2005). Radiation-enhanced vascular endothelial growth factor (VEGF) secretion in glioblastoma multiforme cell lines--a clue to radioresistance?. J. Neurooncol..

[276] Lee C.G., Heijn M., di Tomaso E., Griffon-Etienne G., Ancukiewicz M., Koike C., Park K.R., Ferrara N., Jain R.K., Suit H.D. (2000). Anti-Vascular endothelial growth factor treatment augments tumor radiation response under normoxic or hypoxic conditions. Cancer Res..

[277] Maachani U.B., Shankavaram U., Kramp T., Tofilon P.J., Camphausen K., Tandle A.T. (2016). FOXM1 and STAT3 interaction confers radioresistance in glioblastoma cells. Oncotarget.

[278] Povlsen L.K., Beli P., Wagner S.A., Poulsen S.L., Sylvestersen K.B., Poulsen J.W., Nielsen M.L., Bekker-Jensen S., Mailand N., Choudhary C. (2012). Systems-wide analysis of ubiquitylation dynamics reveals a key role for PAF15 ubiquitylation in DNA-damage bypass. Nat. Cell Biol..

[279] Lee H.C., Kim D.W., Jung K.Y., Park I.C., Park M.J., Kim M.S., Woo S.H., Rhee C.H., Yoo H., Lee S.H. (2004). Increased expression of antioxidant enzymes in radioresistant variant from U251 human glioblastoma cell line. Int. J. Mol. Med..

[280] Flor S., Oliva C.R., Ali M.Y., Coleman K.L., Greenlee J.D., Jones K.A., Monga V., Griguer C.E. (2021). Catalase Overexpression Drives an Aggressive Phenotype in Glioblastoma. Antioxidants.

[281] Pang L.Y., Hurst E.A., Argyle D.J. (2016). Cyclooxygenase-2: A Role in Cancer Stem Cell Survival and Repopulation of Cancer Cells during Therapy. Stem Cells Int..

[282] Ma H.I., Chiou S.H., Hueng D.Y., Tai L.K., Huang P.I., Kao C.L., Chen Y.W., Sytwu H.K. (2011). Celecoxib and radioresistant glioblastoma-derived CD133+ cells: Improvement in radiotherapeutic effects. Laboratory investigation. J. Neurosurg..

[283] Wang W., Long L., Wang L., Tan C., Fei X., Chen L., Huang Q., Liang Z. (2016). Knockdown of Cathepsin L promotes radiosensitivity of glioma stem cells both in vivo and in vitro. Cancer Lett..

[284] Wang J., Yang T., Xu G., Liu H., Ren C., Xie W., Wang M. (2016). Cyclin-Dependent Kinase 2 Promotes Tumor Proliferation and Induces Radio Resistance in Glioblastoma. Transl. Oncol..

[285] Marampon F., Megiorni F., Camero S., Crescioli C., McDowell H.P., Sferra R., Vetuschi A., Pompili S., Ventura L., De Felice F. (2017). HDAC4 and HDAC6 sustain DNA double strand break repair and stem-like phenotype by promoting radioresistance in glioblastoma cells. Cancer Lett..

[286] Deng X., Ma L., Wu M., Zhang G., Jin C., Guo Y., Liu R. (2013). miR-124 radiosensitizes human glioma cells by targeting CDK4. J. Neurooncol..

[287] Moskwa P., Zinn P.O., Choi Y.E., Shukla S.A., Fendler W., Chen C.C., Lu J., Golub T.R., Hjelmeland A., Chowdhury D. (2014). A functional screen identifies miRs that induce radioresistance in glioblastomas. Mol. Cancer Res..

[288] Alkhaibary A., Alassiri A.H., AlSufiani F., Alharbi M.A. (2019). Ki-67 labeling index in glioblastoma; does it really matter?. Hematol./Oncol. Stem Cell Ther..

[289] Mastronardi L., Guiducci A., Puzzilli F., Ruggeri A. (1999). Relationship between Ki-67 labeling index and survival in high-grade glioma patients treated after surgery with tamoxifen. J. Neurosurg. Sci..

[290] Tamura K., Aoyagi M., Ando N., Ogishima T., Wakimoto H., Yamamoto M., Ohno K. (2013). Expansion of CD133-positive glioma cells in recurrent de novo glioblastomas after radiotherapy and chemotherapy. J. Neurosurg..

[291] Zottel A., Jovcevska I., Samec N., Komel R. (2021). Cytoskeletal proteins as glioblastoma biomarkers and targets for therapy: A systematic review. Crit. Rev. Oncol. Hematol..

[292] Nguemgo Kouam P., Rezniczek G.A., Kochanneck A., Priesch-Grzeszkowiak B., Hero T., Adamietz I.A., Buhler H. (2018). Robo1 and vimentin regulate radiation-induced motility of human glioblastoma cells. PLoS ONE.

[293] Chakravarti A., Zhai G.G., Zhang M., Malhotra R., Latham D.E., Delaney M.A., Robe P., Nestler U., Song Q., Loeffler J. (2004). Survivin enhances radiation resistance in primary human glioblastoma cells via caspase-independent mechanisms. Oncogene.

[294] Yu H., Zhang S., Ibrahim A.N., Wang J., Deng Z., Wang M. (2019). RCC2 promotes proliferation and radio-resistance in glioblastoma via activating transcription of DNMT1. Biochem. Biophys. Res. Commun..

[295] Han X., Xue X., Zhou H., Zhang G. (2017). A molecular view of the radioresistance of gliomas. Oncotarget.

[296] Marampon F., Gravina G.L., Zani B.M., Popov V.M., Fratticci A., Cerasani M., Di Genova D., Mancini M., Ciccarelli C., Ficorella C. (2014). Hypoxia sustains glioblastoma radioresistance through ERKs/DNA-PKcs/HIF-1alpha functional interplay. Int. J. Oncol..

[297] Marwick C. (1991). Pharmaceutical industry commission, government agencies seek to expedite new addiction therapy. JAMA.

[298] Kim S.H., Ezhilarasan R., Phillips E., Gallego-Perez D., Sparks A., Taylor D., Ladner K., Furuta T., Sabit H., Chhipa R. (2016). Serine/Threonine Kinase MLK4 Determines Mesenchymal Identity in Glioma Stem Cells in an NF-kappaB-dependent Manner. Cancer Cell.

[299] Bar E.E., Chaudhry A., Lin A., Fan X., Schreck K., Matsui W., Piccirillo S., Vescovi A.L., DiMeco F., Olivi A. (2007). Cyclopamine-mediated hedgehog pathway inhibition depletes stem-like cancer cells in glioblastoma. Stem Cells.

[300] Zhang N., Wei P., Gong A., Chiu W.T., Lee H.T., Colman H., Huang H., Xue J., Liu M., Wang Y. (2011). FoxM1 promotes beta-catenin nuclear localization and controls Wnt target-gene expression and glioma tumorigenesis. Cancer Cell.

[301] Burris H.A. (2013). Overcoming acquired resistance to anticancer therapy: Focus on the PI3K/AKT/mTOR pathway. Cancer Chemother. Pharm..

[302] Ashton C.H., Rawlins M.D., Tyrer S.P. (1991). Buspirone in benzodiazepine withdrawal. Br. J. Psychiatry.

[303] Cao Y., Lathia J.D., Eyler C.E., Wu Q., Li Z., Wang H., McLendon R.E., Hjelmeland A.B., Rich J.N. (2010). Erythropoietin Receptor Signaling Through STAT3 Is Required for Glioma Stem Cell Maintenance. Genes Cancer.

[304] Ali M.Y., Oliva C.R., Noman A.S.M., Allen B.G., Goswami P.C., Zakharia Y., Monga V., Spitz D.R., Buatti J.M., Griguer C.E. (2020). Radioresistance in Glioblastoma and the Development of Radiosensitizers. Cancers.

[305] Farooqi A.A., Siddik Z.H. (2015). Platelet-derived growth factor (PDGF) signalling in cancer: Rapidly emerging signalling landscape. Cell Biochem. Funct..

[306] Godlewski J., Nowicki M.O., Bronisz A., Williams S., Otsuki A., Nuovo G., Raychaudhury A., Newton H.B., Chiocca E.A., Lawler S. (2008). Targeting of the Bmi-1 oncogene/stem cell renewal factor by microRNA-128 inhibits glioma proliferation and self-renewal. Cancer Res..

[307] Koul D. (2008). PTEN signaling pathways in glioblastoma. Cancer Biol. Ther..

[308] Morgenroth A., Vogg A.T., Ermert K., Zlatopolskiy B., Mottaghy F.M. (2014). Hedgehog signaling sensitizes glioma stem cells to endogenous nano-irradiation. Oncotarget.

[309] Han N., Hu G., Shi L., Long G., Yang L., Xi Q., Guo Q., Wang J., Dong Z., Zhang M. (2017). Notch1 ablation radiosensitizes glioblastoma cells. Oncotarget.

[310] Bhat K.P.L., Balasubramaniyan V., Vaillant B., Ezhilarasan R., Hummelink K., Hollingsworth F., Wani K., Heathcock L., James J.D., Goodman L.D. (2013). Mesenchymal differentiation mediated by NF-kappaB promotes radiation resistance in glioblastoma. Cancer Cell.

[311] Xu R.X., Liu R.Y., Wu C.M., Zhao Y.S., Li Y., Yao Y.Q., Xu Y.H. (2015). DNA damage-induced NF-kappaB activation in human glioblastoma cells promotes miR-181b expression and cell proliferation. Cell Physiol. Biochem..

[312] Huang T.T., Wuerzberger-Davis S.M., Seufzer B.J., Shumway S.D., Kurama T., Boothman D.A., Miyamoto S. (2000). NF-kappaB activation by camptothecin. A linkage between nuclear DNA damage and cytoplasmic signaling events. J. Biol. Chem..

[313] McCool K.W., Miyamoto S. (2012). DNA damage-dependent NF-kappaB activation: NEMO turns nuclear signaling inside out. Immunol. Rev..

[314] Volcic M., Karl S., Baumann B., Salles D., Daniel P., Fulda S., Wiesmuller L. (2012). NF-kappaB regulates DNA double-strand break repair in conjunction with BRCA1-CtIP complexes. Nucleic Acids Res..

[315] Wu K., Jiang S.W., Thangaraju M., Wu G., Couch F.J. (2000). Induction of the BRCA2 promoter by nuclear factor-kappa B. J. Biol. Chem..

[316] Miyamoto S. (2011). Nuclear initiated NF-kappaB signaling: NEMO and ATM take center stage. Cell Res..

[317] Wu J.K., Ye Z., Darras B.T. (1993). Frequency of p53 tumor suppressor gene mutations in human primary brain tumors. Neurosurgery.

[318] Chen P., Iavarone A., Fick J., Edwards M., Prados M., Israel M.A. (1995). Constitutional p53 mutations associated with brain tumors in young adults. Cancer Genet. Cytogenet..

[319] Shu H.K., Kim M.M., Chen P., Furman F., Julin C.M., Israel M.A. (1998). The intrinsic radioresistance of glioblastoma-derived cell lines is associated with a failure of p53 to induce p21(BAX) expression. Proc. Natl. Acad. Sci. USA.

[320] Jiang Z., Pore N., Cerniglia G.J., Mick R., Georgescu M.M., Bernhard E.J., Hahn S.M., Gupta A.K., Maity A. (2007). Phosphatase and tensin homologue deficiency in glioblastoma confers resistance to radiation and temozolomide that is reversed by the protease inhibitor nelfinavir. Cancer Res..

[321] Li L., Liu F., Salmonsen R.A., Turner T.K., Litofsky N.S., Di Cristofano A., Pandolfi P.P., Jones S.N., Recht L.D., Ross A.H. (2002). PTEN in neural precursor cells: Regulation of migration, apoptosis, and proliferation. Mol. Cell Neurosci..

[322] Lomonaco S.L., Finniss S., Xiang C., Decarvalho A., Umansky F., Kalkanis S.N., Mikkelsen T., Brodie C. (2009). The induction of autophagy by gamma-radiation contributes to the radioresistance of glioma stem cells. Int. J. Cancer.

[323] Zhuang W., Qin Z., Liang Z. (2009). The role of autophagy in sensitizing malignant glioma cells to radiation therapy. Acta Biochim. Biophys. Sin..

[324] Gomez-Casal R., Bhattacharya C., Ganesh N., Bailey L., Basse P., Gibson M., Epperly M., Levina V. (2013). Non-small cell lung cancer cells survived ionizing radiation treatment display cancer stem cell and epithelial-mesenchymal transition phenotypes. Mol. Cancer.

[325] Shien K., Toyooka S., Ichimura K., Soh J., Furukawa M., Maki Y., Muraoka T., Tanaka N., Ueno T., Asano H. (2012). Prognostic impact of cancer stem cellTransporters in Cancer non-small cell lung cancer patients treated with induction chemoradiotherapy. Lung Cancer.

[326] Zakaria N., Yusoff N.M., Zakaria Z., Lim M.N., Baharuddin P.J., Fakiruddin K.S., Yahaya B. (2015). Human non-small cell lung cancer expresses putative cancer stem cell markers and exhibits the transcriptomic profile of multipotent cells. BMC Cancer.

[327] Lee H.J., Choe G., Jheon S., Sung S.W., Lee C.T., Chung J.H. (2010). CD24, a novel cancer biomarker, predicting disease-free survival of non-small cell lung carcinomas: A retrospective study of prognostic factor analysis from the viewpoint of forthcoming (seventh) new TNM classification. J. Thorac. Oncol..

[328] Karimi-Busheri F., Rasouli-Nia A., Zadorozhny V., Fakhrai H. (2013). CD24+/CD38- as new prognostic marker for non-small cell lung cancer. Multidiscip. Respir. Med..

[329] Summer R., Kotton D.N., Sun X., Ma B., Fitzsimmons K., Fine A. (2003). Side population cells and Bcrp1 expression in lung. Am. J. Physiol. Lung Cell Mol. Physiol..

[330] Editors P.O. (2020). Retraction: Identification and Characterization of Cells with Cancer Stem Cell Properties in Human Primary Lung Cancer Cell Lines. PLoS ONE.

[331] Jiang F., Qiu Q., Khanna A., Todd N.W., Deepak J., Xing L., Wang H., Liu Z., Su Y., Stass S.A. (2009). Aldehyde dehydrogenase 1 is a tumor stem cell-associated marker in lung cancer. Mol. Cancer Res..

[332] Liang D., Shi Y. (2012). Aldehyde dehydrogenase-1 is a specific marker for stem cells in human lung adenocarcinoma. Med. Oncol..

[333] Kiziltunc Ozmen H., Simsek M. (2020). Serum IL-23, E-selectin and sICAM levels in non-small cell lung cancer patients before and after radiotherapy. J. Int. Med. Res..

[334] Li F., Zeng H., Ying K. (2011). The combination of stem cell markers CD133 and ABCG2 predicts relapse in stage I non-small cell lung carcinomas. Med. Oncol..

[335] Kwa H.B., Michalides R.J.A.M., Dijkman J.H., Mooi W.J. (1996). The prognostic value of NCAM, p53 and cyclin D1 in resected non-small cell lung cancer. Lung Cancer.

[336] Zhang X.-Y., Dong Q.-G., Huang J.-S., Huang A.-M., Shi C.-L., Jin B., Sha H.-F., Feng J.-X., Geng Q., Zhou J. (2010). The expression of stem cell-related indicators as a prognostic factor in human lung adenocarcinoma. J. Surg. Oncol..

[337] Shao W., Chen H., He J. (2015). The role of SOX-2 on the survival of patients with non-small cell lung cancer. J. Thorac. Dis..

[338] Fadous-Khalife M.C., Aloulou N., Jalbout M., Hadchity J., Aftimos G., Paris F., Hadchity E. (2016). Kruppel-like factor 4: A new potential biomarker of lung cancer. Mol. Clin. Oncol..

[339] Chanvorachote P., Sriratanasak N., Nonpanya N. (2020). C-myc Contributes to Malignancy of Lung Cancer: A Potential Anticancer Drug Target. Anticancer Res..

[340] Ma Y.N., Zhang H.Y., Fei L.R., Zhang M.Y., Wang C.C., Luo Y., Han Y.C. (2018). SATB2 suppresses non-small cell lung cancer invasiveness by G9a. Clin. Exp. Med..

[341] Hsiao Y.J., Chang W.H., Chen H.Y., Hsu Y.C., Chiu S.C., Chiang C.C., Chang G.C., Chen Y.J., Wang C.Y., Chen Y.M. (2020). MITF functions as a tumor suppressor in non-small cell lung cancer beyond the canonically oncogenic role. Aging.

[342] Harada D., Takigawa N., Kiura K. (2014). The Role of STAT3 in Non-Small Cell Lung Cancer. Cancers.

[343] Wang G., Xiao L., Wang F., Yang J., Yang L., Zhao Y., Jin W. (2019). Hypoxia inducible factor-1alpha/B-cell lymphoma 2 signaling impacts radiosensitivity of H1299 non-small cell lung cancer cells in a normoxic environment. Radiat. Environ. Biophys..

[344] Lu J., Zhan Y., Feng J., Luo J., Fan S. (2018). MicroRNAs associated with therapy of non-small cell lung cancer. Int. J. Biol. Sci..

[345] Sone K., Maeno K., Masaki A., Kunii E., Takakuwa O., Kagawa Y., Takeuchi A., Fukuda S., Uemura T., Fukumitsu K. (2020). Nestin Expression Affects Resistance to Chemotherapy and Clinical Outcome in Small Cell Lung Cancer. Front. Oncol..

[346] Zhang X., Tian T., Sun W., Liu C., Fang X. (2017). Bmi-1 overexpression as an efficient prognostic marker in patients with nonsmall cell lung cancer. Medicine.

[347] Lang Y., Kong X., He C., Wang F., Liu B., Zhang S., Ning J., Zhu K., Xu S. (2017). Musashi1 Promotes Non-Small Cell Lung Carcinoma Malignancy and Chemoresistance via Activating the Akt Signaling Pathway. Cell Physiol. Biochem..

[348] Kumar M., Jaiswal R.K., Prasad R., Yadav S.S., Kumar A., Yadava P.K., Singh R.P. (2021). PARP-1 induces EMT in non-small cell lung carcinoma cells via modulating the transcription factors Smad4, p65 and ZEB1. Life Sci..

[349] Jafarian A.H., Kooshki Forooshani M., Reisi H., Mohamadian Roshan N. (2020). Matrix metalloproteinase-9 (MMP-9) Expression in Non-Small Cell Lung Carcinoma and Its Association with Clinicopathologic Factors. Iran. J. Pathol..

[350] Merchant N., Nagaraju G.P., Rajitha B., Lammata S., Jella K.K., Buchwald Z.S., Lakka S.S., Ali A.N. (2017). Matrix metalloproteinases: Their functional role in lung cancer. Carcinogenesis.

[351] Byers L.A., Heymach J.V. (2007). Dual targeting of the vascular endothelial growth factor and epidermal growth factor receptor pathways: Rationale and clinical applications for non-small-cell lung cancer. Clin. Lung Cancer.

[352] Otsuka S., Bebb G. (2008). The CXCR4/SDF-1 chemokine receptor axis: A new target therapeutic for non-small cell lung cancer. J. Thorac. Oncol..

[353] Kristiansen G., Schluns K., Yongwei Y., Denkert C., Dietel M., Petersen I. (2003). CD24 is an independent prognostic marker of survival in nonsmall cell lung cancer patients. Br. J. Cancer.

[354] Hu B., Ma Y., Yang Y., Zhang L., Han H., Chen J. (2018). CD44 promotes cell proliferation in non-small cell lung cancer. Oncol. Lett..

[355] Tirino V., Camerlingo R., Franco R., Malanga D., La Rocca A., Viglietto G., Rocco G., Pirozzi G. (2009). The role of CD133 in the identification and characterisation of tumour-initiating cells in non-small-cell lung cancer. Eur. J. Cardiothorac. Surg..

[356] Ko T.Y., Kim J.I., Lee S.H. (2020). Relationship between Cancer Stem Cell Marker CD133 and Cancer Germline Antigen Genes in NCI-H292 Lung Cancer Cells. Korean J. Thorac. Cardiovasc. Surg..

[357] Kim W.Y., Oh S.H., Woo J.K., Hong W.K., Lee H.Y. (2009). Targeting heat shock protein 90 overrides the resistance of lung cancer cells by blocking radiation-induced stabilization of hypoxia-inducible factor-1alpha. Cancer Res..

[358] Akakura N., Kobayashi M., Horiuchi I., Suzuki A., Wang J., Chen J., Niizeki H., Kawamura K., Hosokawa M., Asaka M. (2001). Constitutive expression of hypoxia-inducible factor-1alpha renders pancreatic cancer cells resistant to apoptosis induced by hypoxia and nutrient deprivation. Cancer Res..

[359] Kim J.Y., Kim H.J., Jung C.W., Lee T.S., Kim E.H., Park M.J. (2021). CXCR4 uses STAT3-mediated slug expression to maintain radioresistance of non-small cell lung cancer cells: Emerges as a potential prognostic biomarker for lung cancer. Cell Death Dis..

[360] Loriot Y., Mordant P., Dorvault N., De la motte Rouge T., Bourhis J., Soria J.C., Deutsch E. (2010). BMS-690514, a VEGFR and EGFR tyrosine kinase inhibitor, shows anti-tumoural activity on non-small-cell lung cancer xenografts and induces sequence-dependent synergistic effect with radiation. Br. J. Cancer.

[361] Jiang S., Wang R., Yan H., Jin L., Dou X., Chen D. (2016). MicroRNA-21 modulates radiation resistance through upregulation of hypoxia-inducible factor-1alpha-promoted glycolysis in non-small cell lung cancer cells. Mol. Med. Rep..

[362] Grosso S., Doyen J., Parks S.K., Bertero T., Paye A., Cardinaud B., Gounon P., Lacas-Gervais S., Noel A., Pouyssegur J. (2013). MiR-210 promotes a hypoxic phenotype and increases radioresistance in human lung cancer cell lines. Cell Death Dis..

[363] He Z., Liu Y., Xiao B., Qian X. (2015). miR-25 modulates NSCLC cell radio-sensitivity through directly inhibiting BTG2 expression. Biochem. Biophys. Res. Commun..

[364] Li Y., Han W., Ni T.T., Lu L., Huang M., Zhang Y., Cao H., Zhang H.Q., Luo W., Li H. (2015). Knockdown of microRNA-1323 restores sensitivity to radiation by suppression of PRKDC activity in radiation-resistant lung cancer cells. Oncol. Rep..

[365] Lee Y.S., Oh J.H., Yoon S., Kwon M.S., Song C.W., Kim K.H., Cho M.J., Mollah M.L., Je Y.J., Kim Y.D. (2010). Differential gene expression profiles of radioresistant non-small-cell lung cancer cell lines established by fractionated irradiation: Tumor protein p53-inducible protein 3 confers sensitivity to ionizing radiation. Int. J. Radiat. Oncol. Biol. Phys..

[366] Davidson B., Goldberg I., Kopolovic J., Lerner-Geva L., Gotlieb W.H., Ben-Baruch G., Reich R. (1999). MMP-2 and TIMP-2 expression correlates with poor prognosis in cervical carcinoma--a clinicopathologic study using immunohistochemistry and mRNA in situ hybridization. Gynecol. Oncol..

[367] Tsutsumi K., Tsuda M., Yazawa N., Nakamura H., Ishihara S., Haga H., Yasuda M., Yamazaki R., Shirato H., Kawaguchi H. (2009). Increased motility and invasiveness in tumor cells that survive 10 Gy irradiation. Cell Struct. Funct..

[368] Heo W., Lee Y.S., Son C.H., Yang K., Park Y.S., Bae J. (2015). Radiation-induced matrix metalloproteinases limit natural killer cell-mediated anticancer immunity in NCI-H23 lung cancer cells. Mol. Med. Rep..

[369] Zhuang X., Qiao T., Xu G., Yuan S., Zhang Q., Chen X. (2016). Combination of nadroparin with radiotherapy results in powerful synergistic antitumor effects in lung adenocarcinoma A549 cells. Oncol. Rep..

[370] Swinson D.E., Jones J.L., Cox G., Richardson D., Harris A.L., O’Byrne K.J. (2004). Hypoxia-inducible factor-1 alpha in non small cell lung cancer: Relation to growth factor, protease and apoptosis pathways. Int. J. Cancer.

[371] Hussenet T., Dali S., Exinger J., Monga B., Jost B., Dembele D., Martinet N., Thibault C., Huelsken J., Brambilla E. (2010). SOX2 is an oncogene activated by recurrent 3q26.3 amplifications in human lung squamous cell carcinomas. PLoS ONE.

[372] Bass A.J., Watanabe H., Mermel C.H., Yu S., Perner S., Verhaak R.G., Kim S.Y., Wardwell L., Tamayo P., Gat-Viks I. (2009). SOX2 is an amplified lineage-survival oncogene in lung and esophageal squamous cell carcinomas. Nat. Genet..

[373] Chowdhury P., Dey P., Ghosh S., Sarma A., Ghosh U. (2019). Reduction of metastatic potential by inhibiting EGFR/Akt/p38/ERK signaling pathway and epithelial-mesenchymal transition after carbon ion exposure is potentiated by PARP-1 inhibition in non-small-cell lung cancer. BMC Cancer.

[374] Guster J.D., Weissleder S.V., Busch C.J., Kriegs M., Petersen C., Knecht R., Dikomey E., Rieckmann T. (2014). The inhibition of PARP but not EGFR results in the radiosensitization of HPV/p16-positive HNSCC cell lines. Radiother. Oncol..

[375] Toulany M., Iida M., Keinath S., Iyi F.F., Mueck K., Fehrenbacher B., Mansour W.Y., Schaller M., Wheeler D.L., Rodemann H.P. (2016). Dual targeting of PI3K and MEK enhances the radiation response of K-RAS mutated non-small cell lung cancer. Oncotarget.

[376] Kang J., Kim E., Kim W., Seong K.M., Youn H., Kim J.W., Kim J., Youn B. (2013). Rhamnetin and cirsiliol induce radiosensitization and inhibition of epithelial-mesenchymal transition (EMT) by miR-34a-mediated suppression of Notch-1 expression in non-small cell lung cancer cell lines. J. Biol. Chem..

[377] Tian Y., Liu Q., He X., Yuan X., Chen Y., Chu Q., Wu K. (2016). Emerging roles of Nrf2 signal in non-small cell lung cancer. J. Hematol. Oncol..

[378] Wu D., Li L., Yan W. (2016). Knockdown of TC-1 enhances radiosensitivity of non-small cell lung cancer via the Wnt/beta-catenin pathway. Biol. Open.

[379] Zeng J., Aziz K., Chettiar S.T., Aftab B.T., Armour M., Gajula R., Gandhi N., Salih T., Herman J.M., Wong J. (2013). Hedgehog pathway inhibition radiosensitizes non-small cell lung cancers. Int. J. Radiat. Oncol. Biol. Phys..

[380] Heavey S., O’Byrne K.J., Gately K. (2014). Strategies for co-targeting the PI3K/AKT/mTOR pathway in NSCLC. Cancer Treat. Rev..

[381] Zhang T., Cui G.B., Zhang J., Zhang F., Zhou Y.A., Jiang T., Li X.F. (2010). Inhibition of PI3 kinases enhances the sensitivity of non-small cell lung cancer cells to ionizing radiation. Oncol. Rep..

[382] Zagouras P., Stifani S., Blaumueller C.M., Carcangiu M.L., Artavanis-Tsakonas S. (1995). Alterations in Notch signaling in neoplastic lesions of the human cervix. Proc. Natl. Acad. Sci. USA.

[383] Grabher C., von Boehmer H., Look A.T. (2006). Notch 1 activation in the molecular pathogenesis of T-cell acute lymphoblastic leukaemia. Nat. Rev. Cancer.

[384] Theys J., Yahyanejad S., Habets R., Span P., Dubois L., Paesmans K., Kattenbeld B., Cleutjens J., Groot A.J., Schuurbiers O.C.J. (2013). High NOTCH activity induces radiation resistance in non small cell lung cancer. Radiother. Oncol..

[385] Zou B., Zhou X.L., Lai S.Q., Liu J.C. (2018). Notch signaling and non-small cell lung cancer. Oncol. Lett..

[386] Eliasz S., Liang S., Chen Y., De Marco M.A., Machek O., Skucha S., Miele L., Bocchetta M. (2010). Notch-1 stimulates survival of lung adenocarcinoma cells during hypoxia by activating the IGF-1R pathway. Oncogene.

[387] Zhao Q., Mao A., Yan J., Sun C., Di C., Zhou X., Li H., Guo R., Zhang H. (2016). Downregulation of Nrf2 promotes radiation-induced apoptosis through Nrf2 mediated Notch signaling in non-small cell lung cancer cells. Int. J. Oncol..

[388] Chen Y.H., Pan S.L., Wang J.C., Kuo S.H., Cheng J.C., Teng C.M. (2014). Radiation-induced VEGF-C expression and endothelial cell proliferation in lung cancer. Strahlenther. Onkol..

[389] Jiang L., Huang J., Hu Y., Lu P., Luo Q., Wang L. (2020). Gli promotes tumor progression through regulating epithelial-mesenchymal transition in non-small-cell lung cancer. J. Cardiothorac. Surg..

[390] Garcia Campelo M.R., Alonso Curbera G., Aparicio Gallego G., Grande Pulido E., Anton Aparicio L.M. (2011). Stem cell and lung cancer development: Blaming the Wnt, Hh and Notch signalling pathway. Clin. Transl. Oncol..

[391] Pustovalova M., Alhaddad L., Blokhina T., Smetanina N., Chigasova A., Chuprov-Netochin R., Eremin P., Gilmutdinova I., Osipov A.N., Leonov S. (2021). The CD44high Subpopulation of Multifraction Irradiation-Surviving NSCLC Cells Exhibits Partial EMT-Program Activation and DNA Damage Response Depending on Their p53 Status. Int. J. Mol. Sci..

[392] Cao Z., Livas T., Kyprianou N. (2016). Anoikis and EMT: Lethal "Liaisons" during Cancer Progression. Crit. Rev. Oncog..

[393] Thiery J.P., Acloque H., Huang R.Y., Nieto M.A. (2009). Epithelial-mesenchymal transitions in development and disease. Cell.

[394] Lah T.T., Novak M., Breznik B. (2020). Brain malignancies: Glioblastoma and brain metastases. Semin. Cancer Biol..

[395] Mittal V. (2018). Epithelial Mesenchymal Transition in Tumor Metastasis. Annu. Rev. Pathol..

[396] Das R., Gregory P.A., Hollier B.G., Tilley W.D., Selth L.A. (2014). Epithelial plasticity in prostate cancer: Principles and clinical perspectives. Trends Mol. Med..

[397] Ansieau S., Bastid J., Doreau A., Morel A.P., Bouchet B.P., Thomas C., Fauvet F., Puisieux I., Doglioni C., Piccinin S. (2008). Induction of EMT by twist proteins as a collateral effect of tumor-promoting inactivation of premature senescence. Cancer Cell.

[398] Odero-Marah V., Hawsawi O., Henderson V., Sweeney J. (2018). Epithelial-Mesenchymal Transition (EMT) and Prostate Cancer. Adv. Exp. Med. Biol..

[399] Radisky E.S., Radisky D.C. (2010). Matrix metalloproteinase-induced epithelial-mesenchymal transition in breast cancer. J. Mammary Gland Biol. Neoplasia.

[400] Lu W., Kang Y. (2019). Epithelial-Mesenchymal Plasticity in Cancer Progression and Metastasis. Dev. Cell.

[401] Jolly M.K., Mani S.A., Levine H. (2018). Hybrid epithelial/mesenchymal phenotype(s): The ‘fittest’ for metastasis?. Biochim. Biophys. Acta Rev. Cancer.

[402] Gonzalez D.M., Medici D. (2014). Signaling mechanisms of the epithelial-mesenchymal transition. Sci. Signal..

[403] Huber M.A., Kraut N., Beug H. (2005). Molecular requirements for epithelial-mesenchymal transition during tumor progression. Curr. Opin. Cell Biol..

[404] Peinado H., Olmeda D., Cano A. (2007). Snail, Zeb and bHLH factors in tumour progression: An alliance against the epithelial phenotype?. Nat. Rev. Cancer.

[405] Kuner R., Muley T., Meister M., Ruschhaupt M., Buness A., Xu E.C., Schnabel P., Warth A., Poustka A., Sultmann H. (2009). Global gene expression analysis reveals specific patterns of cell junctions in non-small cell lung cancer subtypes. Lung Cancer.

[406] Pierdomenico M., Palone F., Cesi V., Vitali R., Mancuso A.B., Cucchiara S., Oliva S., Aloi M., Stronati L. (2018). Transcription Factor ZNF281: A Novel Player in Intestinal Inflammation and Fibrosis. Front. Immunol..

[407] De Craene B., Berx G. (2013). Regulatory networks defining EMT during cancer initiation and progression. Nat. Rev. Cancer.

[408] Boelens M.C., van den Berg A., Vogelzang I., Wesseling J., Postma D.S., Timens W., Groen H.J. (2007). Differential expression and distribution of epithelial adhesion molecules in non-small cell lung cancer and normal bronchus. J. Clin. Pathol..

[409] Scheel C., Weinberg R.A. (2012). Cancer stem cells and epithelial-mesenchymal transition: Concepts and molecular links. Semin. Cancer Biol..

[410] Kawamoto A., Yokoe T., Tanaka K., Saigusa S., Toiyama Y., Yasuda H., Inoue Y., Miki C., Kusunoki M. (2012). Radiation induces epithelial-mesenchymal transition in colorectal cancer cells. Oncol. Rep..

[411] Andarawewa K.L., Erickson A.C., Chou W.S., Costes S.V., Gascard P., Mott J.D., Bissell M.J., Barcellos-Hoff M.H. (2007). Ionizing radiation predisposes nonmalignant human mammary epithelial cells to undergo transforming growth factor beta induced epithelial to mesenchymal transition. Cancer Res..

[412] Zhang Y.E. (2009). Non-Smad pathways in TGF-beta signaling. Cell Res..

[413] Xu J., Lamouille S., Derynck R. (2009). TGF-beta-induced epithelial to mesenchymal transition. Cell Res..

[414] Jin W. (2020). Role of JAK/STAT3 Signaling in the Regulation of Metastasis, the Transition of Cancer Stem Cells, and Chemoresistance of Cancer by Epithelial-Mesenchymal Transition. Cells.

[415] Smit M.A., Peeper D.S. (2010). Epithelial-mesenchymal transition and senescence: Two cancer-related processes are crossing paths. Aging.

[416] Scheel C., Eaton E.N., Li S.H., Chaffer C.L., Reinhardt F., Kah K.J., Bell G., Guo W., Rubin J., Richardson A.L. (2011). Paracrine and autocrine signals induce and maintain mesenchymal and stem cell states in the breast. Cell.

[417] Chang L., Graham P.H., Hao J., Bucci J., Cozzi P.J., Kearsley J.H., Li Y. (2014). Emerging roles of radioresistance in prostate cancer metastasis and radiation therapy. Cancer Metastasis Rev..

[418] Marie-Egyptienne D.T., Lohse I., Hill R.P. (2013). Cancer stem cells, the epithelial to mesenchymal transition (EMT) and radioresistance: Potential role of hypoxia. Cancer Lett..

[419] Yao Y.H., Cui Y., Qiu X.N., Zhang L.Z., Zhang W., Li H., Yu J.M. (2016). Attenuated LKB1-SIK1 signaling promotes epithelial-mesenchymal transition and radioresistance of non-small cell lung cancer cells. Chin. J. Cancer.

[420] Heddleston J.M., Li Z., McLendon R.E., Hjelmeland A.B., Rich J.N. (2009). The hypoxic microenvironment maintains glioblastoma stem cells and promotes reprogramming towards a cancer stem cell phenotype. Cell Cycle.

[421] Salnikov A.V., Liu L., Platen M., Gladkich J., Salnikova O., Ryschich E., Mattern J., Moldenhauer G., Werner J., Schemmer P. (2012). Hypoxia induces EMT in low and highly aggressive pancreatic tumor cells but only cells with cancer stem cell characteristics acquire pronounced migratory potential. PLoS ONE.

[422] Brabletz S., Brabletz T. (2010). The ZEB/miR-200 feedback loop--a motor of cellular plasticity in development and cancer?. EMBO Rep..

[423] Tripathi S.C., Peters H.L., Taguchi A., Katayama H., Wang H., Momin A., Jolly M.K., Celiktas M., Rodriguez-Canales J., Liu H. (2016). Immunoproteasome deficiency is a feature of non-small cell lung cancer with a mesenchymal phenotype and is associated with a poor outcome. Proc. Natl. Acad. Sci. USA.

[424] Chen L., Gibbons D.L., Goswami S., Cortez M.A., Ahn Y.H., Byers L.A., Zhang X., Yi X., Dwyer D., Lin W. (2014). Metastasis is regulated via microRNA-200/ZEB1 axis control of tumour cell PD-L1 expression and intratumoral immunosuppression. Nat. Commun..

[425] Creighton C.J., Chang J.C., Rosen J.M. (2010). Epithelial-mesenchymal transition (EMT) in tumor-initiating cells and its clinical implications in breast cancer. J. Mammary Gland Biol. Neoplasia.

[426] Vega S., Morales A.V., Ocana O.H., Valdes F., Fabregat I., Nieto M.A. (2004). Snail blocks the cell cycle and confers resistance to cell death. Genes Dev..

[427] Comaills V., Kabeche L., Morris R., Buisson R., Yu M., Madden M.W., LiCausi J.A., Boukhali M., Tajima K., Pan S. (2016). Genomic Instability Is Induced by Persistent Proliferation of Cells Undergoing Epithelial-to-Mesenchymal Transition. Cell Rep..

[428] Liu M., Quek L.E., Sultani G., Turner N. (2016). Epithelial-mesenchymal transition induction is associated with augmented glucose uptake and lactate production in pancreatic ductal adenocarcinoma. Cancer Metab..

[429] Cha Y.H., Yook J.I., Kim H.S., Kim N.H. (2015). Catabolic metabolism during cancer EMT. Arch. Pharm. Res..

[430] Chamberlain M.C. (2011). Radiographic patterns of relapse in glioblastoma. J. Neurooncol..

[431] Bremnes R.M., Al-Shibli K., Donnem T., Sirera R., Al-Saad S., Andersen S., Stenvold H., Camps C., Busund L.T. (2011). The role of tumor-infiltrating immune cells and chronic inflammation at the tumor site on cancer development, progression, and prognosis: Emphasis on non-small cell lung cancer. J. Thorac. Oncol..

[432] Lee S.Y., Jeong E.K., Ju M.K., Jeon H.M., Kim M.Y., Kim C.H., Park H.G., Han S.I., Kang H.S. (2017). Induction of metastasis, cancer stem cell phenotype, and oncogenic metabolism in cancer cells by ionizing radiation. Mol. Cancer.

[433] Cho J.H., Hong W.G., Jung Y.J., Lee J., Lee E., Hwang S.G., Um H.D., Park J.K. (2016). Gamma-Ionizing radiation-induced activation of the EGFR-p38/ERK-STAT3/CREB-1-EMT pathway promotes the migration/invasion of non-small cell lung cancer cells and is inhibited by podophyllotoxin acetate. Tumour Biol..

[434] Thiery J.P. (2002). Epithelial-mesenchymal transitions in tumour progression. Nat. Rev. Cancer.

[435] Guarino M., Rubino B., Ballabio G. (2007). The role of epithelial-mesenchymal transition in cancer pathology. Pathology.

[436] Wild-Bode C., Weller M., Rimner A., Dichgans J., Wick W. (2001). Sublethal irradiation promotes migration and invasiveness of glioma cells: Implications for radiotherapy of human glioblastoma. Cancer Res..

[437] Bensimon J., Altmeyer-Morel S., Benjelloun H., Chevillard S., Lebeau J. (2013). CD24(-/low) stem-like breast cancer marker defines the radiation-resistant cells involved in memorization and transmission of radiation-induced genomic instability. Oncogene.

[438] Lin J.C., Tsai J.T., Chao T.Y., Ma H.I., Liu W.H. (2018). The STAT3/Slug Axis Enhances Radiation-Induced Tumor Invasion and Cancer Stem-like Properties in Radioresistant Glioblastoma. Cancers.

[439] Shintani Y., Okimura A., Sato K., Nakagiri T., Kadota Y., Inoue M., Sawabata N., Minami M., Ikeda N., Kawahara K. (2011). Epithelial to mesenchymal transition is a determinant of sensitivity to chemoradiotherapy in non-small cell lung cancer. Ann. Thorac. Surg..

[440] Kim E., Youn H., Kwon T., Son B., Kang J., Yang H.J., Seong K.M., Kim W., Youn B. (2014). PAK1 tyrosine phosphorylation is required to induce epithelial-mesenchymal transition and radioresistance in lung cancer cells. Cancer Res..

[441] Alhaddad L., Pustovalova M., Blokhina T., Chuprov-Netochin R., Osipov A.N., Leonov S. (2021). IR-Surviving NSCLC Cells Exhibit Different Patterns of Molecular and Cellular Reactions Relating to the Multifraction Irradiation Regimen and p53-Family Proteins Expression. Cancers.

[442] Saleh T., Carpenter V.J., Bloukh S., Gewirtz D.A. (2022). Targeting tumor cell senescence and polyploidy as potential therapeutic strategies. Semin. Cancer Biol..

[443] Zhang J., Si J., Gan L., Di C., Xie Y., Sun C., Li H., Guo M., Zhang H. (2019). Research progress on therapeutic targeting of quiescent cancer cells. Artif. Cells Nanomed. Biotechnol..

[444] Ewald J.A., Desotelle J.A., Wilding G., Jarrard D.F. (2010). Therapy-induced senescence in cancer. J. Natl. Cancer Inst..

[445] Aguirre-Ghiso J.A. (2007). Models, mechanisms and clinical evidence for cancer dormancy. Nat. Rev. Cancer.

[446] Triana-Martinez F., Loza M.I., Dominguez E. (2020). Beyond Tumor Suppression: Senescence in Cancer Stemness and Tumor Dormancy. Cells.

[447] Rao S.G., Jackson J.G. (2016). SASP: Tumor Suppressor or Promoter? Yes!. Trends Cancer.

[448] Sharpless N.E., Sherr C.J. (2015). Forging a signature of in vivo senescence. Nat. Rev. Cancer.

[449] Santos-de-Frutos K., Djouder N. (2021). When dormancy fuels tumour relapse. Commun. Biol..

[450] Edelstein J.E. (1988). Prosthetic feet. State of the Art. Phys. Ther..

[451] Crea F., Nur Saidy N.R., Collins C.C., Wang Y. (2015). The epigenetic/noncoding origin of tumor dormancy. Trends Mol. Med..

[452] Meacham C.E., Morrison S.J. (2013). Tumour heterogeneity and cancer cell plasticity. Nature.

[453] Mohammad K., Dakik P., Medkour Y., Mitrofanova D., Titorenko V.I. (2019). Quiescence Entry, Maintenance, and Exit in Adult Stem Cells. Int. J. Mol. Sci..

[454] Coller H.A., Sang L., Roberts J.M. (2006). A new description of cellular quiescence. PLoS Biol..

[455] Krenning L., van den Berg J., Medema R.H. (2019). Life or Death after a Break: What Determines the Choice?. Mol. Cell.

[456] Shaltiel I.A., Krenning L., Bruinsma W., Medema R.H. (2015). The same, only different—DNA damage checkpoints and their reversal throughout the cell cycle. J. Cell Sci..

[457] Puig I., Tenbaum S.P., Chicote I., Arques O., Martinez-Quintanilla J., Cuesta-Borras E., Ramirez L., Gonzalo P., Soto A., Aguilar S. (2018). TET2 controls chemoresistant slow-cycling cancer cell survival and tumor recurrence. J. Clin. Investig..

[458] Wolter K., Zender L. (2020). Therapy-induced senescence—An induced synthetic lethality in liver cancer?. Nat. Rev. Gastroenterol. Hepatol..

[459] Masunaga S., Sakurai Y., Tanaka H., Hirayama R., Matsumoto Y., Uzawa A., Suzuki M., Kondo N., Narabayashi M., Maruhashi A. (2013). Radiosensitivity of pimonidazole-unlabelled intratumour quiescent cell population to gamma-rays, accelerated carbon ion beams and boron neutron capture reaction. Br. J. Radiol..

[460] Miller I., Min M., Yang C., Tian C., Gookin S., Carter D., Spencer S.L. (2018). Ki67 is a Graded Rather than a Binary Marker of Proliferation versus Quiescence. Cell Rep..

[461] Zeniou M., Feve M., Mameri S., Dong J., Salome C., Chen W., El-Habr E.A., Bousson F., Sy M., Obszynski J. (2015). Chemical Library Screening and Structure-Function Relationship Studies Identify Bisacodyl as a Potent and Selective Cytotoxic Agent Towards Quiescent Human Glioblastoma Tumor Stem-Like Cells. PLoS ONE.

[462] Yao M., Xie C., Kiang M.Y., Teng Y., Harman D., Tiffen J., Wang Q., Sved P., Bao S., Witting P. (2015). Targeting of cytosolic phospholipase A2alpha impedes cell cycle re-entry of quiescent prostate cancer cells. Oncotarget.

[463] Kwon J.S., Everetts N.J., Wang X., Wang W., Della Croce K., Xing J., Yao G. (2017). Controlling Depth of Cellular Quiescence by an Rb-E2F Network Switch. Cell Rep..

[464] Pei H., Guo Z., Wang Z., Dai Y., Zheng L., Zhu L., Zhang J., Hu W., Nie J., Mao W. (2018). RAC2 promotes abnormal proliferation of quiescent cells by enhanced JUNB expression via the MAL-SRF pathway. Cell Cycle.

[465] Chitikova Z.V., Gordeev S.A., Bykova T.V., Zubova S.G., Pospelov V.A., Pospelova T.V. (2014). Sustained activation of DNA damage response in irradiated apoptosis-resistant cells induces reversible senescence associated with mTOR downregulation and expression of stem cell markers. Cell Cycle.

[466] Collado M., Gil J., Efeyan A., Guerra C., Schuhmacher A.J., Barradas M., Benguría A., Zaballos A., Flores J.M., Barbacid M. (2005). Senescence in premalignant tumours. Nature.

[467] He G., Dhar D., Nakagawa H., Font-Burgada J., Ogata H., Jiang Y., Shalapour S., Seki E., Yost S.E., Jepsen K. (2013). Identification of Liver Cancer Progenitors Whose Malignant Progression Depends on Autocrine IL-6 Signaling. Cell.

[468] Joselow A., Lynn D., Terzian T., Box N.F. (2017). Senescence-Like Phenotypes in Human Nevi. Methods Mol. Biol..

[469] Haugstetter A.M., Loddenkemper C., Lenze D., Gröne J., Standfuß C., Petersen I., Dörken B., Schmitt C.A. (2010). Cellular senescence predicts treatment outcome in metastasised colorectal cancer. Br. J. Cancer.

[470] Kahlem P., Dörken B., Schmitt C.A. (2004). Cellular senescence in cancer treatment: Friend or foe?. J. Clin. Investig..

[471] Schmitt C.A., Fridman J.S., Yang M., Lee S., Baranov E., Hoffman R.M., Lowe S.W. (2002). A Senescence Program Controlled by p53 and p16INK4a Contributes to the Outcome of Cancer Therapy. Cell.

[472] Serrano M., Lin A.W., McCurrach M.E., Beach D., Lowe S.W. (1997). Oncogenic ras provokes premature cell senescence associated with accumulation of p53 and p16INK4a. Cell.

[473] Mikula-Pietrasik J., Niklas A., Uruski P., Tykarski A., Ksiazek K. (2020). Mechanisms and significance of therapy-induced and spontaneous senescence of cancer cells. Cell Mol. Life Sci..

[474] Razmik Mirzayans D.M. (2013). Role of Therapy-Induced Cellular Senescence in Tumor Cells and its Modification in Radiotherapy: The Good, The Bad and The Ugly. J. Nucl. Med. Radiat. Ther..

[475] Lee B.Y., Han J.A., Im J.S., Morrone A., Johung K., Goodwin E.C., Kleijer W.J., DiMaio D., Hwang E.S. (2006). Senescence-associated β-galactosidase is lysosomal β-galactosidase. Aging Cell.

[476] Ogrodnik M., Miwa S., Tchkonia T., Tiniakos D., Wilson C.L., Lahat A., Day C.P., Burt A., Palmer A., Anstee Q.M. (2017). Cellular senescence drives age-dependent hepatic steatosis. Nat. Commun..

[477] Kaushik S., Cuervo A.M. (2015). Proteostasis and aging. Nat. Med..

[478] Hernandez-Segura A., de Jong T.V., Melov S., Guryev V., Campisi J., Demaria M. (2017). Unmasking Transcriptional Heterogeneity in Senescent Cells. Curr. Biol..

[479] Suzuki M., Boothman D.A. (2008). Stress-induced Premature Senescence (SIPS). J. Radiat. Res..

[480] Saleh T., Tyutynuk-Massey L., Cudjoe E.K., Idowu M.O., Landry J.W., Gewirtz D.A. (2018). Non-Cell Autonomous Effects of the Senescence-Associated Secretory Phenotype in Cancer Therapy. Front. Oncol..

[481] Antonangeli F., Soriani A., Ricci B., Ponzetta A., Benigni G., Morrone S., Bernardini G., Santoni A. (2016). Natural killer cell recognition of in vivo drug-induced senescent multiple myeloma cells. OncoImmunology.

[482] Coppé J.-P., Desprez P.-Y., Krtolica A., Campisi J. (2010). The Senescence-Associated Secretory Phenotype: The Dark Side of Tumor Suppression. Annu. Rev. Pathol. Mech. Dis..

[483] Hansel C., Jendrossek V., Klein D. (2020). Cellular Senescence in the Lung: The Central Role of Senescent Epithelial Cells. Int. J. Mol. Sci..

[484] Coppe J.P., Patil C.K., Rodier F., Sun Y., Munoz D.P., Goldstein J., Nelson P.S., Desprez P.Y., Campisi J. (2008). Senescence-associated secretory phenotypes reveal cell-nonautonomous functions of oncogenic RAS and the p53 tumor suppressor. PLoS Biol..

[485] Passos J.F., Nelson G., Wang C., Richter T., Simillion C., Proctor C.J., Miwa S., Olijslagers S., Hallinan J., Wipat A. (2010). Feedback between p21 and reactive oxygen production is necessary for cell senescence. Mol. Syst. Biol..

[486] Wiley C.D., Velarde M.C., Lecot P., Liu S., Sarnoski E.A., Freund A., Shirakawa K., Lim H.W., Davis S.S., Ramanathan A. (2016). Mitochondrial Dysfunction Induces Senescence with a Distinct Secretory Phenotype. Cell Metab..

[487] Chang B.D., Broude E.V., Dokmanovic M., Zhu H., Ruth A., Xuan Y., Kandel E.S., Lausch E., Christov K., Roninson I.B. (1999). A senescence-like phenotype distinguishes tumor cells that undergo terminal proliferation arrest after exposure to anticancer agents. Cancer Res..

[488] Dimri G.P., Lee X., Basile G., Acosta M., Scott G., Roskelley C., Medrano E.E., Linskens M., Rubelj I., Pereira-Smith O. (1995). A biomarker that identifies senescent human cells in culture and in aging skin in vivo. Proc. Natl. Acad. Sci. USA.

[489] Campisi J. (2001). Cellular senescence as a tumor-suppressor mechanism. Trends Cell Biol..

[490] Chien Y., Scuoppo C., Wang X., Fang X., Balgley B., Bolden J.E., Premsrirut P., Luo W., Chicas A., Lee C.S. (2011). Control of the senescence-associated secretory phenotype by NF-kappaB promotes senescence and enhances chemosensitivity. Genes Dev..

[491] Aird K.M., Zhang R. (2013). Detection of senescence-associated heterochromatin foci (SAHF). Methods Mol. Biol..

[492] Ksiazek K., Korybalska K., Jorres A., Witowski J. (2007). Accelerated senescence of human peritoneal mesothelial cells exposed to high glucose: The role of TGF-beta1. Lab. Investig..

[493] Krouwer V.J., Hekking L.H., Langelaar-Makkinje M., Regan-Klapisz E., Post J.A. (2012). Endothelial cell senescence is associated with disrupted cell-cell junctions and increased monolayer permeability. Vasc. Cell.

[494] Statuto M., Bianchi C., Perego R., Del Monte U. (2002). Drop of connexin 43 in replicative senescence of human fibroblasts HEL-299 as a possible biomarker of senescence. Exp. Gerontol..

[495] Wang Q., Wu P.C., Dong D.Z., Ivanova I., Chu E., Zeliadt S., Vesselle H., Wu D.Y. (2013). Polyploidy road to therapy-induced cellular senescence and escape. Int. J. Cancer.

[496] Saleh T., Tyutyunyk-Massey L., Murray G.F., Alotaibi M.R., Kawale A.S., Elsayed Z., Henderson S.C., Yakovlev V., Elmore L.W., Toor A. (2019). Tumor cell escape from therapy-induced senescence. Biochem. Pharm..

[497] Yang L., Fang J., Chen J. (2017). Tumor cell senescence response produces aggressive variants. Cell Death Discov..

[498] Ghorai A., Mahaddalkar T., Thorat R., Dutt S. (2020). Sustained inhibition of PARP-1 activity delays glioblastoma recurrence by enhancing radiation-induced senescence. Cancer Lett..

[499] Cahu J., Bustany S., Sola B. (2012). Senescence-associated secretory phenotype favors the emergence of cancer stem-like cells. Cell Death Dis..

[500] Ye C., Zhang X., Wan J., Chang L., Hu W., Bing Z., Zhang S., Li J., He J., Wang J. (2013). Radiation-induced cellular senescence results from a slippage of long-term G2 arrested cells into G1 phase. Cell Cycle.

[501] He J., Li J., Ye C., Zhou L., Zhu J., Wang J., Mizota A., Furusawa Y., Zhou G. (2011). Cell cycle suspension: A novel process lurking in G(2) arrest. Cell Cycle.

[502] Sugrue M.M., Shin D.Y., Lee S.W., Aaronson S.A. (1997). Wild-type p53 triggers a rapid senescence program in human tumor cells lacking functional p53. Proc. Natl. Acad. Sci. USA.

[503] Calio A., Zamo A., Ponzoni M., Zanolin M.E., Ferreri A.J., Pedron S., Montagna L., Parolini C., Fraifeld V.E., Wolfson M. (2015). Cellular Senescence Markers p16INK4a and p21CIP1/WAF Are Predictors of Hodgkin Lymphoma Outcome. Clin. Cancer Res..

[504] Gorgoulis V., Adams P.D., Alimonti A., Bennett D.C., Bischof O., Bishop C., Campisi J., Collado M., Evangelou K., Ferbeyre G. (2019). Cellular Senescence: Defining a Path Forward. Cell.

[505] Evangelou K., Lougiakis N., Rizou S.V., Kotsinas A., Kletsas D., Munoz-Espin D., Kastrinakis N.G., Pouli N., Marakos P., Townsend P. (2017). Robust, universal biomarker assay to detect senescent cells in biological specimens. Aging Cell.

[506] Liao E.C., Hsu Y.T., Chuah Q.Y., Lee Y.J., Hu J.Y., Huang T.C., Yang P.M., Chiu S.J. (2014). Radiation induces senescence and a bystander effect through metabolic alterations. Cell Death Dis..

[507] Roninson I.B. (2003). Tumor cell senescence in cancer treatment. Cancer Res..

[508] Jeon H.Y., Kim J.K., Ham S.W., Oh S.Y., Kim J., Park J.B., Lee J.Y., Kim S.C., Kim H. (2016). Irradiation induces glioblastoma cell senescence and senescence-associated secretory phenotype. Tumour Biol..

[509] Quick Q.A., Gewirtz D.A. (2006). An accelerated senescence response to radiation in wild-type p53 glioblastoma multiforme cells. J. Neurosurg..

[510] Luo H., Yount C., Lang H., Yang A., Riemer E.C., Lyons K., Vanek K.N., Silvestri G.A., Schulte B.A., Wang G.Y. (2013). Activation of p53 with Nutlin-3a radiosensitizes lung cancer cells via enhancing radiation-induced premature senescence. Lung Cancer.

[511] Jones K.R., Elmore L.W., Jackson-Cook C., Demasters G., Povirk L.F., Holt S.E., Gewirtz D.A. (2005). p53-Dependent accelerated senescence induced by ionizing radiation in breast tumour cells. Int. J. Radiat. Biol..

[512] Roberson R.S., Kussick S.J., Vallieres E., Chen S.Y., Wu D.Y. (2005). Escape from therapy-induced accelerated cellular senescence in p53-null lung cancer cells and in human lung cancers. Cancer Res..

[513] Wang Q., Wu P.C., Roberson R.S., Luk B.V., Ivanova I., Chu E., Wu D.Y. (2011). Survivin and escaping in therapy-induced cellular senescence. Int. J. Cancer.

[514] Azad A., Bukczynska P., Jackson S., Haupt Y., Cullinane C., McArthur G.A., Solomon B. (2014). Co-targeting deoxyribonucleic acid-dependent protein kinase and poly(adenosine diphosphate-ribose) polymerase-1 promotes accelerated senescence of irradiated cancer cells. Int. J. Radiat. Oncol. Biol. Phys..

[515] Williams B.R., Amon A. (2009). Aneuploidy: Cancer’s fatal flaw?. Cancer Res..

[516] Sudo T., Nitta M., Saya H., Ueno N.T. (2004). Dependence of paclitaxel sensitivity on a functional spindle assembly checkpoint. Cancer Res..

[517] Jordan M.A., Wilson L. (2004). Microtubules as a target for anticancer drugs. Nat. Rev. Cancer.

[518] Zhang Z., Feng X., Deng Z., Cheng J., Wang Y., Zhao M., Zhao Y., He S., Huang Q. (2021). Irradiation-induced polyploid giant cancer cells are involved in tumor cell repopulation via neosis. Mol. Oncol..

[519] Rand C.W., Courville C.B. (1947). Multinucleation of cortical nerve cells at the margins of traumatic lesions of the human brain. J. Neuropathol. Exp. Neurol..

[520] Liu J. (2018). The dualistic origin of human tumors. Semin. Cancer Biol..

[521] Barok M., Tanner M., Koninki K., Isola J. (2011). Trastuzumab-DM1 causes tumour growth inhibition by mitotic catastrophe in trastuzumab-resistant breast cancer cells in vivo. Breast Cancer Res..

[522] Lin K.C., Torga G., Sun Y., Axelrod R., Pienta K.J., Sturm J.C., Austin R.H. (2019). The role of heterogeneous environment and docetaxel gradient in the emergence of polyploid, mesenchymal and resistant prostate cancer cells. Clin. Exp. Metastasis.

[523] Ogden A., Rida P.C., Knudsen B.S., Kucuk O., Aneja R. (2015). Docetaxel-induced polyploidization may underlie chemoresistance and disease relapse. Cancer Lett..

[524] Zhang S., Zhang D., Yang Z., Zhang X. (2016). Tumor Budding, Micropapillary Pattern, and Polyploidy Giant Cancer Cells in Colorectal Cancer: Current Status and Future Prospects. Stem Cells Int..

[525] Fei F., Zhang M., Li B., Zhao L., Wang H., Liu L., Li Y., Ding P., Gu Y., Zhang X. (2019). Formation of Polyploid Giant Cancer Cells Involves in the Prognostic Value of Neoadjuvant Chemoradiation in Locally Advanced Rectal Cancer. J. Oncol..

[526] Zhang S., Mercado-Uribe I., Xing Z., Sun B., Kuang J., Liu J. (2014). Generation of cancer stem-like cells through the formation of polyploid giant cancer cells. Oncogene.

[527] Fujiwara T., Bandi M., Nitta M., Ivanova E.V., Bronson R.T., Pellman D. (2005). Cytokinesis failure generating tetraploids promotes tumorigenesis in p53-null cells. Nature.

[528] Revesz L., Norman U. (1960). Chromosome ploidy and radiosensitivity of tumours. Nature.

[529] Castedo M., Coquelle A., Vitale I., Vivet S., Mouhamad S., Viaud S., Zitvogel L., Kroemer G. (2006). Selective resistance of tetraploid cancer cells against DNA damage-induced apoptosis. Ann. N. Y. Acad. Sci..

[530] Ianzini F., Kosmacek E.A., Nelson E.S., Napoli E., Erenpreisa J., Kalejs M., Mackey M.A. (2009). Activation of meiosis-specific genes is associated with depolyploidization of human tumor cells following radiation-induced mitotic catastrophe. Cancer Res..

[531] Prieur-Carrillo G., Chu K., Lindqvist J., Dewey W.C. (2003). Computerized video time-lapse (CVTL) analysis of the fate of giant cells produced by X-irradiating EJ30 human bladder carcinoma cells. Radiat Res..

[532] Ianzini F., Mackey M.A. (2002). Development of the large scale digital cell analysis system. Radiat. Prot. Dosim..

[533] Erenpreisa J.A., Cragg M.S., Fringes B., Sharakhov I., Illidge T.M. (2000). Release of mitotic descendants by giant cells from irradiated Burkitt’s lymphoma cell line. Cell Biol. Int..

[534] Horbay R., Stoika R. (2011). Giant cell formation: The way to cell death or cell survival?. Open Life Sci..

[535] Sliwinska M.A., Mosieniak G., Wolanin K., Babik A., Piwocka K., Magalska A., Szczepanowska J., Fronk J., Sikora E. (2009). Induction of senescence with doxorubicin leads to increased genomic instability of HCT116 cells. Mech. Ageing Dev..

[536] Niu N., Mercado-Uribe I., Liu J. (2017). Dedifferentiation into blastomere-like cancer stem cells via formation of polyploid giant cancer cells. Oncogene.

[537] Erenpreisa J., Salmina K., Huna A., Jackson T.R., Vazquez-Martin A., Cragg M.S. (2015). The "virgin birth", polyploidy, and the origin of cancer. Oncoscience.

[538] Zhang D., Yang X., Yang Z., Fei F., Li S., Qu J., Zhang M., Li Y., Zhang X., Zhang S. (2017). Daughter Cells and Erythroid Cells Budding from PGCCs and Their Clinicopathological Significances in Colorectal Cancer. J. Cancer.

[539] Hosaka M., Hatori M., Smith R., Kokubun S. (2004). Giant cell formation through fusion of cells derived from a human giant cell tumor of tendon sheath. J. Orthop. Sci..

[540] Brodbeck W.G., Anderson J.M. (2009). Giant cell formation and function. Curr. Opin. Hematol..

[541] Holland A.J., Cleveland D.W. (2009). Boveri revisited: Chromosomal instability, aneuploidy and tumorigenesis. Nat. Rev. Mol. Cell Biol..

[542] Krajcovic M., Overholtzer M. (2012). Mechanisms of ploidy increase in human cancers: A new role for cell cannibalism. Cancer Res..

[543] Erenpreisa J., Cragg M.S. (2013). Three steps to the immortality of cancer cells: Senescence, polyploidy and self-renewal. Cancer Cell Int..

[544] Beermann W. (1964). Control of Differentiation at the Chromosomal Level. J. Exp. Zool..

[545] Erenpreisa J., Cragg M.S., Anisimov A.P., Illidge T.M. (2011). Tumor cell embryonality and the ploidy number 32n: Is it a developmental checkpoint?. Cell Cycle.

[546] Vakifahmetoglu H., Olsson M., Zhivotovsky B. (2008). Death through a tragedy: Mitotic catastrophe. Cell Death Differ..

[547] Kaur E., Rajendra J., Jadhav S., Shridhar E., Goda J.S., Moiyadi A., Dutt S. (2015). Radiation-induced homotypic cell fusions of innately resistant glioblastoma cells mediate their sustained survival and recurrence. Carcinogenesis.

[548] Sundaram M., Guernsey D.L., Rajaraman M.M., Rajaraman R. (2004). Neosis: A novel type of cell division in cancer. Cancer Biol. Ther..

[549] Erenpreisa J., Cragg M.S. (2001). Mitotic death: A mechanism of survival? A review. Cancer Cell Int..

[550] Niu N., Zhang J., Zhang N., Mercado-Uribe I., Tao F., Han Z., Pathak S., Multani A.S., Kuang J., Yao J. (2016). Linking genomic reorganization to tumor initiation via the giant cell cycle. Oncogenesis.

[551] Erenpreisa J., Ivanov A., Wheatley S.P., Kosmacek E.A., Ianzini F., Anisimov A.P., Mackey M., Davis P.J., Plakhins G., Illidge T.M. (2008). Endopolyploidy in irradiated p53-deficient tumour cell lines: Persistence of cell division activity in giant cells expressing Aurora-B kinase. Cell Biol. Int..

[552] Erenpreisa J., Cragg M.S., Salmina K., Hausmann M., Scherthan H. (2009). The role of meiotic cohesin REC8 in chromosome segregation in gamma irradiation-induced endopolyploid tumour cells. Exp. Cell Res..

[553] Vitale I., Senovilla L., Jemaa M., Michaud M., Galluzzi L., Kepp O., Nanty L., Criollo A., Rello-Varona S., Manic G. (2010). Multipolar mitosis of tetraploid cells: Inhibition by p53 and dependency on Mos. EMBO J..

[554] Rajaraman R., Guernsey D.L., Rajaraman M.M., Rajaraman S.R. (2006). Stem cells, senescence, neosis and self-renewal in cancer. Cancer Cell Int..

[555] Rajaraman R., Rajaraman M.M., Rajaraman S.R., Guernsey D.L. (2005). Neosis--a paradigm of self-renewal in cancer. Cell Biol. Int..

[556] White-Gilbertson S., Voelkel-Johnson C. (2020). Giants and monsters: Unexpected characters in the story of cancer recurrence. Adv. Cancer Res..

[557] Diaz-Carballo D., Saka S., Klein J., Rennkamp T., Acikelli A.H., Malak S., Jastrow H., Wennemuth G., Tempfer C., Schmitz I. (2018). A Distinct Oncogenerative Multinucleated Cancer Cell Serves as a Source of Stemness and Tumor Heterogeneity. Cancer Res..

[558] Puck T.T., Marcus P.I. (1956). Action of x-rays on mammalian cells. J. Exp. Med..

[559] Liang B.C., Thornton A.F., Sandler H.M., Greenberg H.S. (1991). Malignant astrocytomas: Focal tumor recurrence after focal external beam radiation therapy. J. Neurosurg..

[560] Sneed P.K., Gutin P.H., Larson D.A., Malec M.K., Phillips T.L., Prados M.D., Scharfen C.O., Weaver K.A., Wara W.M. (1994). Patterns of recurrence of glioblastoma multiforme after external irradiation followed by implant boost. Int. J. Radiat. Oncol. Biol. Phys..

[561] Sitarz R., Leguit R.J., de Leng W.W., Morsink F.H., Polkowski W.P., Maciejewski R., Offerhaus G.J., Milne A.N. (2009). Cyclooxygenase-2 mediated regulation of E-cadherin occurs in conventional but not early-onset gastric cancer cell lines. Cell Oncol..

[562] Hoskin D.W., Mader J.S., Furlong S.J., Conrad D.M., Blay J. (2008). Inhibition of T cell and natural killer cell function by adenosine and its contribution to immune evasion by tumor cells (Review). Int. J. Oncol..

[563] Malik S.T., Griffin D.B., Fiers W., Balkwill F.R. (1989). Paradoxical effects of tumour necrosis factor in experimental ovarian cancer. Int. J. Cancer.

[564] Sa G., Das T., Moon C., Hilston C.M., Rayman P.A., Rini B.I., Tannenbaum C.S., Finke J.H. (2009). GD3, an overexpressed tumor-derived ganglioside, mediates the apoptosis of activated but not resting T cells. Cancer Res..

[565] Toutirais O., Chartier P., Dubois D., Bouet F., Leveque J., Catros-Quemener V., Genetet N. (2003). Constitutive expression of TGF-beta1, interleukin-6 and interleukin-8 by tumor cells as a major component of immune escape in human ovarian carcinoma. Eur. Cytokine Netw..

[566] Inoue K., Slaton J.W., Kim S.J., Perrotte P., Eve B.Y., Bar-Eli M., Radinsky R., Dinney C.P. (2000). Interleukin 8 expression regulates tumorigenicity and metastasis in human bladder cancer. Cancer Res..

[567] Doubrovina E.S., Doubrovin M.M., Vider E., Sisson R.B., O’Reilly R.J., Dupont B., Vyas Y.M. (2003). Evasion from NK cell immunity by MHC class I chain-related molecules expressing colon adenocarcinoma. J. Immunol..

[568] Mannino M., Chalmers A.J. (2011). Radioresistance of glioma stem cells: Intrinsic characteristic or property of the ’microenvironment-stem cell unit’?. Mol. Oncol..

[569] Gabrilovich D., Ishida T., Oyama T., Ran S., Kravtsov V., Nadaf S., Carbone D.P. (1998). Vascular endothelial growth factor inhibits the development of dendritic cells and dramatically affects the differentiation of multiple hematopoietic lineages in vivo. Blood.

[570] Whiteside T.L., Vujanovic N.L., Herberman R.B. (1998). Natural killer cells and tumor therapy. Curr. Top. Microbiol. Immunol..

[571] Vivier E., Tomasello E., Baratin M., Walzer T., Ugolini S. (2008). Functions of natural killer cells. Nat. Immunol..

[572] Kaufman H.L., Wolchok J.D. (2007). General Principles of Tumor Immunotherapy.

[573] Baay M., Brouwer A., Pauwels P., Peeters M., Lardon F. (2011). Tumor cells and tumor-associated macrophages: Secreted proteins as potential targets for therapy. Clin. Dev. Immunol..

[574] Tong H., Ke J.Q., Jiang F.Z., Wang X.J., Wang F.Y., Li Y.R., Lu W., Wan X.P. (2016). Tumor-associated macrophage-derived CXCL8 could induce ERalpha suppression via HOXB13 in endometrial cancer. Cancer Lett..

[575] Augello A., Tasso R., Negrini S.M., Amateis A., Indiveri F., Cancedda R., Pennesi G. (2005). Bone marrow mesenchymal progenitor cells inhibit lymphocyte proliferation by activation of the programmed death 1 pathway. Eur. J. Immunol..

[576] Di Nicola M., Carlo-Stella C., Magni M., Milanesi M., Longoni P.D., Matteucci P., Grisanti S., Gianni A.M. (2002). Human bone marrow stromal cells suppress T-lymphocyte proliferation induced by cellular or nonspecific mitogenic stimuli. Blood.

[577] Whiteside T.L. (2008). The tumor microenvironment and its role in promoting tumor growth. Oncogene.

[578] Caires H.R., Barros da Silva P., Barbosa M.A., Almeida C.R. (2018). A co-culture system with three different primary human cell populations reveals that biomaterials and MSC modulate macrophage-driven fibroblast recruitment. J. Tissue Eng. Regen. Med..

[579] Almand B., Clark J.I., Nikitina E., van Beynen J., English N.R., Knight S.C., Carbone D.P., Gabrilovich D.I. (2001). Increased production of immature myeloid cells in cancer patients: A mechanism of immunosuppression in cancer. J. Immunol..

[580] Leonard W., Dufait I., Schwarze J.K., Law K., Engels B., Jiang H., Van den Berge D., Gevaert T., Storme G., Verovski V. (2016). Myeloid-derived suppressor cells reveal radioprotective properties through arginase-induced l-arginine depletion. Radiother. Oncol..

[581] Ochoa A.C., Zea A.H., Hernandez C., Rodriguez P.C. (2007). Arginase, prostaglandins, and myeloid-derived suppressor cells in renal cell carcinoma. Clin. Cancer Res..

[582] Serafini P., Borrello I., Bronte V. (2006). Myeloid suppressor cells in cancer: Recruitment, phenotype, properties, and mechanisms of immune suppression. Semin. Cancer Biol..

[583] Munn D.H., Mellor A.L. (2007). Indoleamine 2,3-dioxygenase and tumor-induced tolerance. J. Clin. Investig..

[584] Loukinova E., Dong G., Enamorado-Ayalya I., Thomas G.R., Chen Z., Schreiber H., Van Waes C. (2000). Growth regulated oncogene-alpha expression by murine squamous cell carcinoma promotes tumor growth, metastasis, leukocyte infiltration and angiogenesis by a host CXC receptor-2 dependent mechanism. Oncogene.

[585] Sakaguchi S., Miyara M., Costantino C.M., Hafler D.A. (2010). FOXP3+ regulatory T cells in the human immune system. Nat. Rev. Immunol..

[586] Bergmann C., Strauss L., Zeidler R., Lang S., Whiteside T.L. (2007). Expansion of human T regulatory type 1 cells in the microenvironment of cyclooxygenase 2 overexpressing head and neck squamous cell carcinoma. Cancer Res..

[587] Colombo M.P., Piconese S. (2007). Regulatory-T-cell inhibition versus depletion: The right choice in cancer immunotherapy. Nat. Rev. Cancer.

[588] Arden K.C. (2008). FOXO animal models reveal a variety of diverse roles for FOXO transcription factors. Oncogene.

[589] Li C., Jiang P., Wei S., Xu X., Wang J. (2020). Regulatory T cells in tumor microenvironment: New mechanisms, potential therapeutic strategies and future prospects. Mol. Cancer.

[590] Kochetkova I., Golden S., Holderness K., Callis G., Pascual D.W. (2010). IL-35 stimulation of CD39+ regulatory T cells confers protection against collagen II-induced arthritis via the production of IL-10. J. Immunol..

[591] Kitahata Y., Shinkai M., Kido T. (1988). Guidance and nursing of a patient with acute myocardial infarction and senile dementia: A case study. Kango Gijutsu.

[592] Ihara F., Sakurai D., Takami M., Kamata T., Kunii N., Yamasaki K., Iinuma T., Nakayama T., Motohashi S., Okamoto Y. (2019). Regulatory T cells induce CD4(-) NKT cell anergy and suppress NKT cell cytotoxic function. Cancer Immunol. Immunother..

[593] Spolski R., Li P., Leonard W.J. (2018). Biology and regulation of IL-2: From molecular mechanisms to human therapy. Nat. Rev. Immunol..

[594] Ohta A., Kini R., Ohta A., Subramanian M., Madasu M., Sitkovsky M. (2012). The development and immunosuppressive functions of CD4(+) CD25(+) FoxP3(+) regulatory T cells are under influence of the adenosine-A2A adenosine receptor pathway. Front. Immunol..

[595] Raimondi G., Turner M.S., Thomson A.W., Morel P.A. (2007). Naturally occurring regulatory T cells: Recent insights in health and disease. Crit. Rev. Immunol..

[596] Roncarolo M.G., Gregori S., Battaglia M., Bacchetta R., Fleischhauer K., Levings M.K. (2006). Interleukin-10-secreting type 1 regulatory T cells in rodents and humans. Immunol. Rev..

[597] Baratelli F., Lin Y., Zhu L., Yang S.C., Heuze-Vourc’h N., Zeng G., Reckamp K., Dohadwala M., Sharma S., Dubinett S.M. (2005). Prostaglandin E2 induces FOXP3 gene expression and T regulatory cell function in human CD4+ T cells. J. Immunol..

[598] Brown N.F., Carter T.J., Ottaviani D., Mulholland P. (2018). Harnessing the immune system in glioblastoma. Br. J. Cancer.

[599] Abedalthagafi M., Barakeh D., Foshay K.M. (2018). Immunogenetics of glioblastoma: The future of personalized patient management. NPJ Precis. Oncol..

[600] Liang J., Piao Y., Holmes L., Fuller G.N., Henry V., Tiao N., de Groot J.F. (2014). Neutrophils promote the malignant glioma phenotype through S100A4. Clin. Cancer Res..

[601] Calabrese C., Poppleton H., Kocak M., Hogg T.L., Fuller C., Hamner B., Oh E.Y., Gaber M.W., Finklestein D., Allen M. (2007). A perivascular niche for brain tumor stem cells. Cancer Cell.

[602] Cui Y.H., Suh Y., Lee H.J., Yoo K.C., Uddin N., Jeong Y.J., Lee J.S., Hwang S.G., Nam S.Y., Kim M.J. (2015). Radiation promotes invasiveness of non-small-cell lung cancer cells through granulocyte-colony-stimulating factor. Oncogene.

